# A Portal‐Preorganized Cucurbit[7]uril‐Ruthenium Conjugate Enables Motif‐Selective Protein Sensing

**DOI:** 10.1002/anie.6821024

**Published:** 2026-09-08

**Authors:** Patrick Gruhs, Albert Kraft, Vahideh Mahram, Marco Neumaier, Papri Chakraborty, Emily Janz, Maria Vittoria Balli, Pierre Picchetti, Luca Prodi, Manfred Kappes, Frank Biedermann

**Affiliations:** ^1^ Institute of Nanotechnology (INT) Karlsruhe Institute of Technology (KIT) Karlsruhe Germany; ^2^ Department of Chemistry “Giacomo Ciamician” University of Bologna Bologna Italy

**Keywords:** emission‐based sensing, host–guest binding, protein recognition, supramolecular chemistry

## Abstract

We report a portal‐preorganized cucurbit[7]uril (CB7)‐ruthenium conjugate that reprograms CB7 recognition without modifying the host cavity itself. Covalent integration of a tris(2,2′‐bipyridine)ruthenium(II) chromophore at the CB7 portal yields the unimolecular receptor CB7‐Rubpy, in which guest binding is directly coupled to emission. The proximal Ru chromophore attenuates binding of classical polycationic CB7 guests while enhancing recognition of aromatic peptide and protein motifs, producing a selectivity shift of nearly two orders of magnitude relative to native CB7. CB7‐Rubpy thereby converts CB7 from a charge‐selective host into a motif‐selective receptor for aromatic protein epitopes, displaying high affinity for insulin (*K_a_* = 1.5 × 10^7^ M^−1^) and submicromolar detection capability. The same motif‐selective recognition enables monitoring of aromatic motif accessibility during protein refolding. These findings establish portal‐preorganized chromophore integration as a design strategy for coupling selectivity tuning and signal transduction in CB‐based receptors.

## Graphical abstract

Reprogramming cucurbit[7]uril (CB7) recognition through portal engineering transforms an optically silent host into a motif‐selective photoluminescent receptor. The portal‐preorganized CB7‐Rubpy conjugate directly reports changes in aromatic motif accessibility, enabling optical sensing of protein misfolding and refolding.

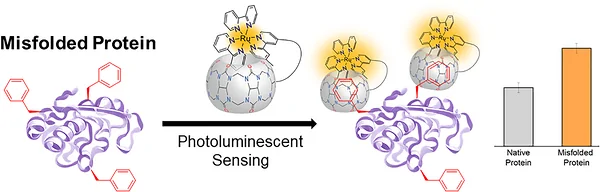

## 1 Introduction

Macrocyclic hosts are among the most powerful platforms for molecular recognition because their preorganized cavities enable selective binding through a combination of noncovalent interactions, including hydrophobic, dispersive, hydrogen‐bonding, and electrostatic contributions [1, 2, 3, 4, 5]. In aqueous media, binding is often further enhanced by the release of high‐energy water molecules from the host cavity [6, 7]. These properties have established macrocycles as versatile receptors for molecular recognition [8, 9, 10], sensing [11, 12, 13], imaging [14, 15], and supramolecular catalysis [16, 17].

Among synthetic hosts, cucurbit[n]urils (CBn) have emerged as particularly useful receptors for molecular recognition in water [18, 19, 20, 21]. These pumpkin‐shaped glycoluril macrocycles possess hydrophobic cavities flanked by carbonyl‐lined portals, creating a distinctive recognition environment that favors cationic and hydrophobic guests through a combination of ion–dipole interactions, hydrophobic effects, and geometric complementarity [4, 19, 22]. Among the CB*n* homologues, cucurbit[7]uril (CB7) is especially attractive because it combines exceptional binding affinities—reaching the attomolar regime for selected guests [23]—with good water solubility and chemical stability, making it a benchmark host for molecular recognition in complex biological environments [24, 25, 26]. Beyond guest binding, the confined CB7 cavity can substantially influence the physicochemical properties of encapsulated molecules [11, 27]. In particular, CB7 has been shown to modulate the photophysical behavior of chromophores, leading to changes in emission intensity, spectral position, and excited‐state lifetimes [19, 28, 29]. These effects have been widely exploited in supramolecular sensing platforms, including indicator displacement assays (IDAs), host–dye conjugates, and sensor arrays [24, 25, 30]. Notably, Urbach, Isaacs, and coworkers demonstrated that covalent attachment of tetramethylrhodamine to CB7 can produce a folded host–dye conjugate for direct sensing and cellular imaging [15]. However, such systems generally exploit the native recognition properties of CB7 rather than reprogramming its underlying selectivity.

Beyond molecular sensing applications, supramolecular host–guest chemistry has increasingly emerged as a powerful approach for probing biomolecular structure and function [31, 32, 33, 34, 35]. In contrast to many conventional biophysical techniques, which primarily report on global structural features, synthetic receptors can provide direct information on the accessibility of defined chemical motifs exposed on biomolecular surfaces [36, 37]. This capability is particularly attractive for studying protein conformational changes, as unfolding, misfolding [38, 39, 40], and structural recovery can alter the accessibility of specific molecular epitopes without necessarily producing large changes in global structure. Seminal work by Urbach, Kim, Scherman, and Nau established that CB*n* hosts can recognize amino acids, peptides, and proteins through defined cationic and aromatic residues [41, 42, 43, 44], including N‐terminal phenylalanine motifs. In particular, CB7 was shown to bind insulin through its accessible B‐chain N‐terminal phenylalanine residue (PheB1), demonstrating that cucurbituril‐based receptors can interrogate biologically relevant surface motifs on proteins [45]. These studies highlight the potential of CB7‐derived receptors for biomolecular recognition while simultaneously underscoring the importance of controlling and tuning their underlying selectivity.

Despite these advances, CB7‐based sensing applications remain constrained by the intrinsic recognition preferences of the host. The binding behavior of CB7 is largely governed by guest‐cavity complementarity, hydrophobic effects, and favorable interactions with cationic guests, making it challenging to selectively target specific biomolecular motifs [21]. Furthermore, many CB7‐based sensing systems rely on noncovalent reporter dyes, introducing additional equilibria that complicate signal transduction and reduce robustness in complex environments [11, 17, 21, 24, 25, 46, 47].

For many synthetic macrocycles, host‐guest recognition can be adjusted by peripheral or cavity‐directed functionalization [48, 49, 50, 51]. Cucurbit[*n*]urils, however, offer far fewer synthetically accessible modification handles, and systematic cavity modification remains difficult. This raises a central question for CB*n* chemistry: can their recognition behavior be rationally reprogrammed without chemically altering the host cavity itself? We reasoned that portal‐directed functionalization could provide a means to reshape the local recognition environment of CB7 and thereby generate emergent binding behavior beyond that encoded by the native host.

## 2 Results and Discussion

### 2.1 Design and Synthesis of the CB7‐Rubpy System

To test whether portal engineering can reprogram macrocyclic host recognition without altering the host cavity itself, we designed the portal‐preorganized conjugate CB7‐Rubpy, in which CB7 is covalently linked to a tris(2,2′‐bipyridine)ruthenium(II) chromophore. This architecture places the dicationic Ru unit directly at the CB7 portal, creating a secondary recognition environment adjacent to the native cavity. We expected this portal‐defined interface to disfavor classical polycationic CB7 guests through electrostatic repulsion while promoting CB7‐compatible aromatic peptide and protein motifs through secondary portal‐associated interactions. At the same time, the environment‐sensitive Ru emitter [52, 53] was designed to translate guest binding at the portal into a direct luminescence response without indicator displacement.

CB7‐Rubpy was synthesized by copper‐catalyzed azide–alkyne cycloaddition (CuAAC) of azide‐functionalized Ru‐N_3_ and monopropargylated CB7 (CB7‐OPr; Scheme 1). Ru‐N_3_ was prepared from cis‐bis(2,2′‐bipyridine)dichlororuthenium(II) in three steps, whereas CB7‐OPr was obtained by sequential hydroxylation and O‐alkylation of CB7 [19, 46]. The final click reaction afforded CB7‐Rubpy in 40% isolated yield. Full synthetic procedures and characterization are provided in the Supporting Information.

**SCHEME 1:**
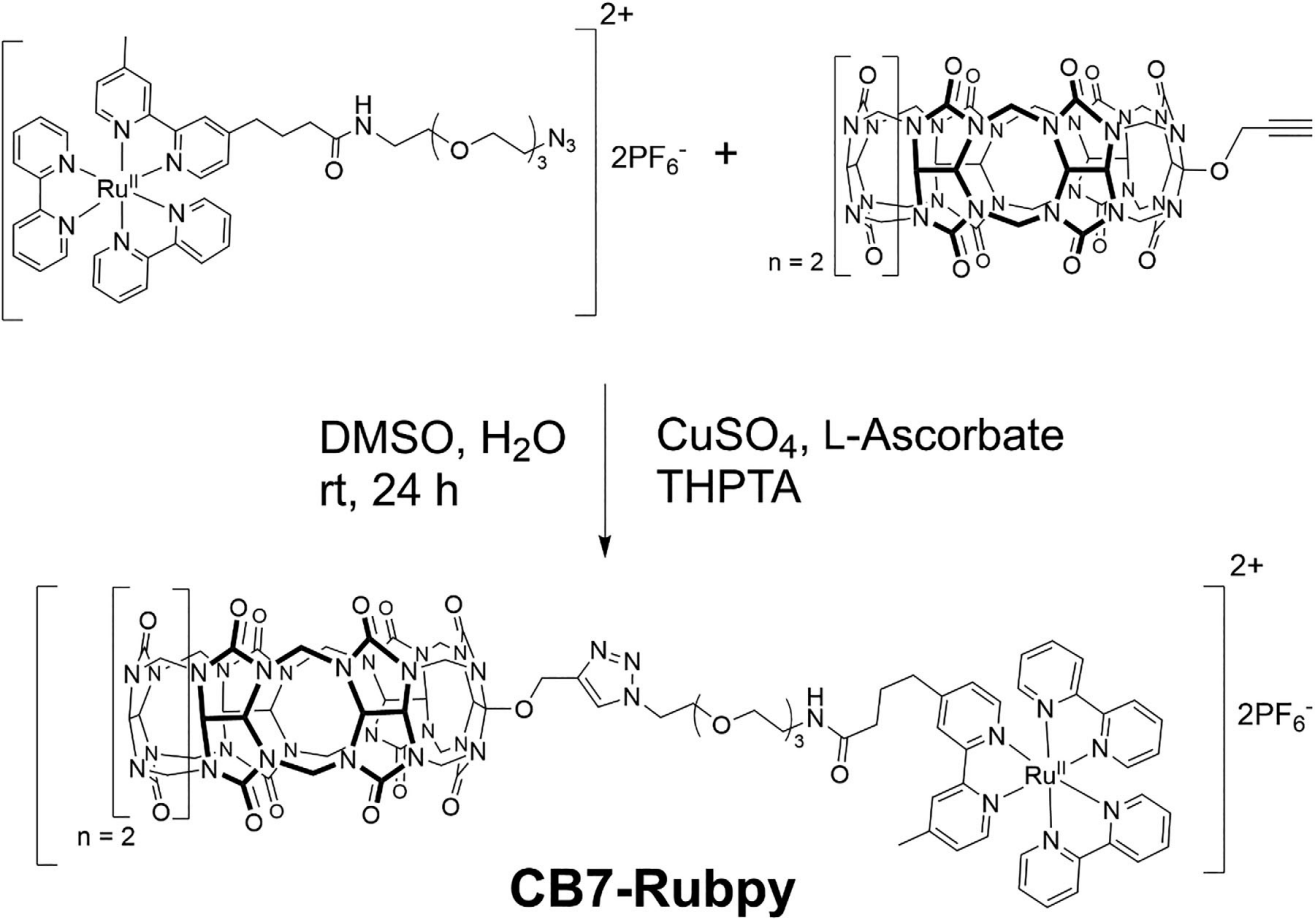
Synthesis of CB7‐Rubpy via a copper‐catalyzed azide–alkyne cycloaddition. Yield after flash chromatography on CHP20P resin is 40%.

### 2.2 Portal‐Preorganized CB7‐Rubpy Architecture

High‐resolution electrospray ionization mass spectrometry (HR‐ESI‐MS) confirmed the successful synthesis of CB7‐Rubpy. In the presence of the reporter guest [C_8_(mim)_2_]^2+^, a diagnostic peak at *m/z* = 590.72 (Figure S12) was observed, matching with the expected isotopologue distribution of the [CB7‐Rubpy•C_8_(mim)_2_]^4+^ complex. This confirms both the successful formation of CB7‐Rubpy and retention of CB7 host–guest recognition. Having established that CB7 binding is preserved after covalent functionalization, we next investigated whether portal engineering gives rise to a defined intramolecular architecture capable of influencing guest recognition. ^1^H NMR spectroscopy (Figure 1) provides the first direct evidence that CB7‐Rubpy adopts such an architecture. Successful CuAAC conjugation is confirmed by the diagnostic triazole resonance at δ 8.12 ppm (Figure 1c).

**FIGURE 1:**
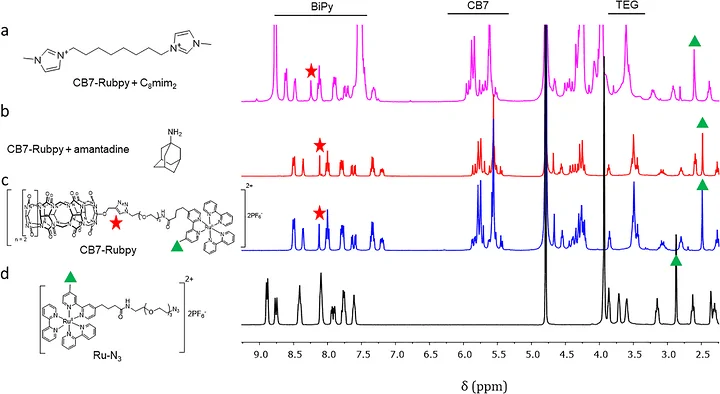
^1^H NMR spectra (400 MHz, D_2_O) of (a) CB7‐Rubpy (2 mM) with excess (10 equiv) C_8_mim_2_Br_2_ (magenta), (b) CB7‐Rubpy (2 mM) with excess (10 equiv) amantadine (red), (c) CB7‐Rubpy (2 mM, blue), and (d) the Ru‐N_3_ precursor (2 mM, black). The singlet at 8.12 ppm (red star) confirms triazole formation; the methyl group of the linking bipyridine ligand is indicated by a green triangle.

Relative to Ru‐N_3_ (Figure 1d), CB7‐Rubpy displays a uniform ∼0.5 ppm upfield shift of all bipyridine resonances, as well as of the methyl substituent on one bipyridine ligand (δ 2.87 → 2.49 ppm, Figure S18). In binary CB7 complexes, such shielding is typically indicative of proximity to the CB7 cavity [54]. However, control spectra of a binary mixture of CB7 and Ru‐COOH show no comparable shifts (Figure S19), excluding spontaneous noncovalent inclusion of [Ru(bpy)_3_]^2^^+^. Together with the ion‐mobility and computational results discussed below, these data rule out a deeply self‐included structure. Instead, the global shielding pattern is consistent with a folded, portal‐preorganized architecture in which the [Ru(bpy)_3_]^2^^+^ unit resides at the CB7 portal (Figures 2 and 3b).

**FIGURE 2:**
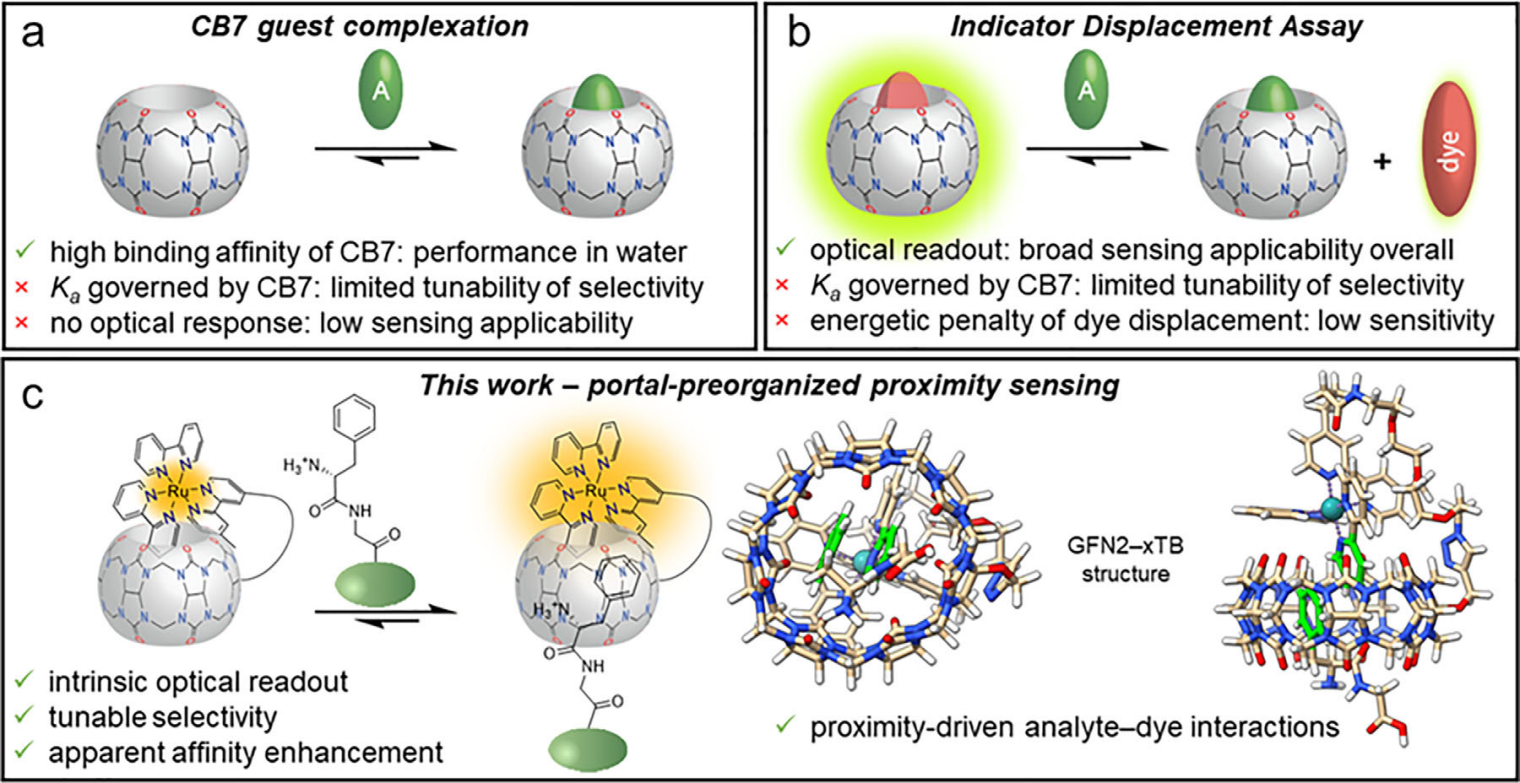
Comparison of simple CB7 host–guest systems, indicator displacement assays, and the CB7‐Rubpy sensing concept. (a) Host–guest complexation with CB7 provides high binding affinity but offers limited tunability of selectivity and lacks an intrinsic optical readout. (b) Indicator displacement assays introduce an optical response via a reporter dye. Their selectivity remains governed by the host, and signal generation requires dye displacement, which incurs an energetic penalty and limits sensitivity. (c) A covalently linked CB7‐Rubpy conjugate enforces portal‐proximal positioning of the chromophore, enabling intrinsic photoluminescent readout. This architecture establishes proximity‐driven sensing, in which analyte binding at the CB7 portal modulates the excited‐state properties of the Ru center. Secondary analyte‐chromophore interactions likely contribute to signal generation and allow modulation of selectivity and binding affinity.

**FIGURE 3:**
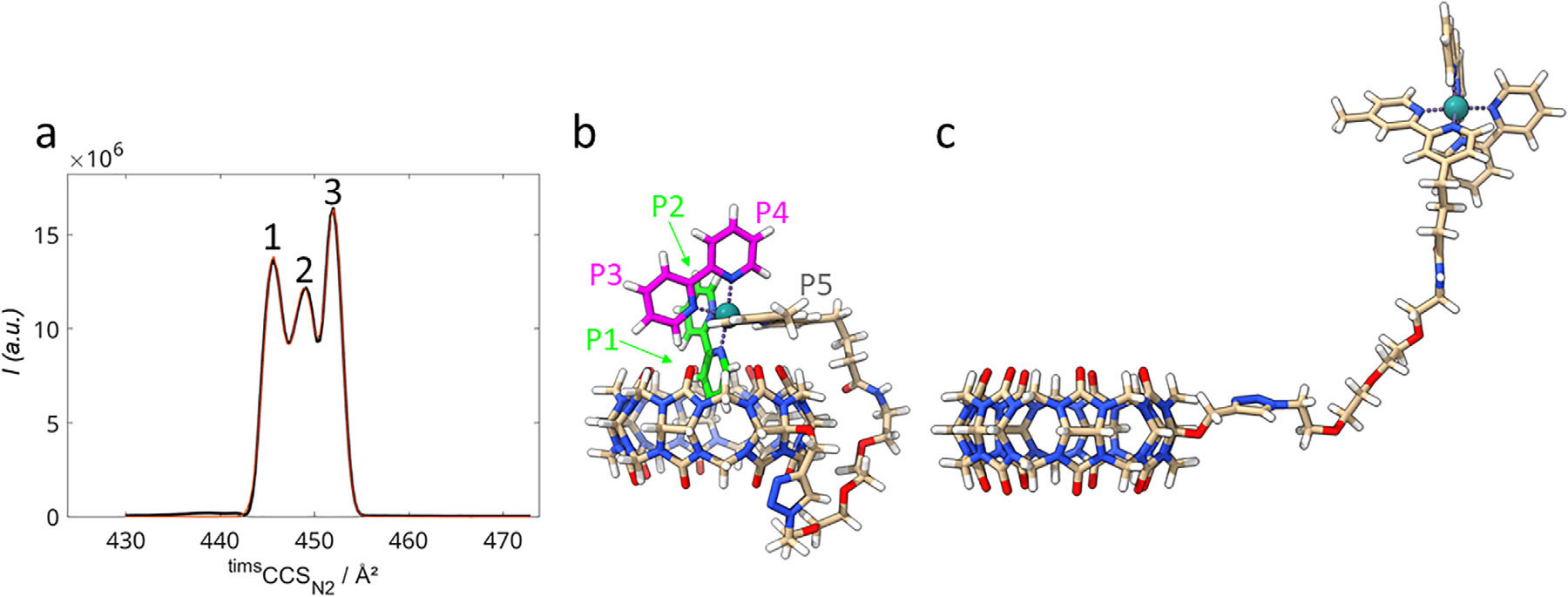
Ion mobility and GFN2‐xTB structural analysis of CB7‐Rubpy. (a) Ion mobility spectrum of [CB7‐Rubpy]^2^^+^ (3 kV electrospray voltage), showing three features with ^tims^CCS_N2_ values of 445.4 Å^2^ (1), 449.4 Å^2^ (2), and 453.0 Å^2^ (3). (b) GFN2‐xTB optimized structure of [CB7‐Rubpy]^2^^+^ highlighting accessible bipyridyl orientations (P1–P5), excluding the linker‐bound ring. (c) Representative extended conformation (GFN2‐xTB) with ^TM^CCS_N2_ = 626.0 Å^2^ (∼40% larger than experimentally observed) and Δ*E* = +4.56 eV relative to P1 (see Supporting Information); this structure is not observed experimentally and represents a purely computed limit, indicating that complete unfolding is not induced by guest binding. All calculated ^TM^CCS_N2_ values were scaled by a factor of 0.97 to achieve perfect agreement with the smallest of the three experimentally observed conformers.

To validate the proposed portal‐preorganized architecture, ion mobility spectrometry (IMS) and computational modeling were employed (Figure 3). IMS revealed three closely spaced conformational populations with ^tims^CCS_N2_ values of 445.4, 449.4, and 453.0 Å^2^ (Figure 3a), consistent with a compact dynamic ensemble. Computed low‐energy structures maintain close association of the Ru chromophore with the CB7 portal, and their calculated collision cross section (CCS) values agree well with experiment (Figures 3b and S20). By contrast, an open, extended architecture is incompatible with both the experimental CCS values and calculated energetics (Figure 3c). Thus, IMS and computation support a folded, portal‐preorganized ensemble in which the Ru chromophore remains positioned at the CB7 portal, creating the secondary recognition environment required for reprogramming CB7 selectivity.

### 2.3 Photophysical Properties

To assess whether portal preorganization affects the Ru chromophore, we compared the optical properties of CB7‐Rubpy and [Ru(bpy)_3_]^2+^ in water. Their nearly identical absorption spectra indicate that CB7 attachment does not substantially perturb the ground‐state electronic structure of the Ru complex (Figure S21). In contrast, CB7‐Rubpy shows a redshifted emission maximum (*λ*_max_ = 642 nm) relative to [Ru(bpy)_3_]^2+^ (*λ*_max_ = 624 nm), together with markedly reduced emission intensity (5 µM, Figure S22a). Thus, the portal‐preorganized architecture identified by NMR and IMS selectively perturbs the excited state of the Ru chromophore, providing the photophysical basis for signal generation.

Control experiments confirmed that this behavior requires portal preorganization. Binary mixtures of CB7 and [Ru(bpy)_3_]^2^^+^ showed no significant spectral changes even with up to 5 equiv. CB7 (Figure S22b,c), indicating that weak intermolecular host–chromophore interactions are insufficient to perturb the Ru excited state. In contrast, the model compound Ada‐Ru, in which adamantane binding enforces proximity between the Ru chromophore and the CB7 portal, reproduced the characteristic redshift and emission attenuation observed for CB7‐Rubpy upon CB7 addition (Figure S22b,d).

Together, these controls show that persistent portal proximity, not chromophore encapsulation or weak intermolecular association, is responsible for the altered Ru emission. Portal‐directed chromophore integration therefore converts the Ru unit from a reporter into part of the recognition interface, enabling guest binding to be translated directly into optical output.

### 2.4 Buffer and Metal Cation Effect on CB7‐Rubpy

We next examined whether the portal‐associated architecture of CB7‐Rubpy is sensitive to the ionic environment. While the absorption spectra remain essentially unchanged across different buffer systems, the emission response depends strongly on ionic strength. Emission spectra recorded in deionized water and phosphate buffer (PB, 10 mM, pH 7.4) are nearly identical (*λ*_max_ = 642 nm), whereas phosphate‐buffered saline (PBS, 1×, pH 7.4) produces a moderate increase in emission intensity together with a slight blueshift (*λ*_max_ = 639 nm, Figure S22a). These changes indicate that CB7‐Rubpy exists in a dynamic folded/open equilibrium that can be shifted by ions toward a more solvent‐exposed chromophore state. To probe this process directly, we investigated the effects of K^+^ and Na^+^, the major cations present in PB and PBS.

^1^H NMR titrations with KCl revealed a characteristic non‐monotonic response of the Rubpy methyl resonance, which shifted to a maximum at ≈32 mM KCl and returned to its initial position at ≈160 mM (Figure 4b, green triangle). In contrast, the aromatic bipyridine resonances and the triazole proton (Figure 4b, red star) remained largely unchanged up to ≈80 mM KCl and shifted only at higher concentrations (≥160 mM, Figure S23), consistent with global ionic‐strength effects. The Ru‐COOH control showed only minor monotonic salt‐induced shifts (Figure S24), confirming that the non‐monotonic response originates from the CB7‐Rubpy architecture rather than direct K^+^ effects on the Ru chromophore. These observations indicate a two‐stage reorganization of the host–chromophore interaction: initial K^+^ binding likely stabilizes the folded architecture at the free CB7 portal, whereas higher concentrations progressively disrupt the portal‐associated interaction. The Rubpy methyl resonance therefore serves as a local probe of the folded/open equilibrium.

**FIGURE 4:**
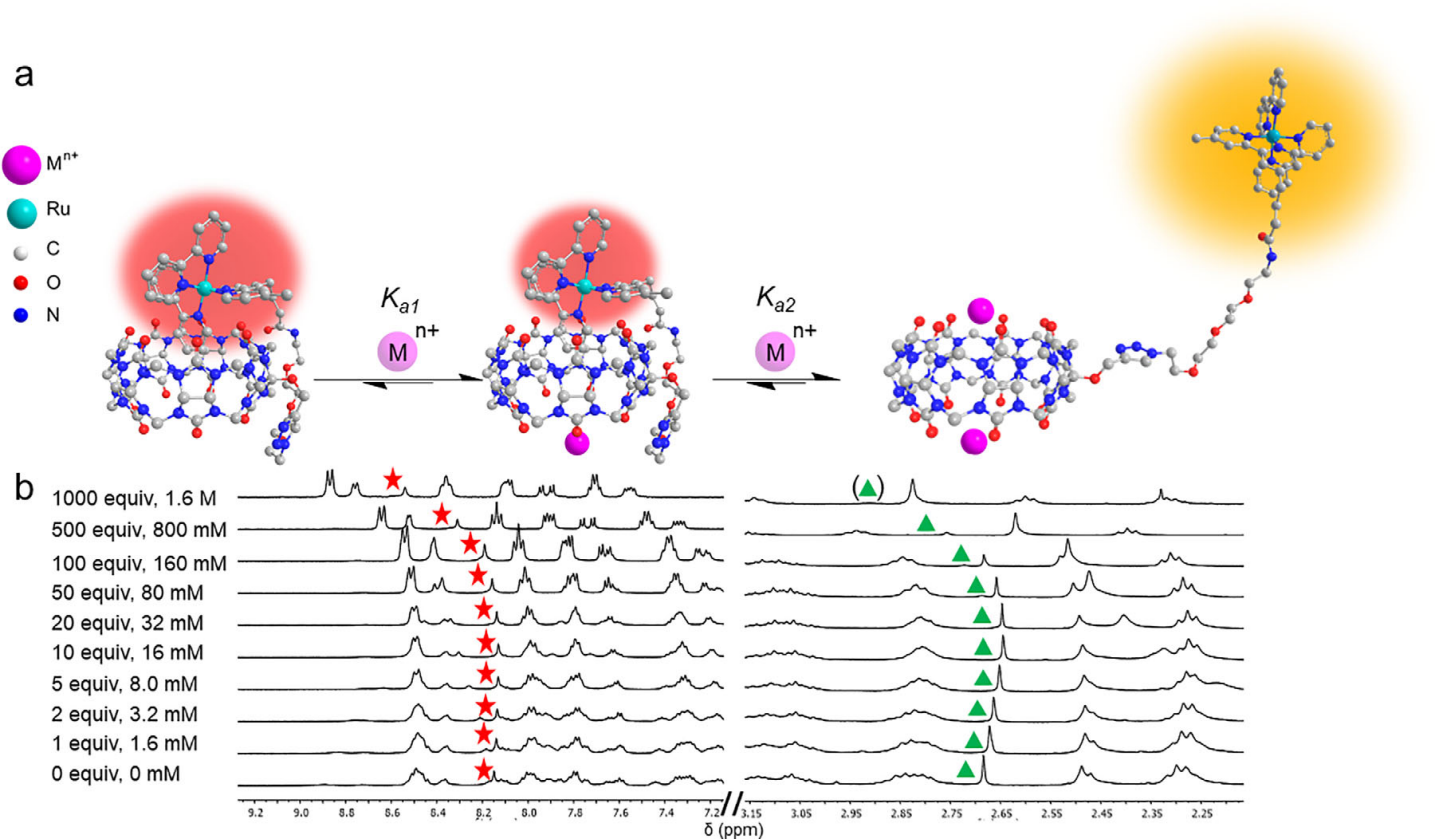
Salt‐dependent modulation of CB7‐Rubpy conformation and emission. (a) Schematic illustration of stepwise cation‐binding to CB7‐Rubpy. Metal cations (M^n^^+^) interact with the CB7 portals in a two‐stage binding process. As a result of cation binding to the free CB7 portal at low salt concentrations (≤ ∼10 mM), a decrease in [Ru(bpy)_3_]^2^^+^ emission intensity is observed. At higher salt concentrations, the CB7‐Rubpy portal interaction is disrupted in favor of a more solvent‐exposed chromophore state that shows a stronger and blueshifted emission characteristic of aqueous [Ru(bpy)_3_]^2^^+^. (b) ^1^H NMR titration of CB7‐Rubpy (1.6 mM) with increasing equivalents of KCl (400 MHz, D_2_O). The aromatic region (left) shows monotonic shifts with increasing ionic strength; the triazole proton (red star) serves as a reference signal and reflects global ionic strength effects. In contrast, the aliphatic region (right) reveals a non‐monotonic shift of the methyl group of the linker‐adjacent bipyridine ligand (green triangle), which first shifts downfield and then upfield with increasing KCl concentration, indicating local, salt‐dependent conformational changes.

Fitting the NMR data in the 0–80 mM KCl regime with a consecutive 2:1 binding model yielded estimated binding constants of *K_a1_* ≈ 130 M^−^^1^ and *K_a2_* ≈ 18 M^−1^ (Figure S25). Although the values should be interpreted cautiously because salt‐induced shifts overlap with the binding response, the trend is chemically meaningful: *K_a1_* is approximately one order of magnitude lower than for parent CB7 (Table S1), consistent with partial portal occupation and electrostatic repulsion by the proximal [Ru(bpy)_3_]^2+^ unit, whereas the still lower *K_a2_* reflects the energetic cost of displacing the portal‐associated chromophore.

Emission titrations with K^+^ and Na^+^ reproduced the same non‐monotonic two‐stage behavior observed by NMR (Figures S26–S29). Low salt concentrations caused a modest emission decrease (≈10%), consistent with an initial interaction regime in which the folded architecture remains largely intact. At higher concentrations, emission increased markedly and blueshifted toward the spectrum of [Ru(bpy)_3_]^2^^+^, indicating disruption of the intramolecular host–chromophore interaction and formation of a more solvent‐exposed Ru state. Fitting the high‐salt regime with a 1:1 model gave log*K_a__2_* = 0.64 ± 0.09 for K^+^ and 0.53 ± 0.09 for Na^+^, while Li^+^ had little effect (Figure S30). These values indicate that CB7‐Rubpy is predominantly folded in PB (> 95%), whereas PBS already contains a significant open‐state population (≈34%; folded ≈66%; see Supporting Information for population analysis). Thus, ionic strength directly controls the recognition‐active folded architecture and explains the reduced sensing response under physiological salt conditions.

Additional titrations with Mg^2^^+^, Gd^3^^+^, and Eu^3^^+^ showed the same qualitative two‐stage response (Figures S31–S35), confirming that the folded/open equilibrium is an intrinsic property of CB7‐Rubpy rather than specific to alkali–metal binding. Trivalent cations triggered the high‐concentration regime already at ≈10 mM, consistent with stronger portal binding due to their higher charge density.

### 2.5 Reprogrammed Host–Guest Recognition in CB7‐Rubpy

Having established CB7‐Rubpy as a dynamic, portal‐preorganized receptor, we next tested whether this secondary recognition environment alters the intrinsic guest selectivity of CB7. We examined a series of established CB7 guests, including amantadine (AdNH_2_), methyl viologen (M_2_V), spermine (Spm), spermidine (Spd), cadaverine (Cad), phenylalanine (Phe), and phenylalanyl‐glycine (Phe‐Gly; Figure 5).

**FIGURE 5:**
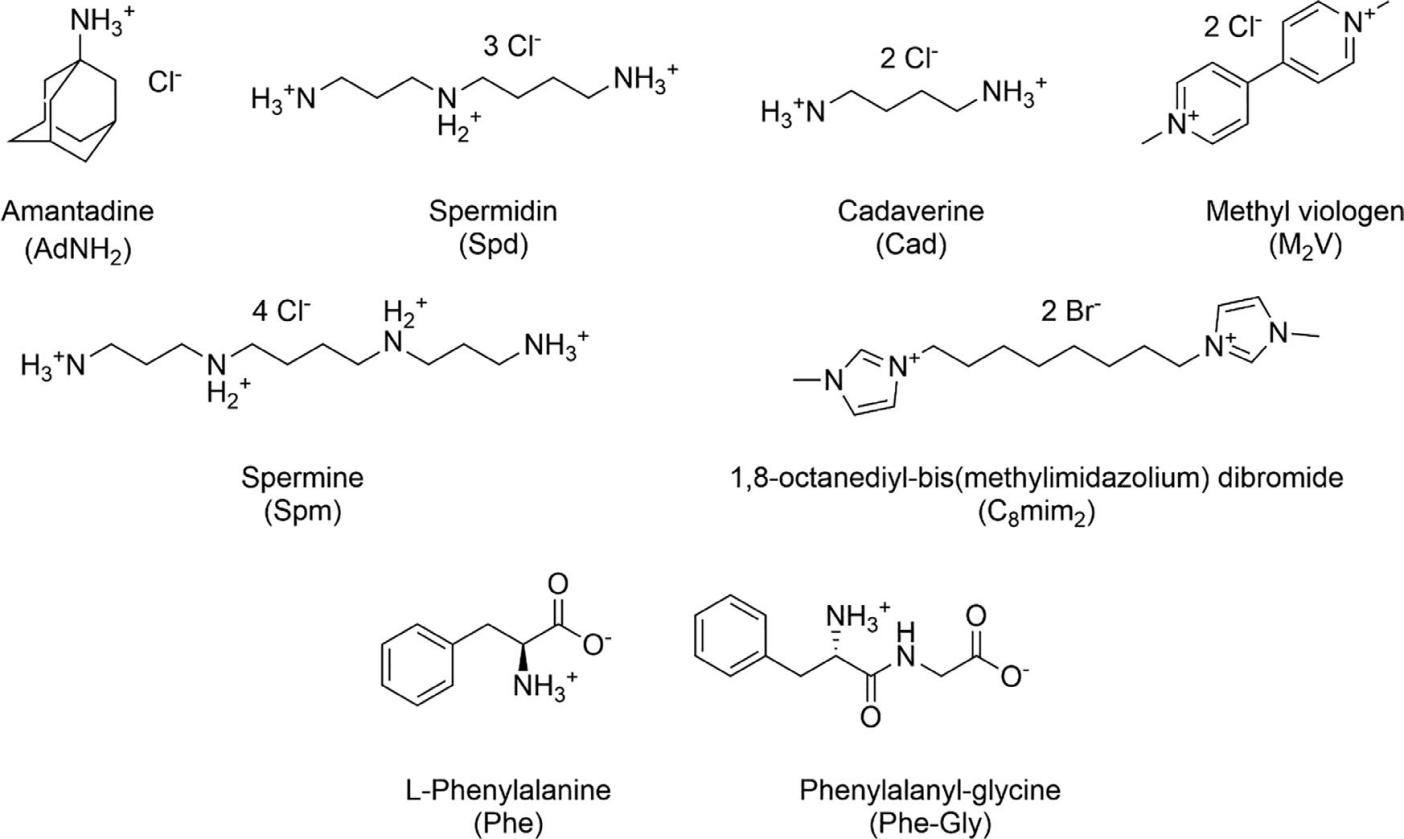
Chemical structures of the small‐molecule guests used to evaluate the reprogrammed host–guest recognition properties of CB7‐Rubpy. The guest panel includes classical cationic CB7 binders—amantadine (AdNH_2_), methyl viologen (M_2_V), cadaverine (Cad), spermine (Spm), and spermidine (Spd)—the aromatic amino‐acid‐derived guests phenylalanine (Phe) and phenylalanyl–glycine (Phe–Gly), and the larger dicationic guest C_8_(mim)_2_^2^^+^. The latter served as a structurally demanding reference guest, particularly for NMR and ion mobility spectrometry studies. Together, these guests were used to assess binding trends by emission spectroscopy, NMR spectroscopy, ion mobility spectrometry, and computational modelling.

^1^H NMR spectroscopy revealed that guest binding induces distinct but architecture‐preserving structural responses. Upon addition of AdNH_2_, a high‐affinity CB7 guest [24, 25], the resonances of the Rubpy unit, including the CH_3_‐bpy probe, remained largely unchanged (Figures 1b and S36), indicating that guest binding occurs while preserving the folded, portal‐associated architecture. In contrast, the sterically more demanding dicationic guest C_8_(mim)_2_^2+^ induced pronounced downfield shifts of both methyl and aromatic bipyridine resonances (Figures 1a and S37), consistent with substantial reorganization of the Ru chromophore relative to the CB7 portal, although without complete unfolding.

IMS and computational analysis supported these distinct binding modes. For [CB7‐Rubpy•C_8_(mim)_2_Br_2_]^2+^, the experimental CCS value of 493.5 Å^2^ agrees with a low‐energy inclusion complex, whereas exclusion structures are energetically disfavored (Figures S38a and S39). For the amantadine complex, calculations favor classical inclusion in solution and an exclusion structure in the gas phase (Figures S38b and S40). In both cases, guest binding is compatible with retention of the folded CB7‐Rubpy architecture. Thus, the proximal Ru chromophore remains part of the recognition environment during guest binding rather than being displaced by complete unfolding.

Emission titrations further support this conclusion. All guests except M_2_V, a known photoinduced electron transfer (PET) quencher [55] of [Ru(bpy)_3_]^2+^, produced pronounced increases in emission intensity (Figures S41–S52). Notably, even in the presence of excess AdNH_2_, the emission spectra did not converge to those of free [Ru(bpy)_3_]^2+^ or to the high‐salt‐induced open state of CB7‐Rubpy (Figure S53), indicating that the folded architecture remains largely intact during guest binding. Control experiments under degassed conditions further excluded displacement of cavity‐bound oxygen as the origin of the observed signal changes (Figure S54).

Emission‐based binding studies revealed that portal‐directed chromophore integration rewrites the selectivity profile of CB7 (Table 1). Classical cationic CB7 guests bind one to two orders of magnitude more weakly than to parent CB7, consistent with electrostatic repulsion from the proximal Ru(II) center and partial portal occlusion. In striking contrast, Phe–Gly binds more strongly to CB7‐Rubpy, with *K_a_* = (5.1 ± 1.2) × 10^7^ M^−^^1^ in water, exceeding parent CB7 (3.3 ± 0.1 × 10^7^ M^−^^1^), and showing a 3.3‐fold stronger binding in PB. A binding mode in which the phenyl side chain remains included in the CB7 cavity while the peptide backbone and terminal groups remain near the portal is plausible, but the available data do not permit a unique geometry or a specific Ru–guest interaction to be assigned. This selectivity shift is therefore attributed to the portal‐associated recognition environment rather than to a single defined microscopic interaction.

**TABLE 1:** Comparison of binding affinities in deionized water and phosphate buffer (PB, 10 mM, pH 7.4). Association constants for CB7‐Rubpy were determined from emission titrations using a 1:1 binding model ([CB7‐Rubpy] = 1 µM, *λ*_exc_ = 452 nm, *λ*_em_ = 610 nm). Values for native CB7 were taken from the cited literature unless indicated otherwise.

		log *K*_a_ (CB7)		log *K*_a_ (CB7‐Rubpy)	
Analyte	Charge	DI water	PB	DI water	PB
M_2_V	2^+^	8.78 [56]	6.82 [57]	6.31 ± 0.09	6.58 ± 0.14
Cad	2^+^	7.41 [58]	6.65 [59]^a^	5.92 ± 0.03	5.35 ± 0.11
Spm	4^+^	7.41 [58]	6.07 [60]^b^	6.10 ± 0.04	5.80 ± 0.04
Spd	3^+^	5.31 [61]	n.a.	5.29 ± 0.03	5.15 ± 0.01
Phe	Zwitterionic	6.01 [62]	5.06 [42]	5.55 ± 0.07	5.08 ± 0.03
Phe‐Gly	Zwitterionic	7.51 ± 0.01^c^	7.08 ± 0.05^c^	7.71 ± 0.10	7.60 ± 0.30
Insulin	Varies	n.a.	6.18 [45]	n.a.	7.19 ± 0.28

^a^ 10 mM ammonium phosphate.

^b^ 20 mM sodium phosphate buffer.

^c^ Determined by an indicator displacement assay under matched medium conditions (Figures S64 and S65).

Collectively, these results demonstrate that covalent integration of the Ru chromophore reprograms CB7 recognition without modifying the host cavity itself. The proximal chromophore creates a portal‐defined recognition environment that weakens binding of classical polycationic guests while favoring aromatic motifs. This rewritten selectivity provides the molecular basis for the motif‐selective protein sensing described below.

### 2.6 Motif‐Selective Protein Sensing With CB7‐Rubpy

Building on the reprogrammed selectivity of CB7‐Rubpy toward aromatic recognition motifs, we next investigated whether this altered recognition behavior can be translated from small‐molecule guests to proteins. Human insulin provides a stringent test of this hypothesis because its B‐chain N‐terminus (PheB1) constitutes a known CB7‐binding motif that combines an accessible aromatic side chain with a terminal ammonium group [41, 45]. To evaluate whether this recognition principle extends to larger proteins, we investigated a panel of biologically relevant proteins and enzymes spanning a broad range of molecular weights, folds, surface charges, and aromatic‐residue presentations, including insulin, RNase A (bovine pancreas), lysozyme (from chicken egg white), lipases (*Pseudomonas cepacia* and *Candida rugosa*), hyaluronidase (bovine testes), horseradish peroxidase, human serum albumin (HSA), and β‐galactosidase (*Aspergillus oryzae*). Specifically, the panel includes compact, predominantly basic proteins (RNase A and lysozyme), the large acidic carrier protein HSA, glycosylated enzymes, and lipases with hydrophobic substrate‐binding surfaces; it therefore spans substantial differences in surface charge, glycosylation, hydrophobicity, and steric accessibility.

Structure‐based inspection of representative protein structures identified potentially exposed phenylalanine residues in several proteins, including F49, F374, and F395 in HSA [63, 64] F47, F81, F108 and F125 in bovine HYAL1 used as a representative sequence/model reference [65, 66] and F119, F122, F142, F146, F221, and F249 in the open conformation of *P. cepacia* lipase [67], whereas lysozyme contains only one clearly exposed phenylalanine candidate (F34) [68] and RNase A contains no clearly surface‐accessible phenylalanine by this criterion [69]. Importantly, solvent exposure of an aromatic ring does not necessarily imply CB7‐Rubpy binding, because productive recognition requires an accessible approach trajectory, compatible sterics and electrostatics at the CB7 portal, and a binding geometry that allows the protein epitope to engage the receptor.

Emission measurements revealed pronounced protein‐dependent responses. In PB (10 mM, pH 7.4), CB7‐Rubpy clearly discriminated between proteins (Figure S55), whereas responses were attenuated in PBS (Figure S56), consistent with the partial population of the open state under high‐salt conditions. Strikingly, insulin produced by far the strongest response of all proteins tested in both buffer systems, emerging as the preferred protein target and demonstrating that the reprogrammed aromatic motif selectivity of CB7‐Rubpy is retained at the protein level. In PB, insulin binding induced a pronounced concentration‐dependent increase in emission intensity (Figure 6a), yielding a high affinity of *K_a, PB_* = (1.53 ± 0.47) × 10^7^ M^−1^ (Figure 6b). In PBS, binding remained strong with *K_a, PBS_* = (1.30 ± 0.42) × 10^6^ M^−1^ (Figure S57b). Notably, the affinity observed for insulin closely mirrors that of Phe–Gly, providing direct evidence that the motif preference established at the small‐molecule level is retained upon transition to protein targets. This behavior is consistent with recognition of the accessible N‐terminal PheB1 motif of insulin. Formation of the CB7‐Rubpy•insulin complex is further supported by ESI‐MS, which shows the intact host–guest assembly as the dominant signal (Figure 6c). The strongest site‐specific assignment in the present study is therefore recognition of N‐terminal phenylalanine motifs, as represented by Phe–Gly and the accessible PheB1 residue of insulin.

**FIGURE 6:**
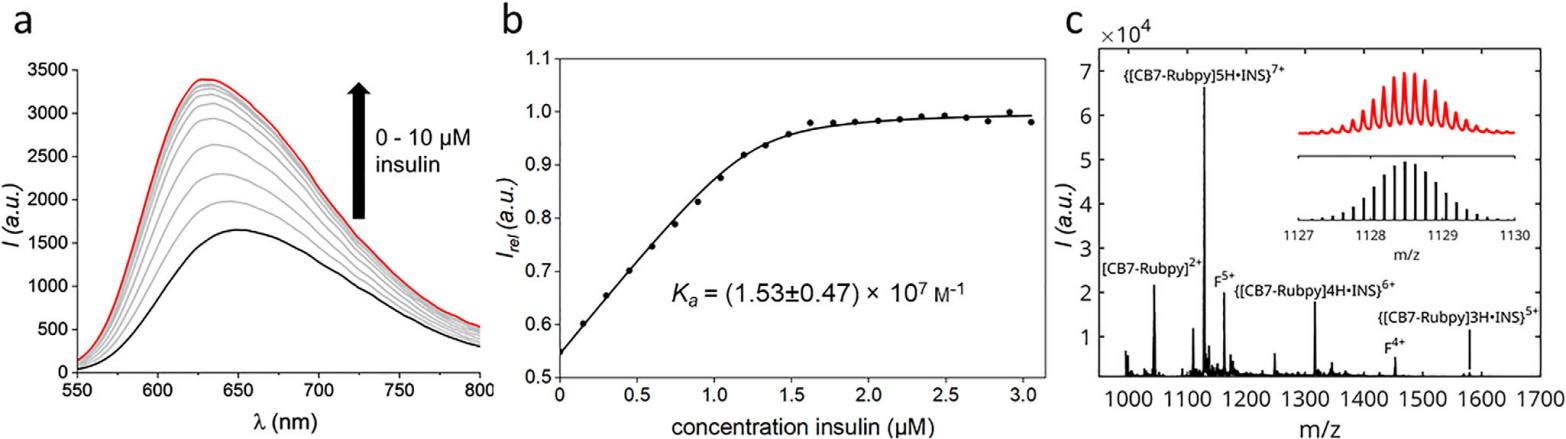
Binding Interaction of CB7‐Rubpy with insulin. (a) Emission spectra (*λ*_exc_ = 452 nm) of CB7‐Rubpy (5 µM) recorded in phosphate buffer (PB, 10 mM, pH 7.4) upon titration with insulin (0–10 µM), showing a concentration‐dependent increase in emission intensity. (b) Binding isotherm obtained from emission titration experiments (CB7‐Rubpy, 1 µM, *λ*_exc_ = 452 nm, *λ*_em_ = 610 nm) with insulin in PB (10 mM, pH 7.4), yielding a binding constant from three replicate measurements of *K*_a_ = (1.53 ± 0.47) × 10^7^ M^−1^. (c) Electrospray ionization mass spectrometry (ESI‐MS) of the CB7‐Rubpy‐insulin complex, while “INS” refers to insulin. The dominant signal corresponds to the complex {[CB7‐Rubpy]5H•INS}^7+^, with additional charge states observed as {[CB7‐Rubpy]4H•INS}^6+^ and {[CB7‐Rubpy]3H•INS}^5+^. Peaks labeled “F” arise from fragment ions and were not analyzed further. The inset shows the measured (top) and calculated (bottom) isotopic distribution of {[CB7‐Rubpy]5H•INS}^7+^.

Unlike classical CB7‐based indicator displacement assays (IDAs), CB7‐Rubpy operates as a unimolecular reporter in which analyte binding directly modulates the chromophore environment without dye displacement. Because recognition and signal generation are integrated within the same molecular construct, binding is translated directly into optical output without the equilibrium penalty associated with indicator displacement. In PB, reliable intensity‐based discrimination between free and insulin‐bound CB7‐Rubpy is maintained down to a sensor concentration of 0.25 µM (Figure S58). At 610 nm, the signal increases by approximately 26% at 0.25 µM; at 0.1 µM, the intensity difference is limited, although a small spectral shift remains visible. In PBS, only minor signal differences are observed at 1 and 0.5 µM, and the spectra largely overlap at lower concentrations (Figure S59). Notably, the previously reported CB7•acridine orange system, studied in PB (10 mM, pH 7.0), exhibits < 5% signal change at 0.5 µM and requires concentrations ≥ 1 µM for reliable discrimination [45].

Among the remaining proteins, bovine testicular hyaluronidase produced the second‐largest response in PB. This is consistent with the presence of several phenylalanine residues in bovine HYAL1 that may serve as accessible aromatic recognition motifs, although the exact binding epitope cannot be assigned without mutagenesis or peptide‐mapping experiments [65]. β‐Galactosidase from *A. oryzae*, HSA, and lipase (*C. rugosa*) showed moderate signal enhancements, indicating partial accessibility of suitable aromatic and/or cationic surface motifs. In contrast, horseradish peroxidase, lipase (*Pseudomonas*/*Burkholderia cepacia*), lysozyme, and RNase A elicited only minor responses. The weak response of horseradish peroxidase suggests that cofactor content and glycosylation alone do not promote CB7‐Rubpy recognition if suitable CB7‐compatible motifs are not presented in an accessible geometry [70]. Similarly, the moderate response of HSA, despite the presence of several solvent‐exposed internal phenylalanine residues, shows that CB7‐Rubpy does not simply report on the total number of aromatic residues [63]. Unlike Phe–Gly and insulin PheB1, these internal phenylalanines are embedded in the protein surface and lack the same terminal ammonium/aromatic recognition motif. The weak responses of lysozyme and RNase A are consistent with limited availability of CB7‐compatible phenylalanine motifs, whereas the weak response of *Pseudomonas cepacia* lipase, despite exposed phenylalanines in the open crystal structure, indicates that solution conformation and steric approachability are also critical. In PBS, only insulin and hyaluronidase retained measurable signal enhancement. Thus, the sensing response does not correlate with protein size, molecular weight, cofactor content, glycosylation, or aromatic‐residue abundance, but with the geometric and electrostatic accessibility of CB7‐compatible recognition motifs.

### 2.7 Monitoring Denaturation‐Derived Protein States

Having shown that CB7‐Rubpy recognizes accessible aromatic protein motifs, we next tested whether this reprogrammed recognition could report on motif accessibility during protein denaturation and refolding. Insulin, HSA, lysozyme, and RNase A were subjected to thermal and chemical denaturation–recovery protocols in PB, and CB7‐Rubpy emission was measured after refolding (Figure 7a). Thus, the response reports on the recovered protein states rather than transient unfolded conformations.

**FIGURE 7:**
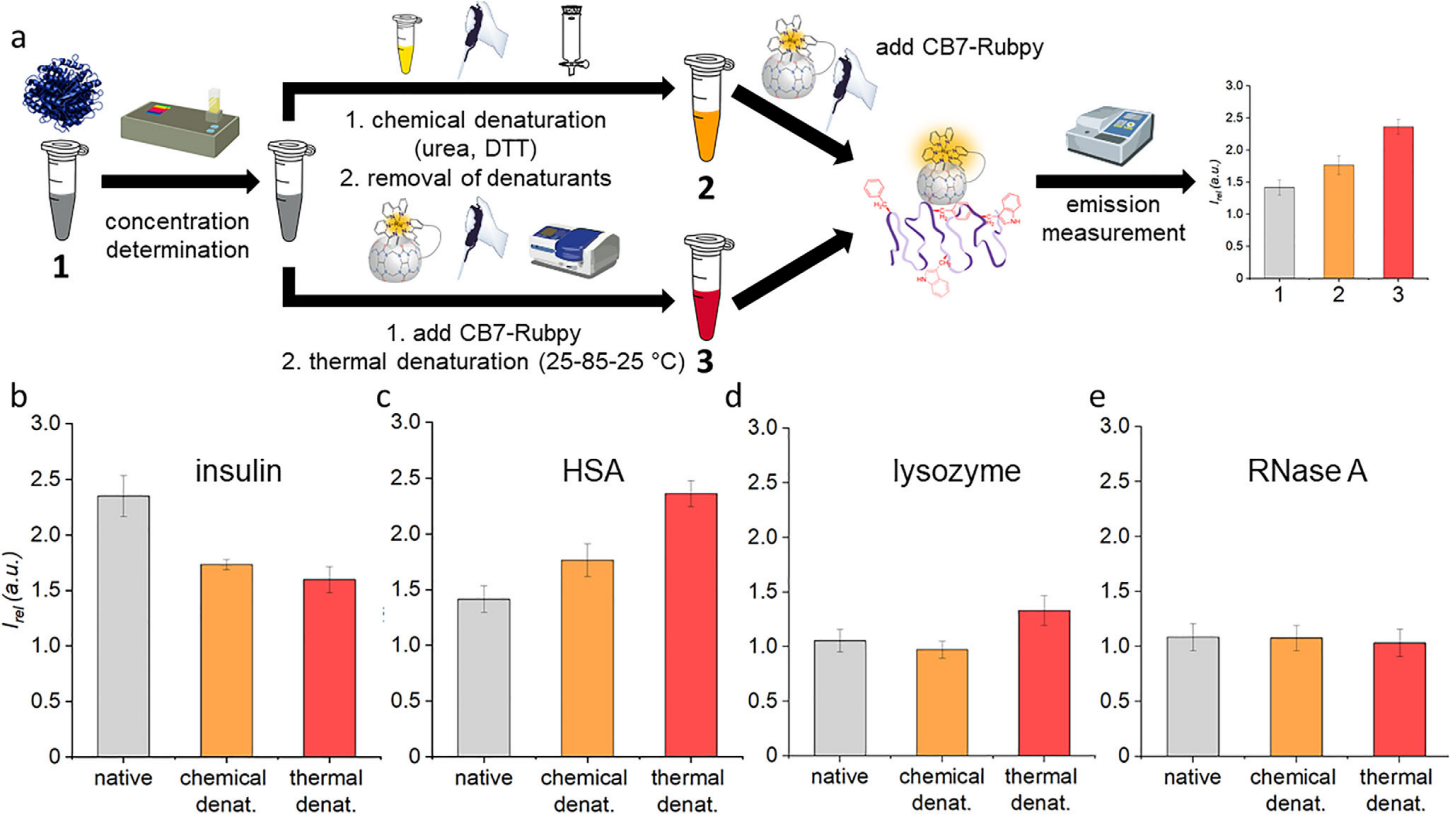
Influence of protein denaturation on the photoluminescent response of CB7‐Rubpy. (a) Schematic illustration of the experimental workflows for chemical and thermal denaturation. Protein concentrations were first determined by UV absorption. For chemical denaturation, proteins were treated with urea and DTT, followed by removal of denaturants via size‐exclusion chromatography prior to addition of CB7‐Rubpy and emission measurement. For thermal denaturation, proteins were mixed with CB7‐Rubpy and subjected to a heating–cooling cycle (25°C→85°C→25°C) before measurement. Both treatments disrupt protein secondary structure. (b–e) Bar plots showing the emission intensities (*λ*_exc_ = 452 nm, *λ*_em_ = 610 nm) of CB7‐Rubpy (5 µM) in the presence of native, chemically denatured (urea 6 M, DTT 2 mM), and thermally denatured proteins (each 5 µM) in phosphate buffer (PB, 10 mM, pH 7.4) for (b) insulin, (c) HSA, (d) lysozyme, and (e) RNase A. Emission intensities are normalized to the corresponding blank measurements.

Distinct protein‐dependent responses were observed after recovery (Figure 7b–e). For insulin, emission decreased by approximately 30%, indicating that the native binding‐competent motif is not fully restored, consistent with altered presentation of the N‐terminal PheB1 residue. In contrast, HSA showed enhanced emission after recovery, increasing by ∼20% after chemical denaturation and ∼40% after thermal denaturation. Because mature HSA lacks a free N‐terminal phenylalanine motif but contains several solvent‐exposed internal phenylalanine residues, including F49, F374, and F395, this response is most consistently explained by partially refolded states that alter the accessibility or local environment of internal aromatic residues and/or nearby cationic surface patches. Lysozyme and RNase A showed only negligible changes. This is consistent with the limited availability of CB7‐compatible phenylalanine motifs in their native structures and suggests that denaturation–recovery does not generate additional accessible recognition motifs that are productively engaged by CB7‐Rubpy. Thus, CB7‐Rubpy responds not only to protein identity, but also to denaturation‐induced changes in the presentation of CB7‐compatible aromatic motifs.

Circular dichroism (CD) spectroscopy after the same recovery protocols provided a complementary global readout of refolding. After chemical denaturation (Figure S60), HSA, which gave the strongest CB7‐Rubpy response, showed clear CD signatures of incomplete refolding, whereas RNase A showed minimal changes in both CD and CB7‐Rubpy emission. CD measurements after thermal denaturation confirmed the same trend (Figure S61): HSA exhibited the largest spectral changes, insulin showed altered signatures after recovery, and lysozyme and RNase A displayed only minor changes, consistent with more efficient recovery of their native secondary structure. Thus, CD reports on global secondary‐structure recovery, while CB7‐Rubpy reports on local recognition‐motif accessibility. Together, these data show that CB7‐Rubpy detects denaturation‐derived states through changes in recognition‐motif presentation rather than through global protein folding alone.

The reprogrammed recognition properties of CB7‐Rubpy therefore extend beyond protein identification to conformational‐state sensing. By reporting on the accessibility of CB7‐compatible aromatic recognition motifs rather than on global protein structure, CB7‐Rubpy provides information complementary to conventional spectroscopic methods and enables detection of partially refolded or non‐native protein states. More broadly, these results demonstrate that reprogrammed supramolecular recognition can be used not only to identify biomolecular targets but also to monitor changes in their structural presentation, extending the concept of motif‐selective recognition from molecular sensing to conformational‐state analysis.

## 3 Conclusion

In summary, we introduce CB7‐Rubpy as a portal‐preorganized host–chromophore conjugate that integrates recognition, selectivity tuning, and signal generation within a single macrocyclic receptor. Covalent positioning of the Ru chromophore at the CB7 portal creates a secondary recognition interface while preserving the native binding cavity. This portal‐defined environment reprograms CB7 selectivity, weakening binding of classical polycationic guests while favoring CB7‐compatible aromatic peptide and protein motifs, as demonstrated by the enhanced affinities for Phe–Gly and insulin. At the protein level, this rewritten selectivity enables motif‐selective sensing of accessible aromatic epitopes, particularly N‐terminal phenylalanine motifs, and translates binding directly into emission without indicator displacement. The same recognition principle further allows CB7‐Rubpy to report denaturation‐derived protein states through changes in local aromatic motif presentation. Control experiments with free [Ru(bpy)_3_]^2^^+^ (Figure S62) and the noncovalent Ada‐Ru•CB7 system (Figure S63) support that this analyte–responsive behavior requires covalent portal preorganization, an accessible CB7 recognition site, and an integrated chromophore. Thus, portal‐preorganized chromophore integration converts CB7 from an optically silent, charge‐selective host into an emissive motif‐selective receptor and provides a design strategy for coupling selectivity tuning and signal transduction in CB‐based receptors.

## Author Contributions

**Patrick Gruhs**: conceptualization, investigation, methodology, validation, writing – original draft, writing – review and editing, visualization. **Albert Kraft**: writing – original draft, writing – review and editing, investigation, data curation, methodology, visualization. **Vahideh Mahram**: investigation, writing – review and editing. **Marco Neumaier**: methodology, conceptualization, investigation, visualization, writing – original draft. **Papri Chakraborty**: investigation, methodology. **Emily Janz**: investigation, validation. **Maria Vittoria Balli**: investigation. **Pierre Picchetti**: supervision. **Luca Prodi**: supervision, resources, writing – review and editing, funding acquisition. **Manfred Kappes**: writing – review and editing, supervision, resources, funding acquisition. **Frank Biedermann**: conceptualization, funding acquisition, writing – original draft, supervision, resources, project administration.

## Conflicts of Interest

The authors declare no conflicts of interest.

## Supporting information




**Supporting File**: anie74610‐sup‐0001‐SuppMat.pdf.

## Data Availability

The data supporting the main findings of this study are provided in the Supporting Information and have been deposited in the Chemotion Repository and the Zenodo database. Further information is available from the corresponding authors upon reasonable request. Additional references are cited in the Supporting Information.
