# Supplementary material for: A Portal‐Preorganized Cucurbit[7]uril‐Ruthenium Conjugate Enables Motif‐Selective Protein Sensing
Source: Angew Chem Int Ed Engl. 2026 Sep 8;65(44):e6821024. doi: 10.1002/anie.6821024 (PMC13703776; doi:10.1002/anie.6821024)
Supplement: Supplementary file 1 [file ANIE-65-e6821024-s001.pdf]

Supporting Information

A Portal-Preorganized Cucurbit[7]uril-Ruthenium Conjugate Enables Motif-Selective Protein Sensing

Patrick Gruhs[a], Albert Kraft[a], Vahideh Mahram[a], Marco Neumaier[a], Papri Chakraborty[a], Emily Janz[a], Maria Vittoria Balli[b], Pierre Picchetti[a], Luca Prodi[b], Manfred Kappes*[a], Frank Biedermann*[a]

[a]Institute of Nanotechnology, Karlsruhe Institute of Technology (KIT), Kaiserstrasse 12, 76131 Karlsruhe (Germany)
[b]Department of Chemistry “Giacomo Ciamician”, Alma Mater Studiorum - University of Bologna, via Gobetti 85, 40129 Bologna (Italy)

*Manfred Kappes — E-mail: manfred.kappes@kit.edu
*Frank Biedermann — E-mail: frank.biedermann@kit.edu

Table of Contents

1. List of abbreviations **3**

2. Materials and instrumentation **4**

2.1 Chemicals *4*

2.2 General instrumentations *4*

2.2.1 Absorption and emission spectroscopy 4
2.2.2 Nuclear magnetic resonance (NMR) spectroscopy 5
2.2.3 High performance liquid chromatography (HPLC) 5
2.2.4 Electrospray ionization mass spectrometry (ESI-MS) 5
2.2.5 Ion mobility spectrometry experiments 5
2.2.6 Circular dichroism spectroscopy 5

3. Experimental **6**

3.1 Synthetic remarks *6*

3.1.1 Synthetic step a 7
3.1.2 Synthetic step b 9
3.1.3 Synthetic step c 13
3.1.4 Ada-Ru 15

3.2 Experimental details *18*

3.2.1 Emission-based detection 18
3.2.2 Quantum chemical methods and calculation of collision cross sections. 18
3.2.3 Calculation of the degree of complexation 18
3.2.4 Structure-guided assessment of phenylalanine accessibility 19
3.2.5 Chemical denaturation of proteins 20
3.2.6 Emission titrations with salt solutions 21

4. Supporting Tables **21**

5. Supporting Figures **22**

6. Supporting References **50**

1. List of abbreviations

ACN: Acetonitrile
Ada-Ru: Adamantane-functionalized Ru(bpy) complex / reference compound
aISS: Automated interaction site screening; docking submodel used for host-guest complexes
BiPy-COOH: 4-(4′-methyl-[2,2′-bipyridin]-4-yl)butanoic acid
Bpy / bpy: 2,2′-Bipyridine / bipyridyl ligand
$C_8(mim)_2Br_2$: Bis-imidazolium bromide guest/additive used for CB7-containing ESI-MS samples
CB7: Cucurbit[7]uril
CB7-OH: Mono-hydroxylated cucurbit[7]uril
CB7-OPr: Mono-propargyloxylated cucurbit[7]uril
CB7-Rubpy: Cucurbit[7]uril-Ru(bpy) conjugate used as the sensor
CD: Circular dichroism
CREST: Conformer-Rotamer Ensemble Sampling Tool
CuAAC: Copper-catalyzed azide-alkyne cycloaddition
DFT: Density functional theory
DI water: Deionized water
DTT: Dithiothreitol
ESI-MS: Electrospray ionization mass spectrometry
GFN2-xTB: Geometry, frequency and noncovalent extended tight-binding method
HG: Host-guest complex
HPLC: High performance liquid chromatography
HPLC-MS: High performance liquid chromatography-mass spectrometry
HSA: Human serum albumin
IMS-MS: Ion mobility spectrometry-mass spectrometry
LC: Liquid chromatography
M_2V: Methyl viologen
MCI GEL CHP20P: Polymeric adsorbent used for final purification
MLCT: Metal-to-ligand charge transfer
MS: Mass spectrometry
NMR: Nuclear magnetic resonance
PB: Phosphate buffer
PBS: Phosphate-buffered saline
Phe: Phenylalanine
Phe-Gly: Phenylalanyl-glycine
PMMA: Poly(methyl methacrylate)
r.t.: Room temperature
$Ru(bpy)_3$: Tris(2,2′-bipyridyl)ruthenium(II)
Ru-COOH: Carboxyl-functionalized ruthenium complex
$Ru-N_3$: Azide-functionalized ruthenium complex
Ru-NHS: NHS-activated ruthenium complex
RuBiPy: Ruthenium bipyridine reference compound
SEC: Size-exclusion chromatography
Spm: Spermine
TEG: Triethylene glycol linker unit
THF: Tetrahydrofuran
THPTA: Tris(3-hydroxypropyltriazolylmethyl)amine
timsTOF: Trapped ion mobility spectrometry-time-of-flight mass spectrometer
UFF: Universal force field
UV-Vis: Ultraviolet-visible
v/v: Volume per volume

2. Materials and instrumentation

2.1 Chemicals

Ultrapure (Milli-Q) water was used for all experiments. PB buffer was prepared by dissolving 2.021 g Sodium Phosphate Dibasic Heptahydrate and 0.3394 g Sodium Phosphate Monobasic Monohydrate in 1 L of Milli-Q water. pH adjustment was done with diluted NaOH or HCl solutions. PBS buffer was prepared by dissolving 8 g Sodium chloride, 0.2 g Potassium Chloride, 1.44 g Sodium Phosphate Dibasic and 0.245 g Potassium Phosphate Monobasic in 1 L of Milli-Q water. pH adjustment was done with diluted NaOH or HCl solutions. Sodium phosphate monobasic monohydrate, sodium phosphate dibasic heptahydrate, silver hexafluorophosphate, copper sulfate anhydrous, L-ascorbate, amantadine hydrochloride, methyl viologen dichloride, human recombinant insulin, β-galactosidase from Aspergillus oryzae, horseradish peroxidase, human serum albumin fatty acid free, hyaluronidase from bovine testes, lipase from Pseudomonas cepacia, lipase from Candida rugosa, lysozyme from chicken egg white, RNase A, and *cis*-bis(2,2′-bipyridine)dichlororuthenium(II) were purchased from Sigma-Aldrich. Tris(2,2′-bipyridyl)ruthenium(II) chloride hexahydrate $[Ru(bpy)_3]Cl_2 \cdot 6H_2O$ was purchased from Merck Life Science S.r.l. 4-(4'-methyl-[2,2'-bipyridin]-4-yl)butanoic acid, 11-azido-3,6,9-trioxaundecan-1-amine and tris(3-hydroxypropyltriazolylmethyl)amine (THPTA) were purchased from BLDpharm. Nandrolone (or 19-nortestosterone) was purchased from Merck Life Science s.r.l. *N*-Hydroxysuccinimide (NHS) was purchased from Acros Organics.

Cucurbit[7]uril (CB7),[1] mono-hydroxylated cucurbit[7]uril (CB7-OH)[2] and mono-propargyloxylated cucurbit[7]uril (CB7-OPr)[3] were prepared according to procedures reported in literature.

For the final purification of CB7-Rubpy, MCI GEL® CHP20P Polymeric Adsorbent was used. Column packing and regeneration was performed as instructed by the manufacturer.

2.2 General instrumentations

2.2.1 Absorption and emission spectroscopy

Absorbance Spectra. Absorbance spectra were measured at 25°C in Milli-Q water, PBS or PB buffer on a JASCO V-730 double-beam UV-Vis spectrophotometer. For UV-Vis absorption experiments, PMMA cuvettes with a light path of 10 mm and dimensions of 10 × 10 mm from Brand with a spectroscopic cut-off at 220 nm were utilized. The samples were equilibrated by using a water thermostatic cell holder STR-812, while the cuvettes were equipped with a stirrer allowing rapid mixing.

Emission spectra. Emission spectra were recorded on a JASCO FP-8300 fluorescence spectrometer equipped with a 450 W Xenon arc lamp and double-grating excitation and emission monochromators. All measurements were performed at 25 °C using a thermostated cell holder (STR-812), and samples were continuously stirred to ensure rapid mixing.

For fluorescence-based titration experiments, PMMA cuvettes (Brand) with a 10 mm light path and dimensions of 10 × 10 mm (spectroscopic cut-off at 220 nm) were used.

Spectral correction was performed using a Rhodamine B solution in ethanol at high concentration, which exhibits a quantum yield close to unity. Emission spectra of this reference were recorded with a calibrated spectrometer to determine an instrument-specific correction factor in the wavelength range of 520-800 nm. This correction factor was subsequently applied to all measured emission spectra to account for wavelength-dependent variations in detector and optical system response.

2.2.2 Nuclear magnetic resonance (NMR) spectroscopy

Proton (^{1}H)- and carbon (^{13}C)- NMR spectra were recorded in deuterium oxide, acetonitrile-*d*$_3$, chloroform-*d*$_3$ or DMSO-*d*$_6$, depending on the sample, on a Bruker Avance 400 spectrometer at 25°C. The ^{1}H-NMR and ^{13}C-NMR chemical shifts (δ) are given in ppm and refer to residual protons on the corresponding deuterated solvent.

2.2.3 High performance liquid chromatography (HPLC)

Analytical HPLC analyses were performed on an LC-2000Plus HPLC system equipped with a UV-2075 UV-Vis detector and a Kromasil 100 C18 5 µm LC column (250 × 4.6 mm, Agela) at a flow rate of 0.8 mL/min.

2.2.4 Electrospray ionization mass spectrometry (ESI-MS)

ESI-MS spectra were recorded on an Orbitrap Exploris 240 mass spectrometer (Thermo Fisher Scientific) equipped with a HESI II probe in positive-ion mode. The instrument was calibrated over m/z 200-2000 using a premixed FlexMix calibration solution. Samples were analyzed with a spray voltage of 1.75 kV, and the ion-transfer tube temperature was set to 320 °C. CB7-containing samples were dissolved in a 10 mM aqueous solution of $[C_8(mim)_2]Br_2$ and diluted to 1 mL with MeOH, all other samples were prepared by dissolving the compound in MeOH. MeOH:H_2O (1:1 v/v) mixture was used as eluent. Injection of the samples was carried out with the aid of a Hamilton® syringe 725RN 500 µL at a flow rate of 25 µL/min, using a single syringe pump.

2.2.5 Ion mobility spectrometry experiments

IMS-MS measurements were performed on a Bruker timsTOF instrument. The IMS ramp start was set to 0.78, 0.98, and 1.02 V s cm^{-2} for $[CB7\text{-}Rubpy•amantadine]^{3+}$, $[CB7\text{-}Rubpy]^{2+}$, and $[CB7\text{-}Rubpy•C8(mim)_2Br_2]^{2+}$, respectively, with the ramp end adjusted to 0.1 V s cm^{-2} higher in each case. The ramp time was 500 ms and the cycle accumulation time was 10 ms. Tunnel-in and tunnel-out pressures were set to 2.50 and 0.78 mbar, respectively. Electrospray ionization was performed using a capillary voltage of 3–5 kV, an end-plate offset of −2 kV, nebulizer pressure of 0.3 bar, dry gas flow of 3.5 L min^{-1}, and a dry gas temperature of 200 °C.

Samples were prepared from 500 µM aqueous stock solutions, diluted 20-fold with Milli-Q water and subsequently with acetonitrile (water/acetonitrile 1:2, v/v). Approximately 20 µL formic acid were added to 2 mL of the diluted solution prior to analysis, and the samples were introduced into the electrospray ionization source of the Bruker timsTOF mass spectrometer.

Experimental reduced ion mobilities were converted to collision cross sections ($^{tims}CCS_{N2}$) using calibration with Agilent ESI-L Low Concentration Tuning Mix measured under identical instrumental conditions.[4, 5] All measurements were performed at least in duplicate on different days, and average $^{tims}CCS_{N2}$ values were used. Repeated measurements showed deviations below 0.5%, indicating high reproducibility.

Conformational sampling was performed using the Conformer–Rotamer Ensemble Sampling Tool (CREST)[6] in combination with the GFN2-xTB semiempirical method.[7] Representative low-energy structures were subsequently optimized using the r²SCAN-3c density functional method.[8] For host–guest complexes, initial binding poses were generated using aISS docking prior to CREST sampling. Theoretical collision cross sections ($^{TM}CCS_{N2}$) were calculated using trajectory method simulations.

2.2.6 Circular dichroism spectroscopy

CD spectra were recorded on a JASCO J-1500 CD spectrometer equipped with a Jasco PTC-510 temperature controller. The fluorescence spectra were recorded on a JASCO FP-8300 fluorescence

spectrometer equipped with a 450 W Xenon arc lamp and double-grating excitation and emission monochromators. All measurements were performed at 25 °C using a thermostated cell holder (STR-812). Blank measurements of buffer provided no induced CD signals in the regions examined. All CD spectra reported are baseline corrected for the appropriate solvent system used. For spectroscopy analysis in quartz cuvettes, Suprasil (type 111-QS) emission cuvettes with a light path of 10 mm and dimensions of 10 × 10 mm from Hellma-Analytics were utilized. For thermal denaturation studies, a heating rate of 2 °C min⁻¹ was applied from 25 to 85 °C, followed by equilibration at 85 °C for 20 min. The sample was then cooled back to 25 °C.

3. Experimental

3.1 Synthetic remarks

The CB7-Rubpy system was synthesized *via* a copper-catalyzed azide-alkyne cycloaddition (CuAAC). More specifically, *cis*-bis(2,2′-bipyridine)dichlororuthenium(II) was converted into an azide-functionalized complex (Ru-N_3) through a three-step sequence, involving its activation to the NHS ester (synthetic step **a**) and the subsequent amidation with a bifunctional azido-amine linker (synthetic step **b**). Subsequently, mono-propargyloxylated CB7 (CB7-OPr) was prepared from CB7 *via* sequential hydroxylation and *o*-alkylation. Eventually, the final click coupling of Ru-N_3 and CB7-OPr was carried out under standard CuAAC conditions in the presence of THPTA, affording the CB7-Rubpy in an isolated yield of 40% (synthetic step **c**). The entire synthetic route has been summarized in Figure S1.

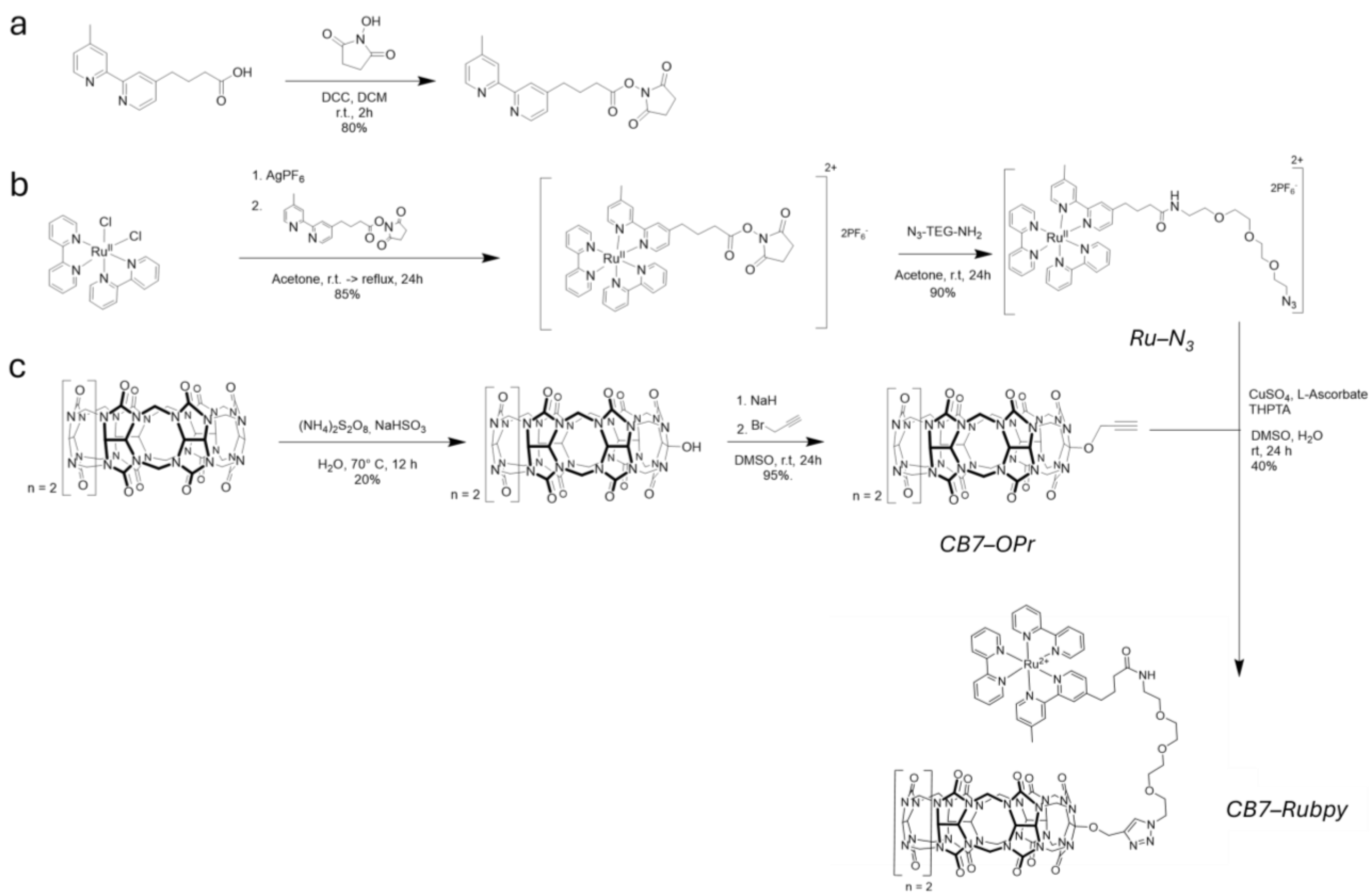

Figure S1. Schematic representation of the synthetic procedure for CB7-Rubpy, reporting the obtained yields for each step.

3.1.1 Synthetic step a

2,5-dioxopyrrolidin-1-yl 4-(4'-methyl-[2,2'-bipyridin]-4-yl)butanoate

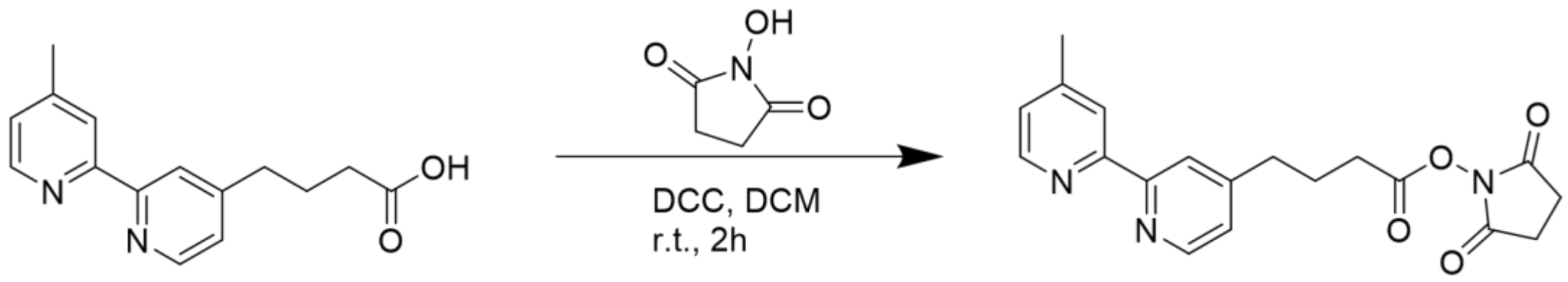

According to literature procedures[9], BiPy-COOH (150 mg, 0.6 mmol, 1 equiv.) was dissolved in a flame dried flask in dry DCM (5 mL) under N_2 atmosphere. DCC (132.8 mg, 0.6 mmol, 1.1 equiv.), and NHS (74.1 mg, 0.6 mmol, 1.1 equiv.) were added and stirred at r.t. for 2 h. The mixture was then filtered and washed with DCM. The solvent was evaporated *in vacuo* and the crude product was purified by column chromatography. (EtOAc/Hex 1:1, then EtOAc/MeOH 95:5). The product was obtained as a semi-solid. (203.3 mg, 0.57 mmol, 98%) ^{1}H-NMR (400 MHz, $CDCl_3$) δ: 8.65-8.51 (m, 2H), 8.26 (d, J = 11.5 Hz, 2H), 7.23-7.10 (m, 2H), 2.90-2.79 (m, 6H), 2.66 (t, J = 7.3 Hz, 2H), 2.45 (s, 3H), 2.23-2.12 (m, 2H). ^{13}C-NMR (101 MHz, $CDCl_3$) δ: 169.06 (s), 149.24 (s), 124.85 (s), 124.12 (s), 122.24 (s), 121.35 (s), 34.08 (s), 30.26 (s), 25.60 (s), 25.19 (s), 21.25 (s). ESI-MS: hydrolyzes

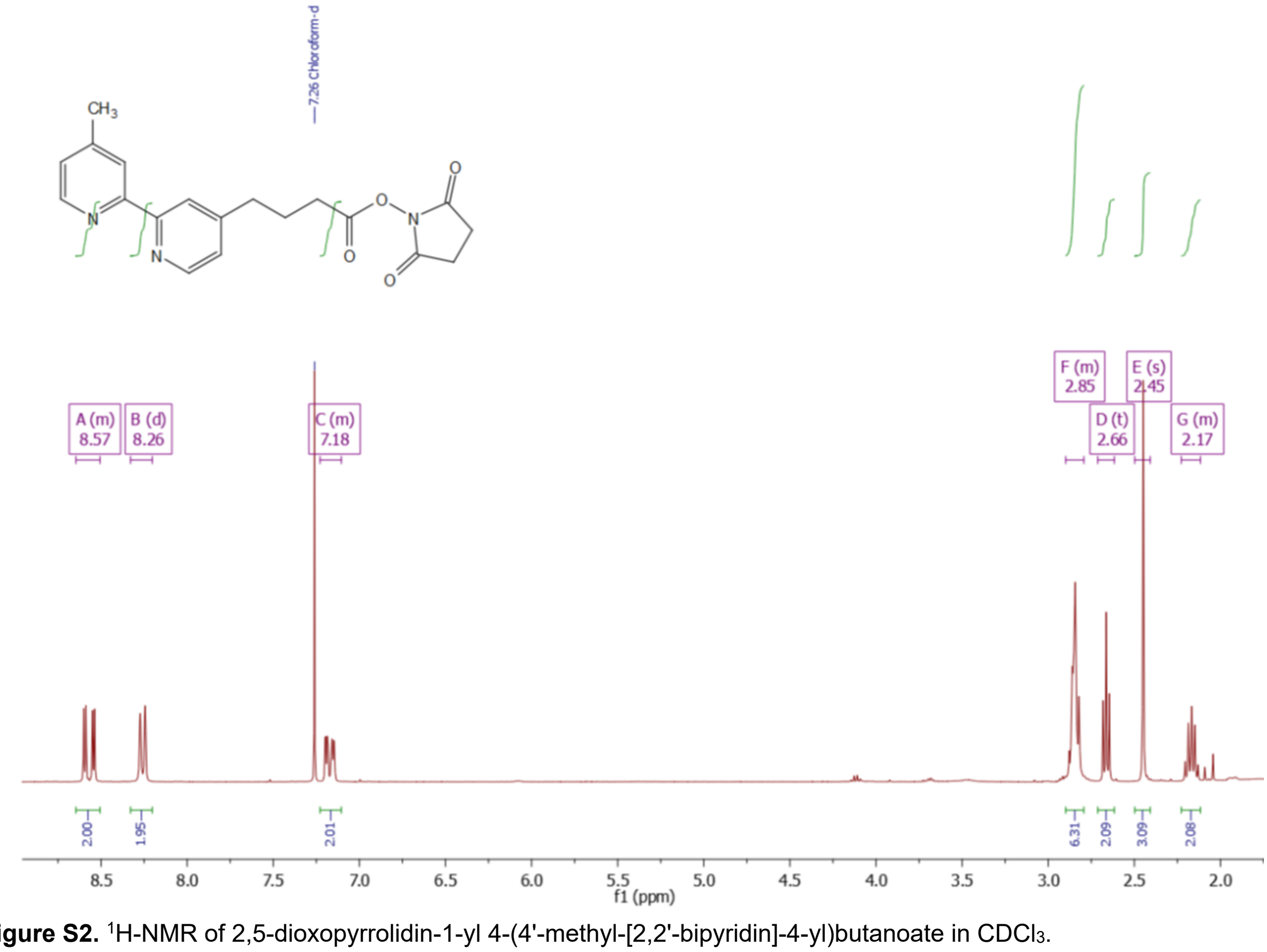

Figure S2. ^{1}H-NMR of 2,5-dioxopyrrolidin-1-yl 4-(4'-methyl-[2,2'-bipyridin]-4-yl)butanoate in $CDCl_3$.

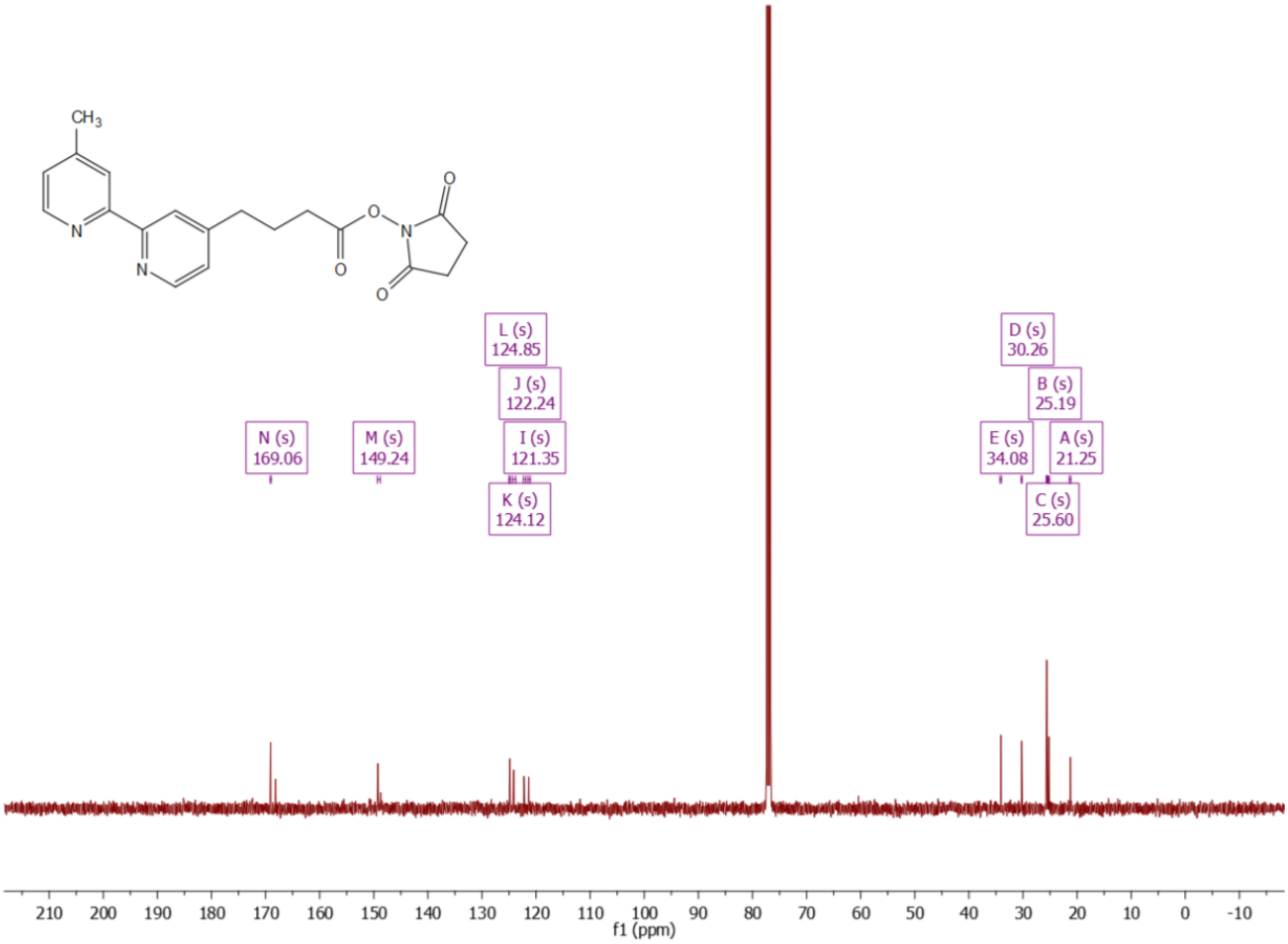

Figure S3. ^{13}C-NMR of 2,5-dioxopyrrolidin-1-yl 4-(4'-methyl-[2,2'-bipyridin]-4-yl)butanoate in $CDCl_3$.

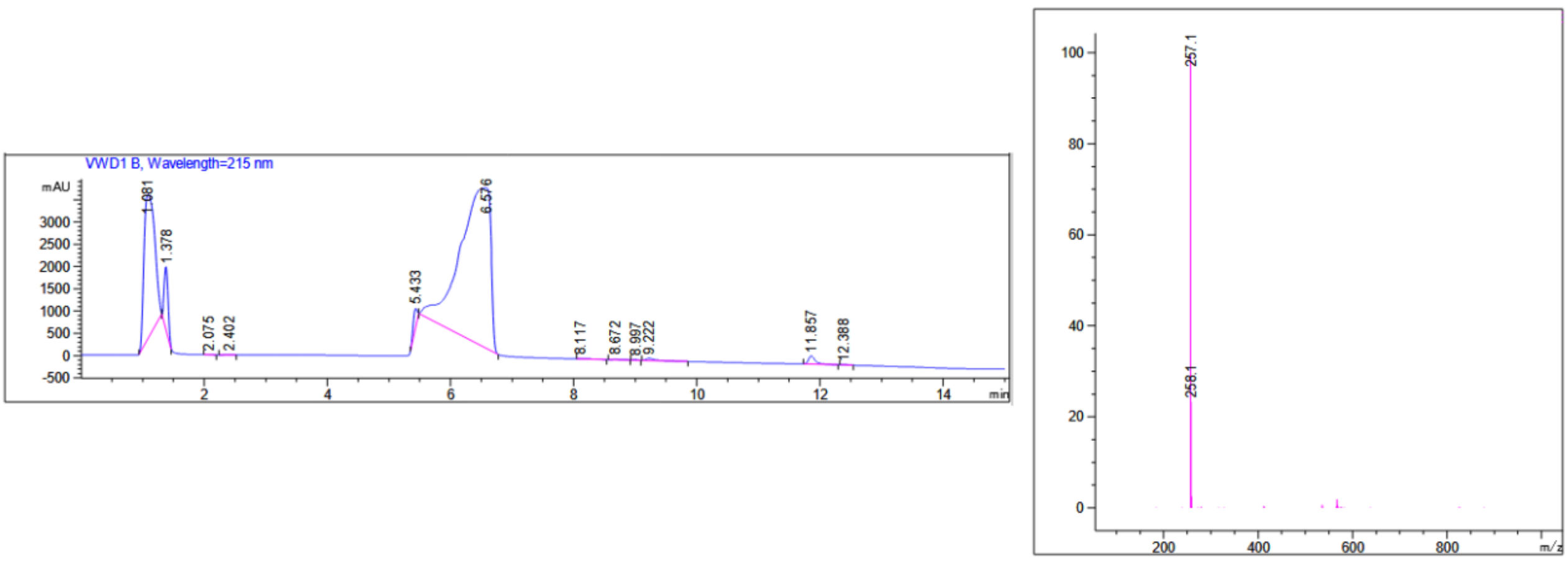

Figure S4. HPLC-MS of 2,5-dioxopyrrolidin-1-yl 4-(4'-methyl-[2,2'-bipyridin]-4-yl)butanoate measured (top) and simulated (bottom).

Additional information can be found in the chemotion repository: https://dx.doi.org/10.14272/reaction/SA-FUHFF-UHFFFADPSC-GWDYGOLOEZ-UHFFFADPSC-NUHFF-NUHFF-NUHFF-ZZZ.1

3.1.2 Synthetic step b

Ru-NHS

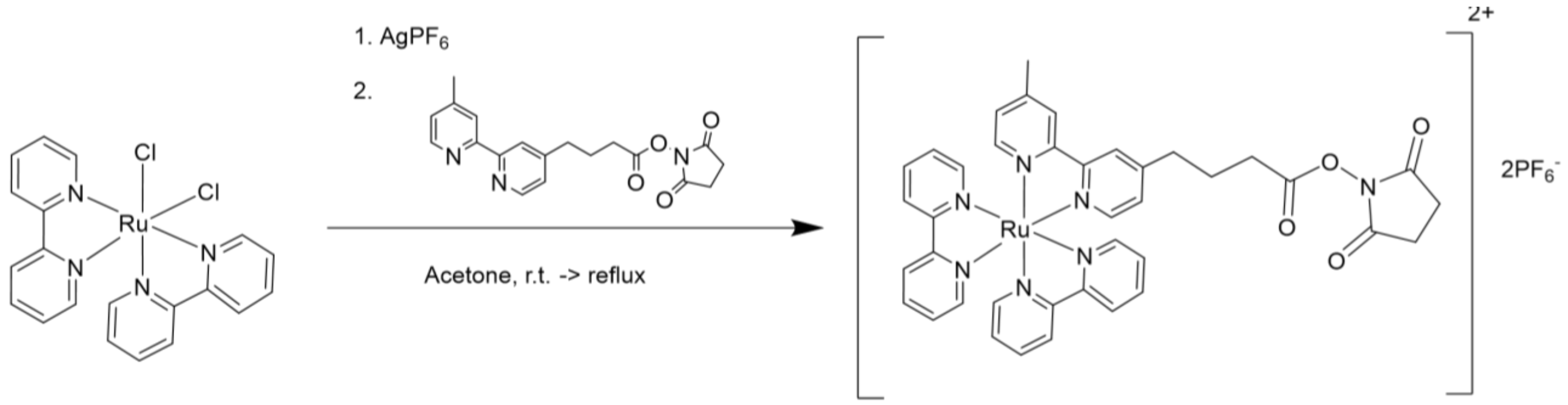

According to literature procedures[10], *cis*-bis-(2,2'-bipyridine)-dichlororuthenium (II) (200 mg, 0.4 mmol, 1 equiv.) and $AgPF_6$ (208.8 mg, 0.82 mmol, 1.5 equiv.) were added to a flame dried Schlenk flask, and the flask was evaporated and backfilled with N_2. Dry acetone (40 mL) was then added and the solution was purged with N_2. The reaction mixture was allowed to stir at room temperature under N_2 atmosphere for 8 h. Bpy ligand (218.9 mg, 0.62 mmol, 1.5 equiv.) was added to the mixture dissolved in dry acetone (2 mL), then stirred for 24 h at reflux under N_2 atmosphere. The solution was filtered twice to remove all AgCl precipitate and the solvent was evaporated *in vacuo*. Et_2O (100 mL) was added, and the initially dark-red sticky solid turned into a light-red solid, which was collected by centrifugation and then dried under a high vacuum. The product was obtained as a red solid (406.6 mg, 0.38 mmol, 93%). ^{1}H-NMR (400 MHz, CD_3CN) δ: 8.50-8.44 (m, 2H), 8.41-8.33 (m, 1H), 8.26 (d, J = 6.8 Hz, 1H), 8.08-8.00 (m, 2H), 7.72 (dd, J = 8.9, 4.9 Hz, 2H), 7.60-7.49 (m, 1H), 7.43-7.34 (m, 2H), 7.25 (dt, J = 9.0, 2.8 Hz, 2H), 2.90 (t, J = 7.6 Hz, 1H), 2.86-2.80 (m, 1H), 2.77 (s, 1H), 2.69 (dt, J = 11.9, 7.3 Hz, 2H), 2.53 (s, 2H). ^{13}C NMR (101 MHz, CD_3CN) δ 157.65 (s), 152.39 - 151.96 (m), 138.17 (s), 128.14 (d, J = 12.7 Hz), 124.81 (d, J = 8.1 Hz), 117.90 (s), 25.73 (s), 1.64 - 0.48 (m), 0.27 (s), 0.22 - -0.96 (m). ESI-MS: calcd. for $[M]^{2+}$ = 383.5897; found = 383.6044.

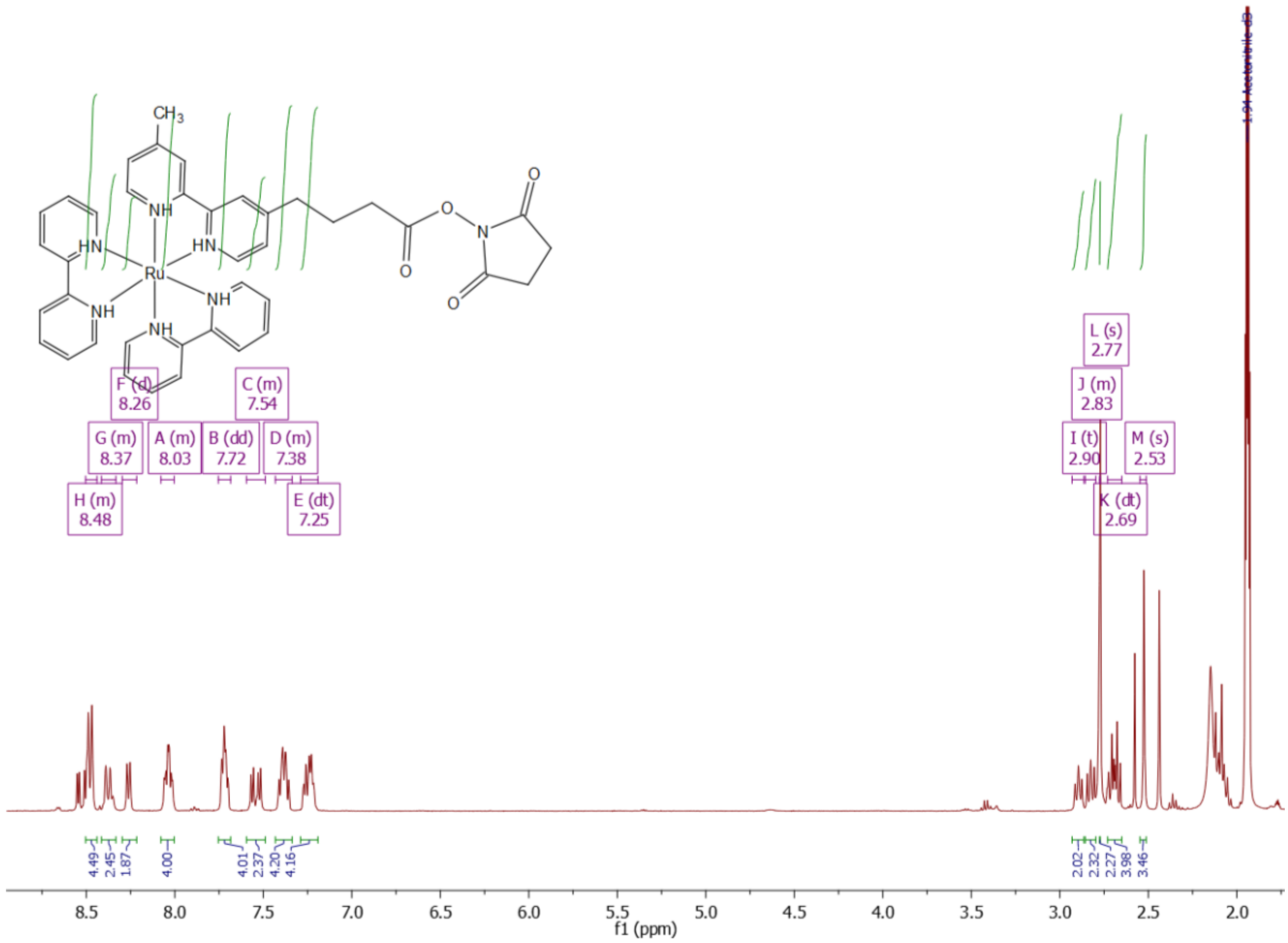

Figure S5. ^{1}H-NMR of Ru-NHS in CD_3CN.

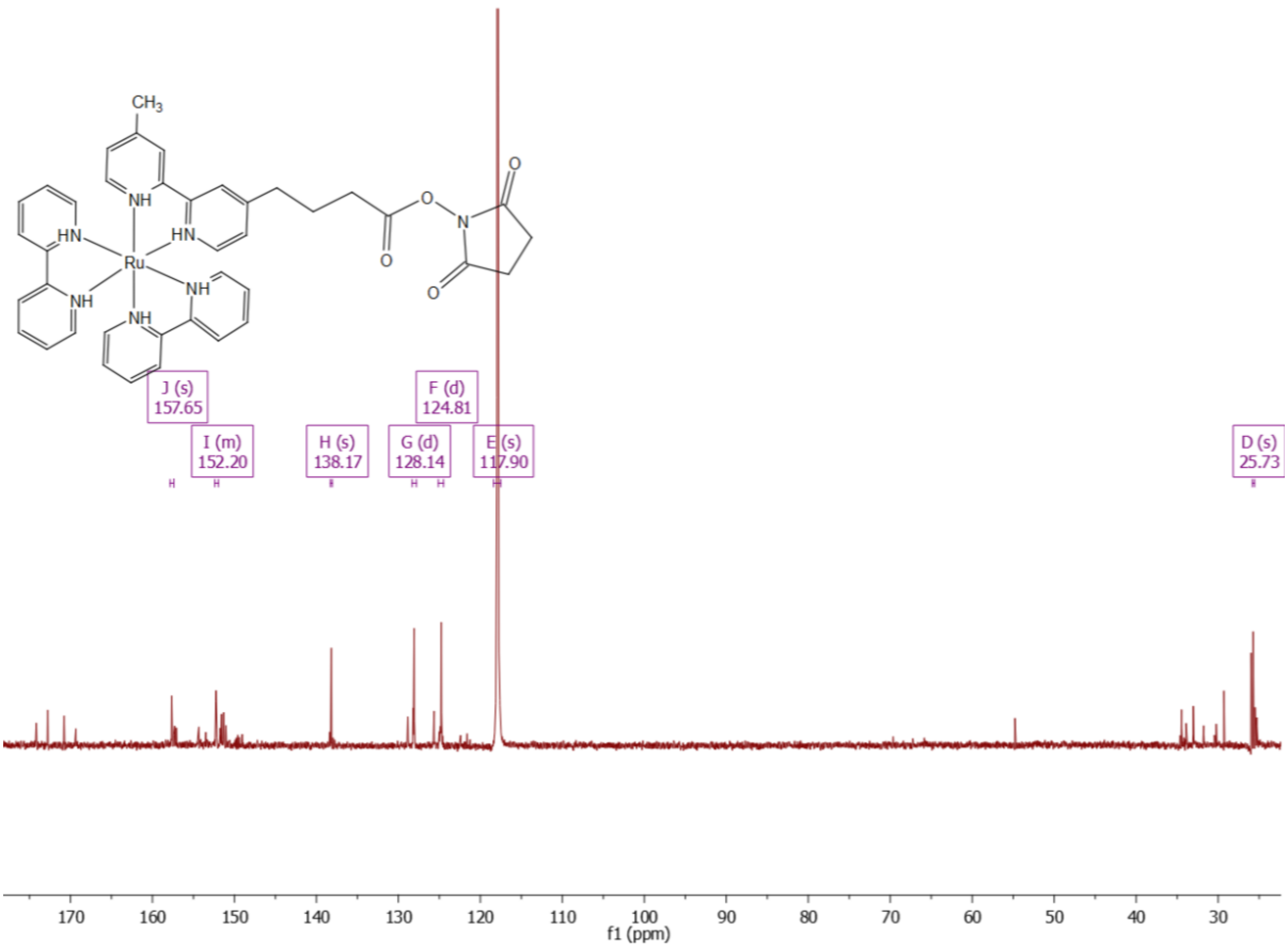

Figure S6. ^{13}C-NMR of Ru-NHS in CD_3CN.

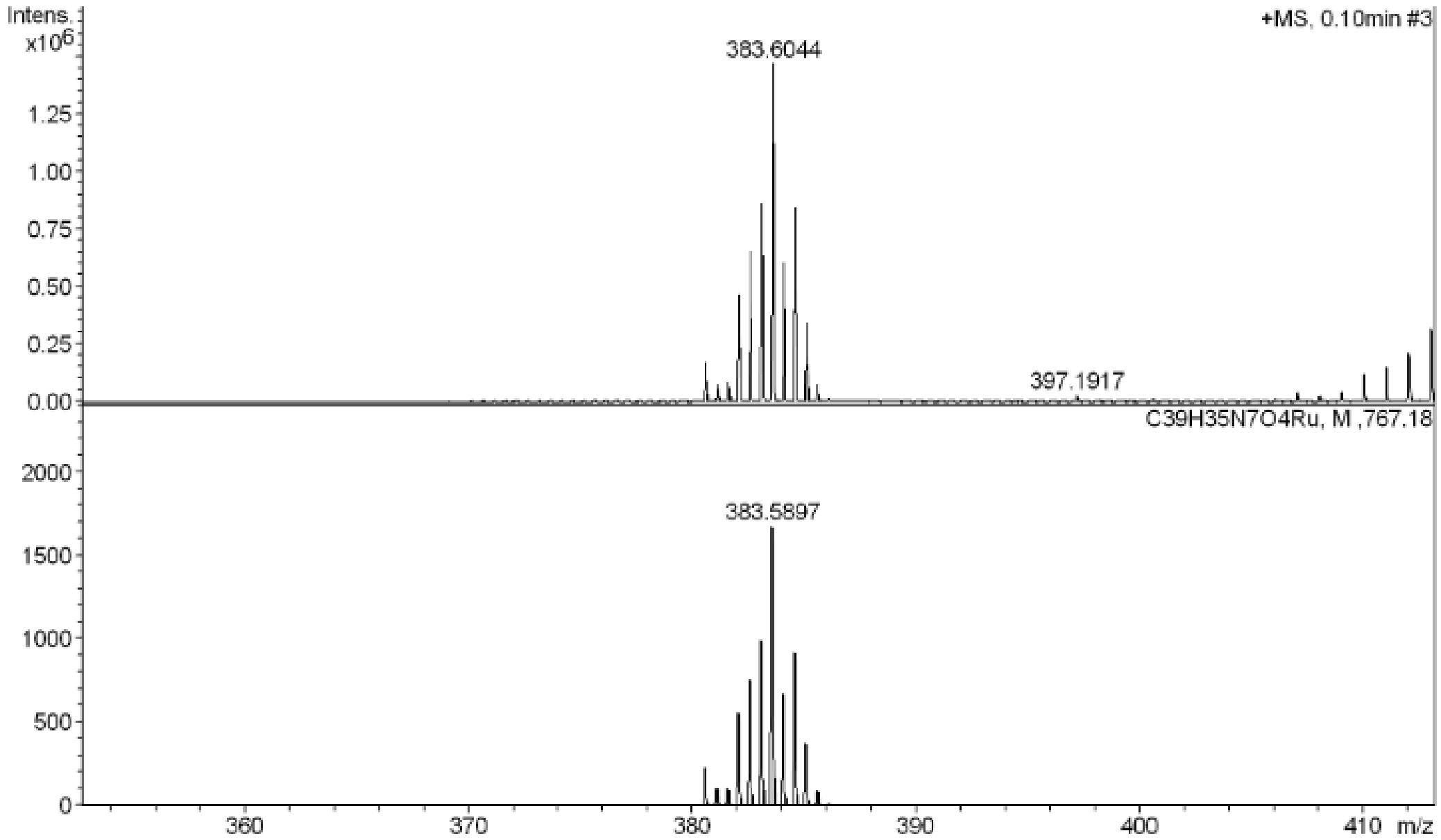

Figure S7. ESI-MS of Ru-NHS measured (top) and simulated (bottom).

Additional information can be found in the chemotion repository: https://dx.doi.org/10.14272/reaction/SA-FUHFF-UHFFFADPSC-GWDYGOLOEZ-UHFFFADPSC-NUHFF-NUHFF-NUHFF-ZZZ

Ru-N_3

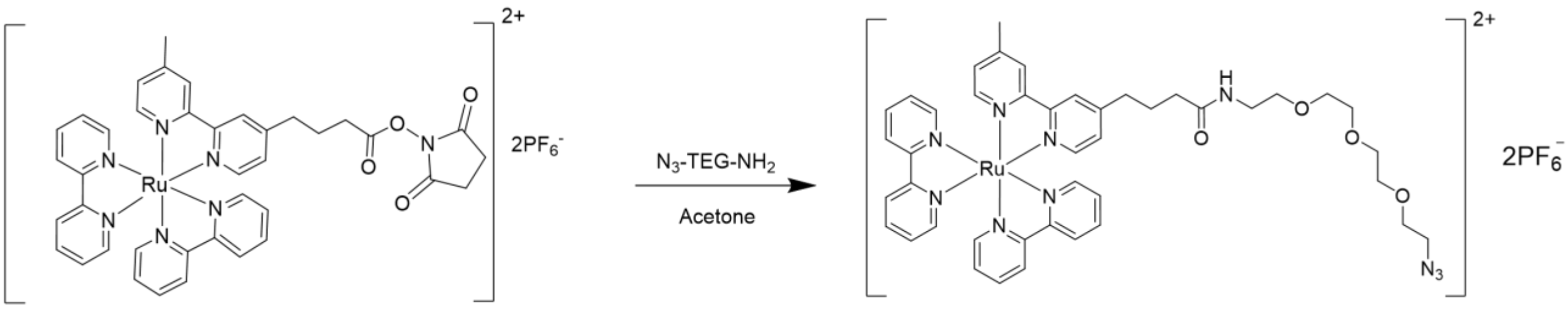

A 90 mM solution of 11-azido-trioxaundecan-1-amine in dry acetone (35.6 µL in 2 mL) was prepared, and Na_2SO_4 was added to this solution. 1600 µL (31.0 mg, 0.14 mmol 1.5 equiv.) of the linker solution was added to a solution of Ru-NHS (100 mg, 0.09 mmol, 1 equiv.) in 2 mL of dry acetone. The mixture was stirred overnight under N_2 atmosphere. Then, the product was precipitated by pouring in Et_2O (300 mL) and left for 3 h at 4° C. The crude product was obtained by centrifugation and then purified by neutral alumina column chromatography. (acetone/H_2O 7:1). The acetone was removed *in vacuo*, and the product was obtained after freeze-drying as a orange solid (98.6 mg, 0.085 mmol, 90%). ^{1}H-NMR (400 MHz, CD_3CN) δ: 8.46 (d, J = 8.2 Hz, 4H), 8.36 (d, J = 14.5 Hz, 2H), 8.07-7.98 (m, 4H), 7.70 (dd, J = 9.8, 4.9 Hz, 4H), 7.51 (t, J = 5.5 Hz, 2H), 7.38 (dddd, J = 11.2, 7.3, 4.4, 1.2 Hz, 4H), 7.26-7.17 (m, 2H), 6.41 (s, 1H), 3.60-3.48 (m, 10H), 3.43 (t, J = 5.6 Hz, 2H), 3.34-3.29 (m, 2H), 3.22 (dd, J = 11.0, 5.2 Hz, 2H), 2.83-2.74 (m, 2H), 2.51 (s, 3H), 2.19 (t, J = 7.2 Hz, 2H), 2.12-2.06 (m, 2H). ESI-MS: calcd. for $[M]^{2+}$ = 435.1404; found = 435.1464.

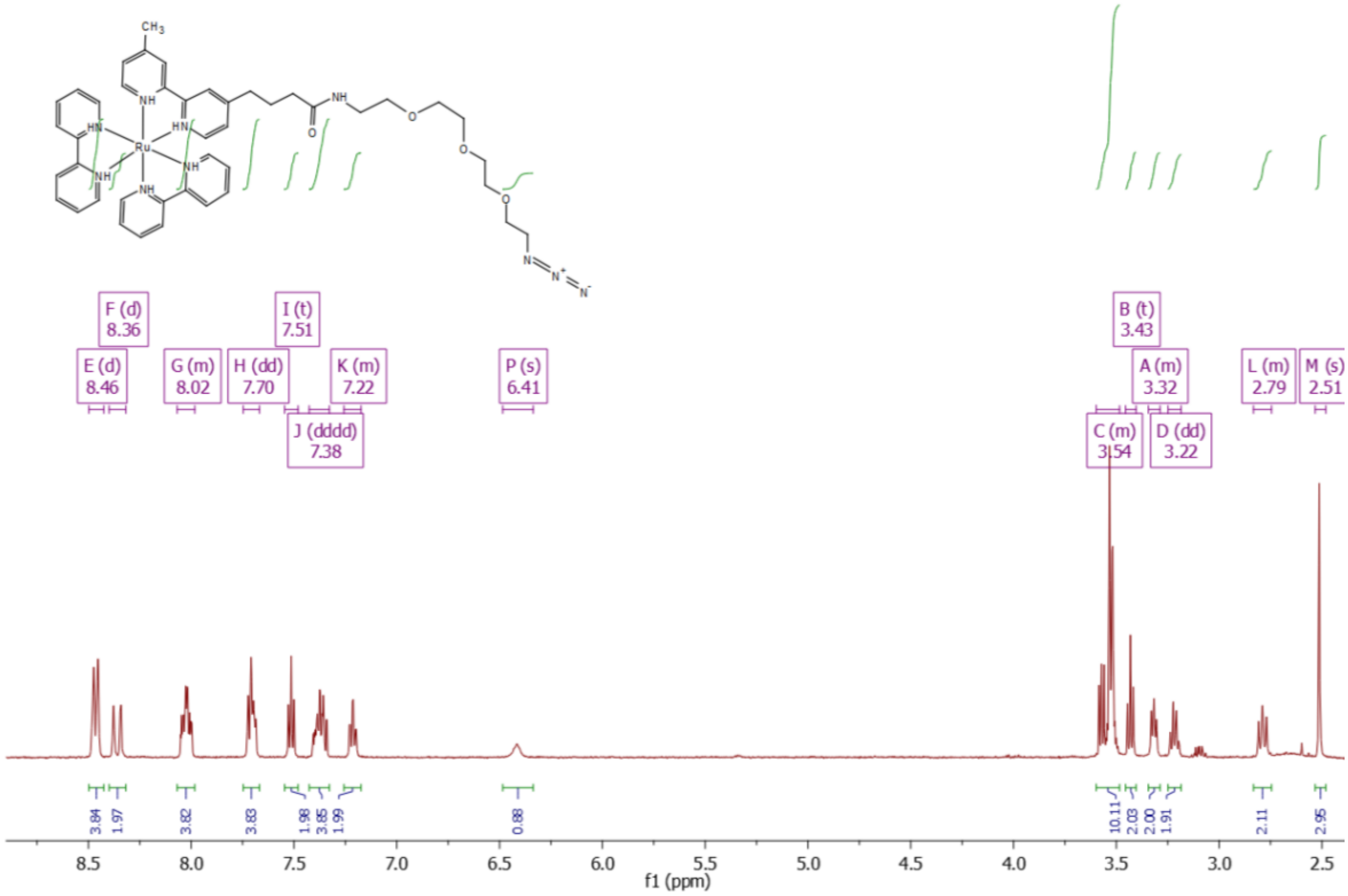

Figure S8. ^{1}H-NMR of Ru-N_3 in CD_3CN.

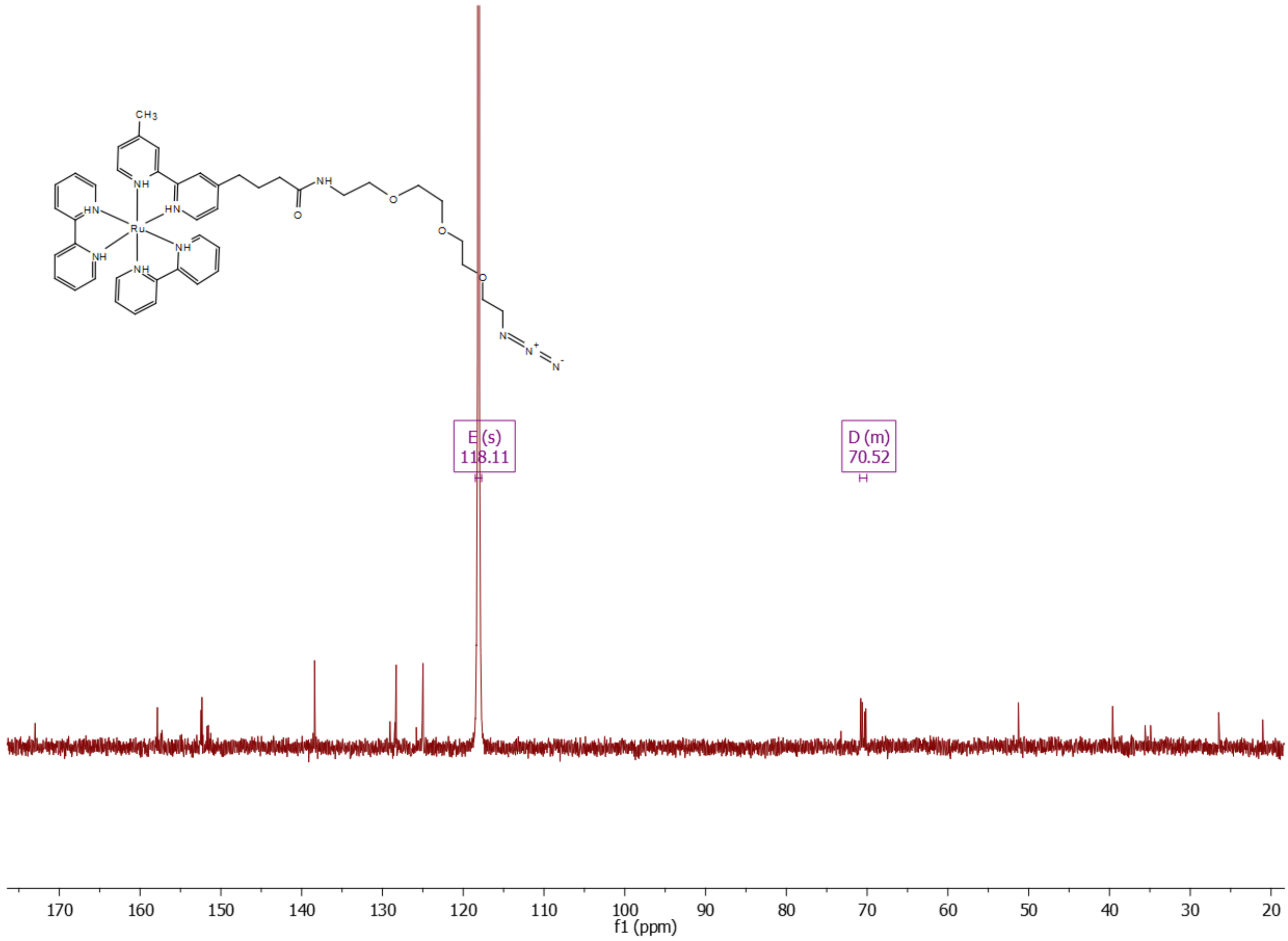

Figure S9. ^{13}C-NMR of Ru-N_3 in CD_3CN.

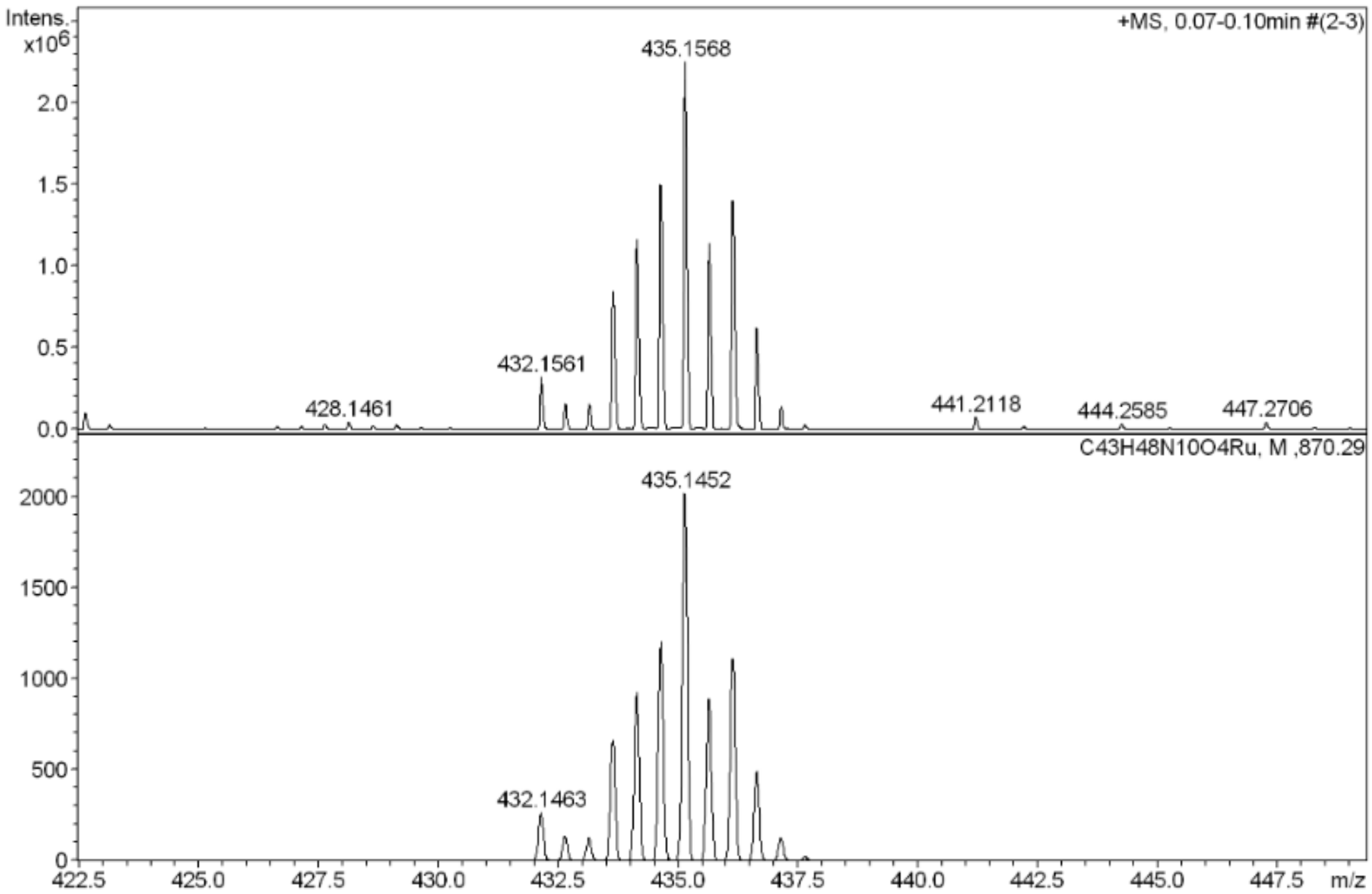

Figure S10. ESI-MS of Ru-N_3 measured (top) and simulated (bottom).

Additional information can be found in the chemotion repository: https://dx.doi.org/10.14272/reaction/SA-FUHFF-UHFFFADPSC-LLIZAOQNRC-UHFFFADPSC-NUHFF-NUHFF-NUHFF-ZZZ

3.1.3 Synthetic step c

CB7-Rubpy

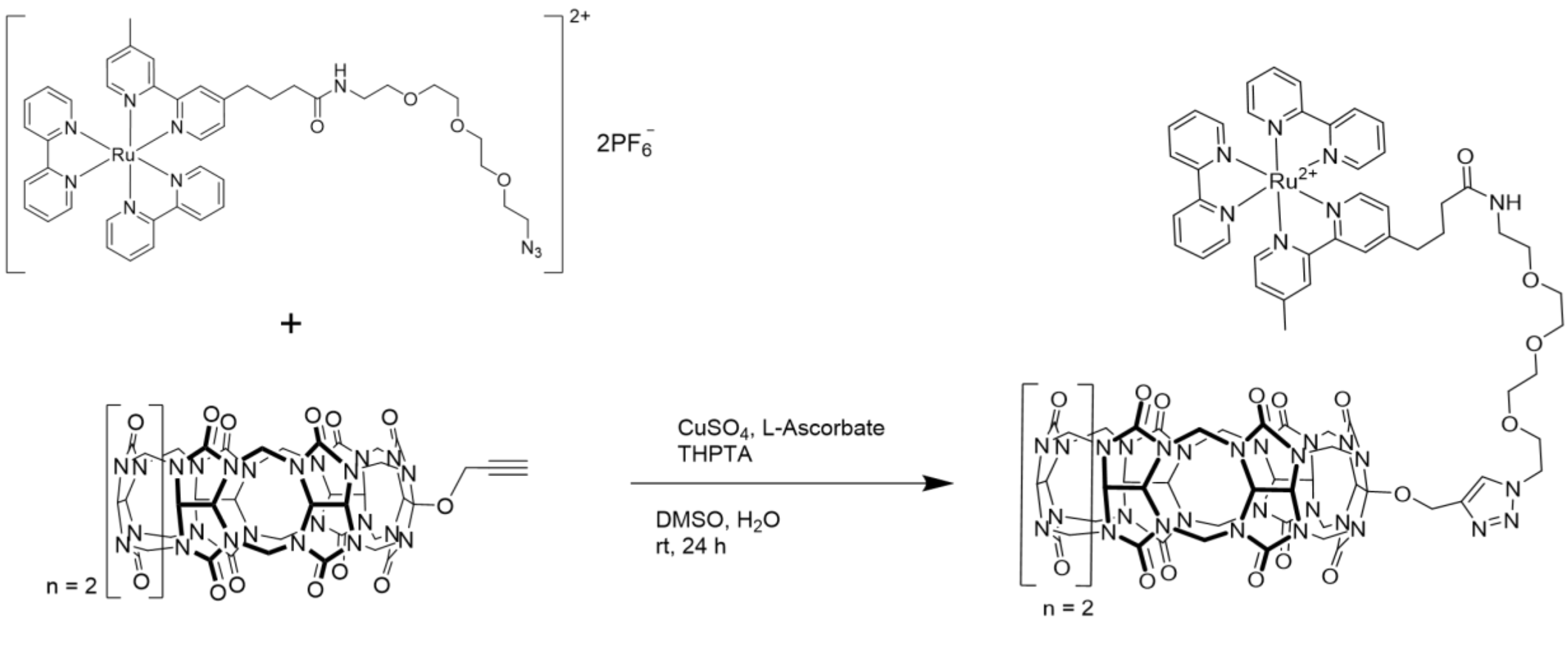

According to a modified literature procedure[11], in a vial, Ru-TEG-N_3 (21.4 mg, 0.018 mmol, 1.5 equiv.), CB7-OPr (15.0 mg, 0.012 mmol, 1 equiv.), and THPTA (21.4 mg, 0.050 mmol, 4 equiv.) were suspended in DMSO (1 mL). In a second vial, sodium L-ascorbate (24.4 mg, 0.123 mmol, 10 equiv.) was added to 5 mL 55% DMSO aqueous solution containing $CuSO_4$ (11.8 mg, 0.074 mmol, 6 equiv.). The two solutions were mixed and stirred at room temperature for 24 h, in the dark, under an N_2 atmosphere. Afterwards, the product was precipitated by addition of the reaction solution in MeOH/THF 1:1. Washing with MeOH/THF (1:1, v/v) afforded the crude product, which was purified by flash chromatography on MCI GEL CHP20P using an H_2O/ACN gradient of 0–25% ACN. ^{1}H-NMR (400 MHz, D_2O) δ: 8.50 (d, J = 8.0 Hz, 4H), 8.36 (s, 2H), 8.10 (d, J = 11.2 Hz, 1H),

8.00 (t, J = 7.5 Hz, 4H), 7.79 (dd, J = 10.3, 5.4 Hz, 4H), 7.62 (dd, J = 18.1, 5.8 Hz, 2H), 7.34 (dd, J = 13.2, 7.1 Hz, 4H), 7.20 (dd, J = 10.5, 5.9 Hz, 2H), 5.99-5.33 (m, 26H), 4.47-4.14 (m, 14H), 3.91-3.82 (m, 2H), 3.48 (dq, J = 10.7, 5.5 Hz, 12H), 3.15-3.00 (m, 2H), 2.80 (t, J = 7.0 Hz, 2H), 2.64-2.55 (m, 4H), 2.48 (s, 3H), 2.27 (t, J = 7.0 Hz, 2H), 2.02-1.90 (m, 2H), 1.26-1.09 (m, 6H). Due to the complex structure, a precise assignment of all peaks was not possible. Even after multiple attempts, it was not possible to measure the ^{13}C-NMR spectrum of the compound. ESI-MS: calcd. for $[M+C_8(mim)_2]^{4+}$ = 590.7191; found = 590.7195. Analyt. HPLC: 4.98 min.

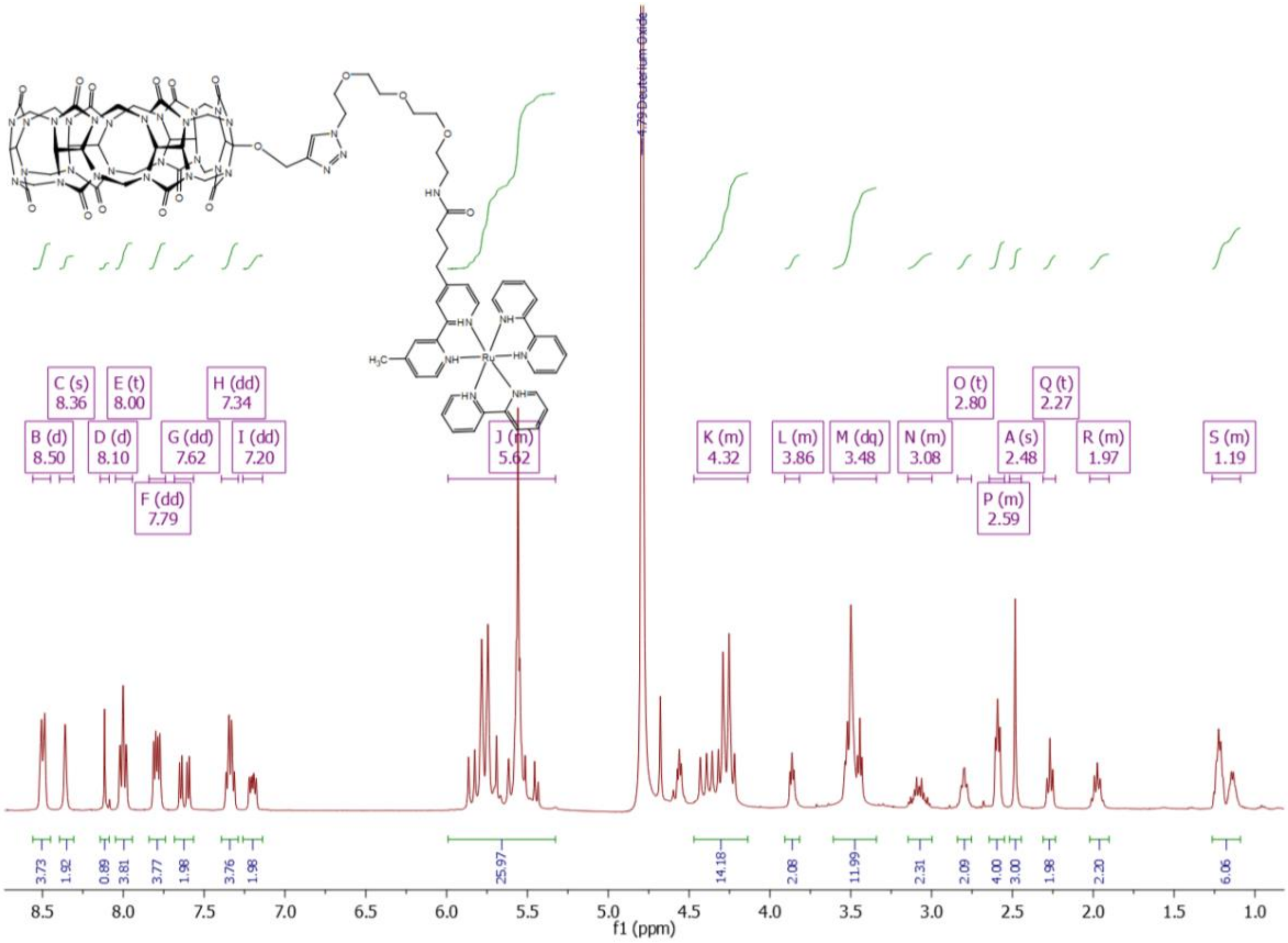

Figure S11. ^{1}H-NMR of CB7-Rubpy in D_2O.

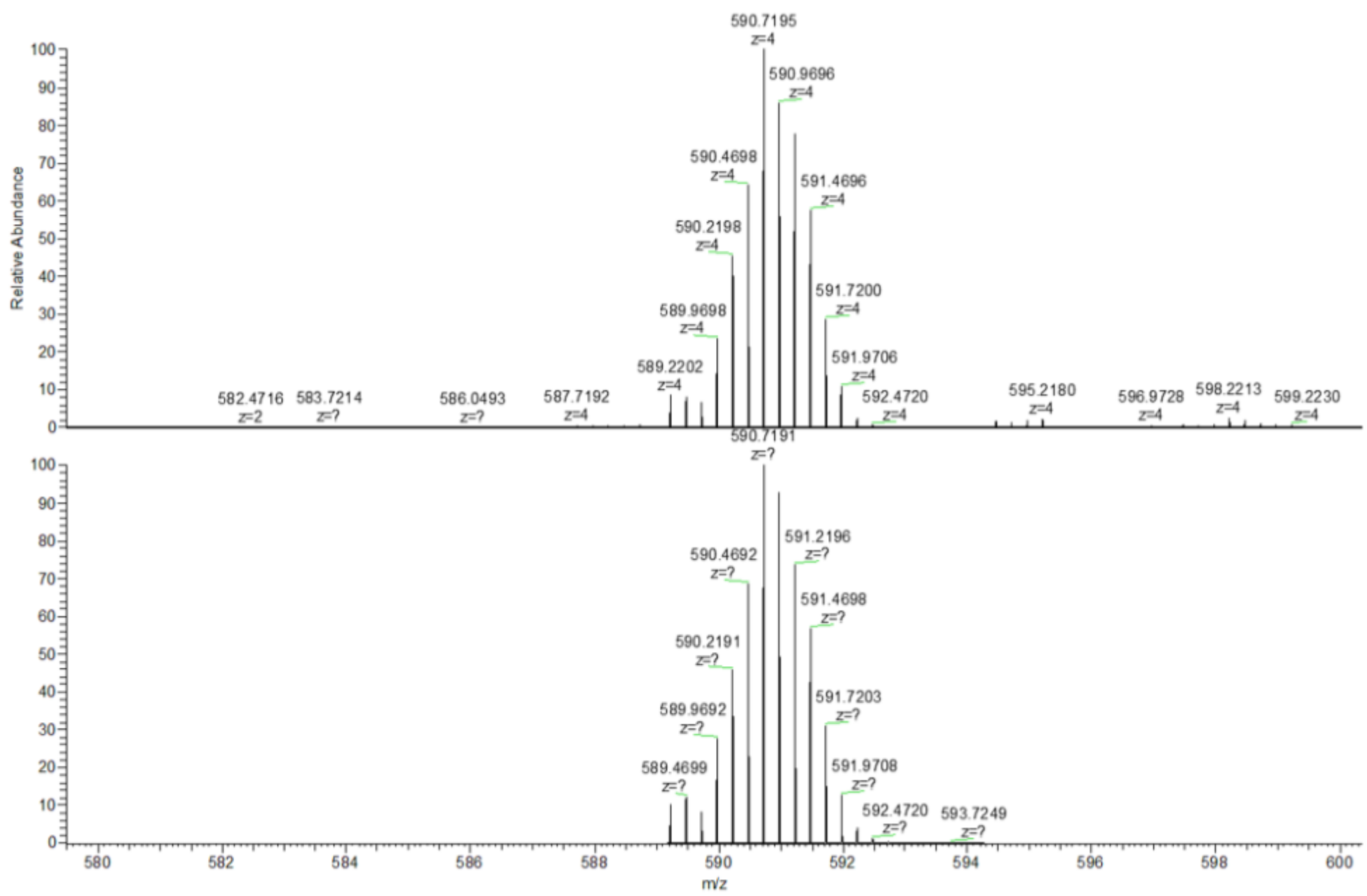

Figure S12. ESI-MS of $[CB7\text{-}Rubpy{\bullet}C_8(mim)_2]^{4+}$ adduct, measured (top) and simulated (bottom).

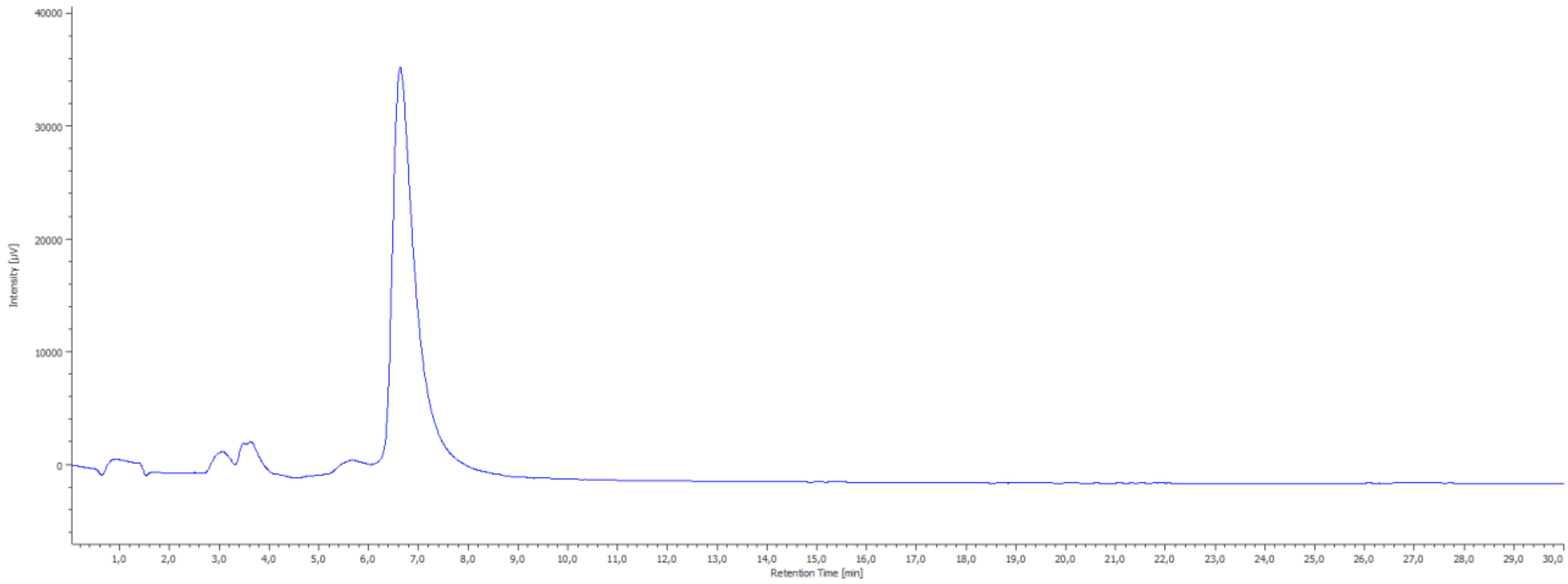

Figure S13. Analytical trace of CB7-Rubpy.

Additional information can be found in the chemotion repository: https://dx.doi.org/10.14272/reaction/SA-FUHFF-UHFFFADPSC-RWLGBBMHCA-UHFFFADPSC-NUHFF-NUHFF-NUHFF-ZZZ

3.1.4 Ada-Ru

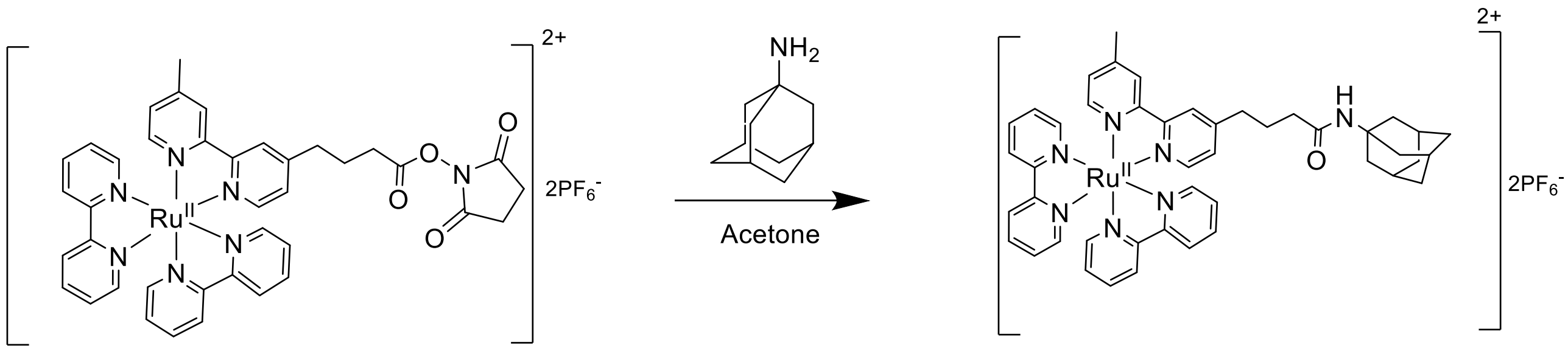

1-adamantamine (28.6 mg, 0.19 mmol 2 equiv.) and 50 µL Triethylamine were dissolved in dry acetone (1 mL). Ru-NHS (100 mg, 0.09 mmol 1 equiv.) was then added and the mixture was purged with N_2. The mixture was stirred overnight under an atmosphere of N_2. Then, the product was precipitated by pouring in Et_2O (200 mL). This was left for 3h in the fridge. The crude product is obtained by centrifugation. It was then purified by neutral alumina column chromatography. (Acetone/H_2O 7:1). The product was obtained as a dark orange solid. (70.9 mg, 0.06 mmol, 69%) ^{1}H NMR (400 MHz, CD_3CN) δ 8.48 (d, J = 8.1 Hz, 4H), 8.41 - 8.31 (m, 2H), 8.09 - 7.98 (m, 4H), 7.72 (t, J = 6.0 Hz, 4H), 7.53 (dd, J = 7.9, 5.8 Hz, 2H), 7.44 - 7.33 (m, 4H), 7.23 (dd, J = 5.5, 2.2 Hz, 2H), 2.83 - 2.75 (m, 2H), 2.53 (s, 3H), 2.11 (dt, J = 8.7, 5.0 Hz, 4H), 2.00 (s, 3H), 1.92 - 1.87 (m, 2H), 1.72 - 1.60 (m, 7H), 1.18 (d, J = 3.8 Hz, 3H). ESI-MS: calcd. for $[M-2PF_6]^{2+}$ = 401.6437; found = 401.6429

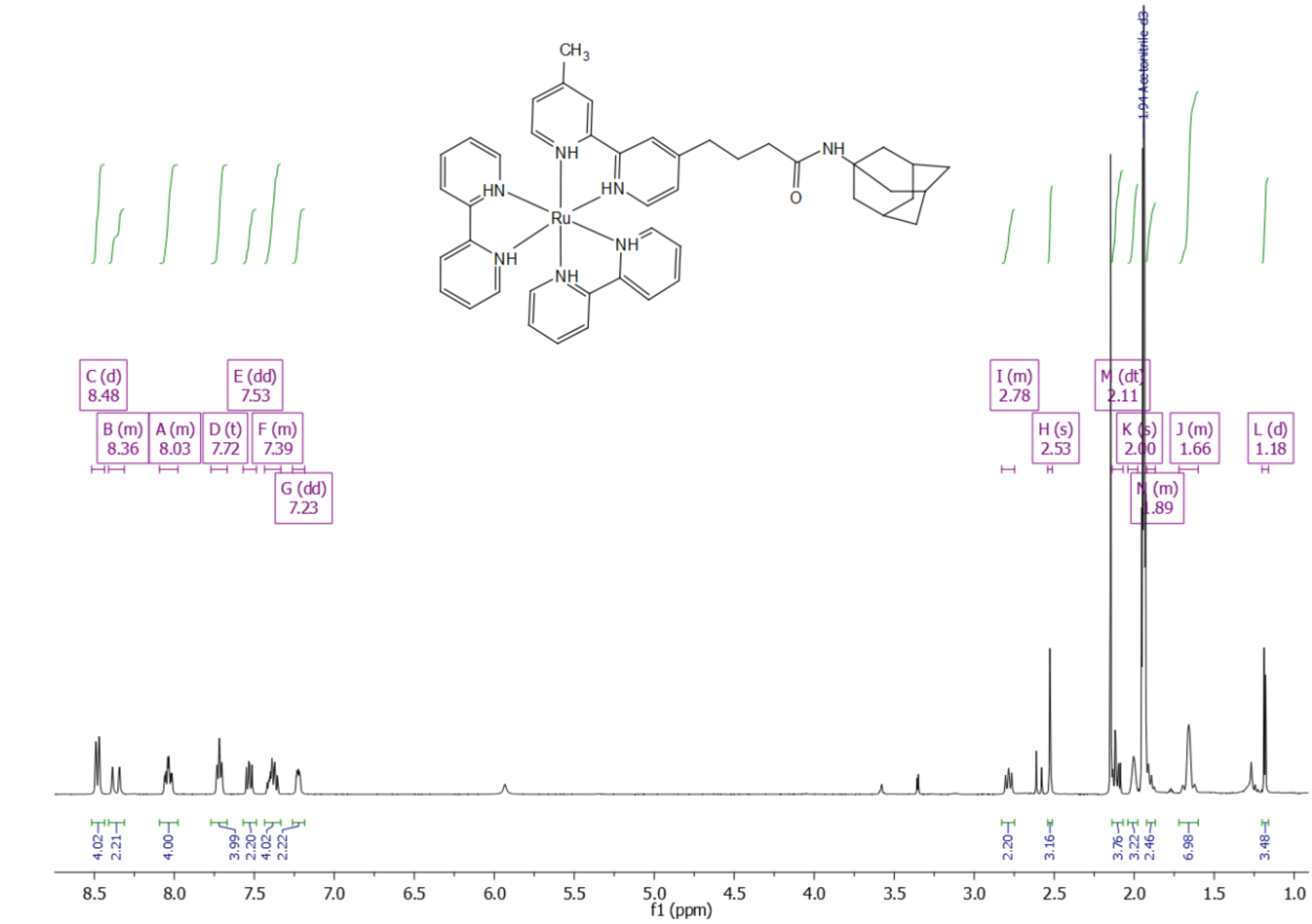

Figure S14. ^{1}H-NMR of Ada-Ru in CD_3CN.

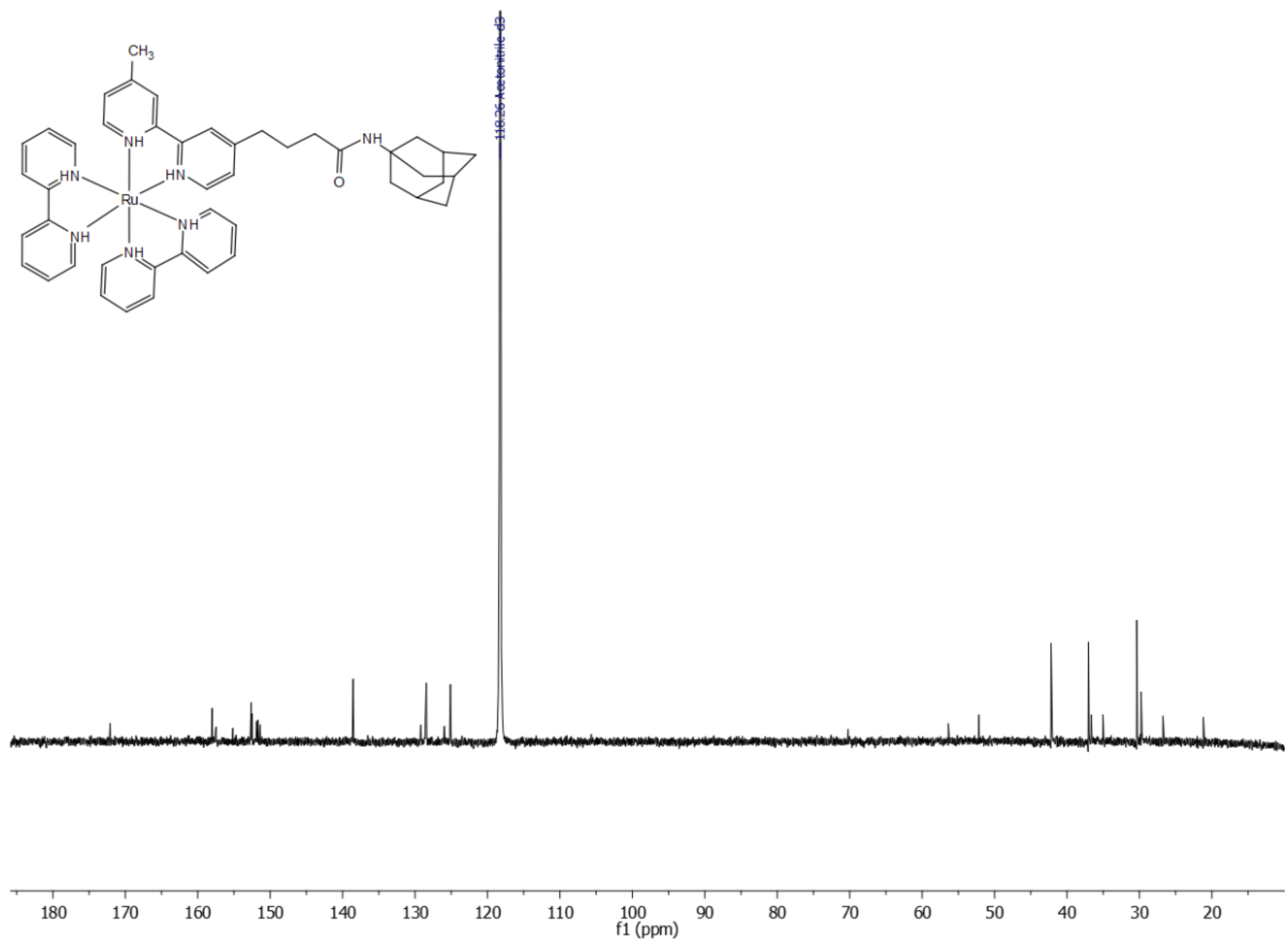

Figure S15. ^{13}C NMR of Ada-Ru in CD_3CN

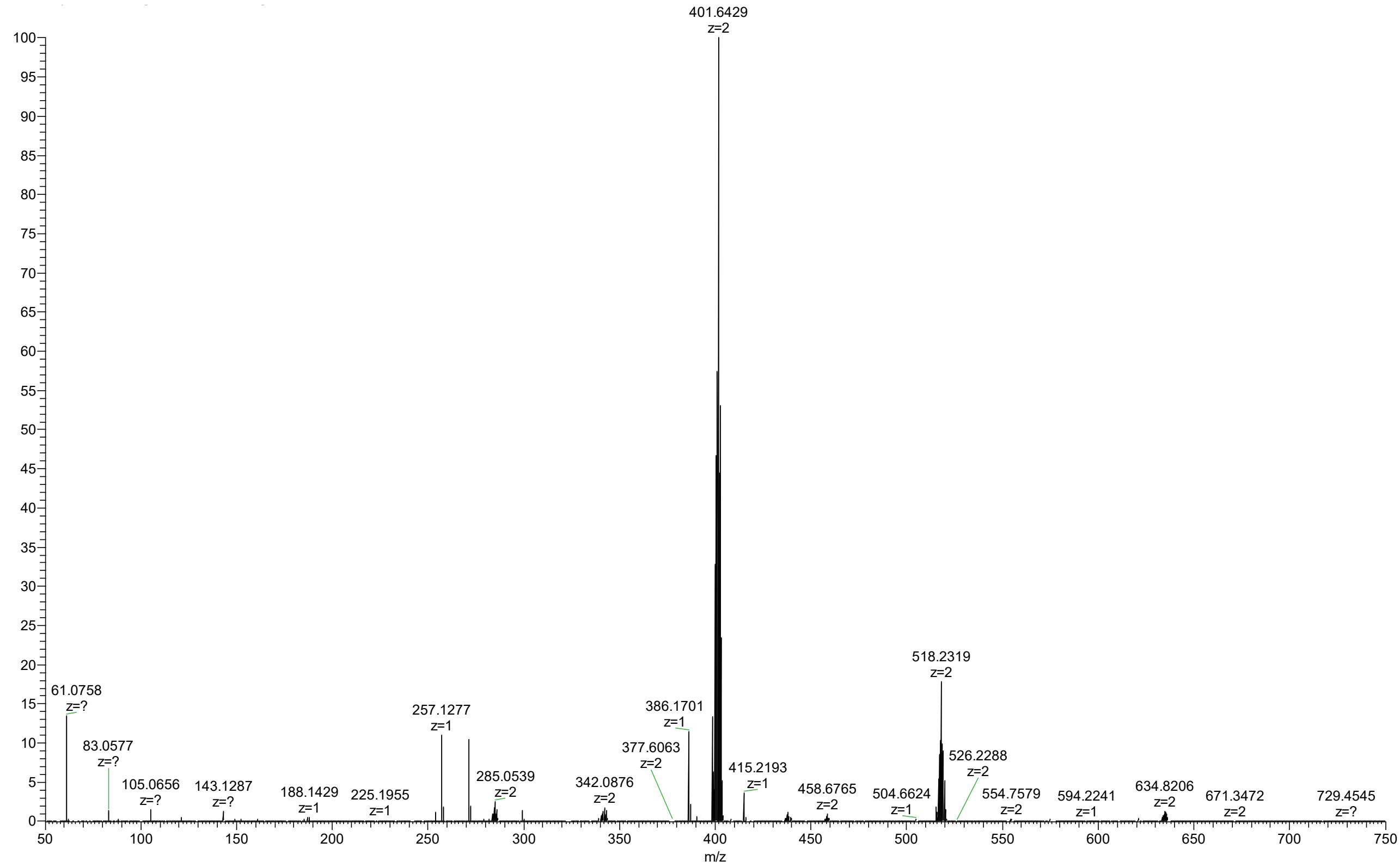

Figure S16. ESI MS of Ada-Ru.

Additional information can be found in the chemotion repository:
https://dx.doi.org/10.14272/reaction/SA-FUHFF-UHFFFADPSC-RBYPRXJKYM-UHFFFADPSC-NUHFF-NRWFG-NUHFF-ZZZ

3.2 Experimental details

3.2.1 Emission-based detection

In a typical procedure, a stock solution of CB7-Rubpy (2 mM in water) was diluted to 5 µM for the protein sensing experiments and to 1 µM for the binding constant experiments (in the considered solvent) in a PMMA cuvette (optical path length = 1 cm), to which a solution of the analyte was added in increasing concentrations. For each addition, the solution in the cuvette was stirred with the aid of a magnetic stirrer in order to equilibrate for 2 minutes. Emission spectra were recorded upon excitation at 452 nm, corresponding to the ^{3}MLCT absorption band of the compound. The changes in emission intensity occurring around 620 nm were investigated (excitation slit width = 5 nm, emission slit width = 2 nm, emission cut-off = 500 nm).

3.2.2 Quantum chemical methods and calculation of collision cross sections.

For calculating structural conformer candidates, we used the GFN2-xTB[7] method and additionally the aISS docking submodel for host-guest complexes[12] as well as the Conformer-Rotamer Ensemble Sampling Tool (CREST).[6] More reliable electronic energies were computed with DFT and the r^{2}SCAN-3c method[8] in TURBOMOLE 7.8, employing the optimized GFN2-xTB geometries as input structures. Model structures were initially assembled in Avogadro (version 1.2.0) and pre-optimized using the universal force field (UFF). Additionally, for the host-guest complexes we performed implicit solvation calculations with COSMO[13] as implemented in TURBOMOLE. A dielectric constant of ε_r = 59 (representing a 1:1 mixture of methanol and water) was used, together with default atomic radii.

The calculation of collision cross sections ($^{TM}CCS_{N2}$) was performed with IMoS 1.09 using the trajectory method (TM) in N_2 at 300 K and the ion-induced quadrupole potential was always considered.[14, 15] Mulliken partial charges from xTB and DFT were used for modelling the electrostatic interactions within the TM method. To minimize the statistical error to < 0.05 % we calculated 3 x 10^7 trajectories in total.

We used the $^{TM}CCS_{N2}$ values calculated from the GFN2-xTB structures, which were approximately 3% larger than the experimentally determined $^{tims}CCS_{N2}$ values. The values calculated from the r²SCAN-3c structures were consistently approximately 5% larger. Similar deviations between calculated and experimental CCS values have been reported for related systems.[16]

The GFN2-xTB structures and their r²SCAN-3c-reoptimized counterparts agree well, with root-mean-square deviations (RMSDs) of 0.32–0.45 Å for structures P1–P5. The better agreement of the GFN2-xTB-based $^{TM}CCS_{N2}$ values with experiment most likely reflects error cancellation and sensitivity to the atomic-charge model; values obtained using natural bond orbital (NBO) charges were approximately 2% larger than those obtained using Mulliken charges.

3.2.3 Calculation of the degree of complexation

The degree of complexation (θ) is defined as the fraction of host molecules present in the complexed host-guest state at equilibrium. For the present calculation, a 1:1 host-guest model (H + G ⇌ HG) is assumed, where the host (H) is defined as the folded CB7-Rubpy•M^+ complex, the guest (G) as the metal cation M^+, and the open complex (HG) as CB7-Rubpy•2M^+. The degree of complexation with respect to the host is given by θ_H=[HG]/$[H]_0$ where [HG] is the equilibrium concentration of the CB7-Rubpy•2M^+complex, and $[H]_0$ is the initial concentration of CB7-Rubpy•M^+. Thus, θ represents the ratio of open CB7-Rubpy•2M^+ to folded CB7-Rubpy•M^+species. For the calculation of the degree of

complexation in phosphate buffer (PB), a Na^+ concentration of 17 mM was assumed. For phosphate-buffered saline (PBS), Na^+ and K^+ concentrations of approximately 150 mM and 4.5 mM, respectively, were used.

3.2.4 Structure-guided assessment of phenylalanine accessibility

To rationalize the protein-dependent emission responses of CB7-Rubpy, we performed a structure-guided screening of phenylalanine accessibility in representative protein structures or structural models. This analysis was used to identify potentially accessible aromatic motifs and to exclude clearly buried phenylalanine residues. It was not intended to assign definitive CB7-Rubpy binding sites.

Atomic coordinates were obtained in PDBx/mmCIF format from the Protein Data Bank or, where no experimental structure of the exact protein was available, from the corresponding predicted structural model. The following structures or models were analysed: human serum albumin (HSA, PDB 1AO6), bovine HYAL1 as a representative sequence/model reference for the bovine testicular hyaluronidase preparation (UniProt Q5E985 and corresponding predicted structural model), Pseudomonas/Burkholderia cepacia lipase in the open conformation (PDB 3LIP), hen egg-white lysozyme (PDB 1DPX), and bovine pancreatic RNase A (PDB 7RSA). For HSA, both crystallographically independent chains of PDB 1AO6 were analysed, and residue numbers refer to the mature HSA sequence.

Structures were prepared by removing crystallographic water molecules, free ions, buffer components, glycerol, acetate, tert-butanol, and other non-covalently bound crystallization additives. Hydrogen atoms were omitted for consistency. No energy minimization, protonation-state optimization, or side-chain rebuilding was performed. For alternate conformations, the highest-occupancy conformer was used where applicable. Resolved covalently attached glycans were retained when present, because they can sterically shield protein surfaces.

For each phenylalanine residue, we calculated the solvent-accessible surface area (SASA) of the six aromatic ring carbon atoms, CG, CD1, CD2, CE1, CE2, and CZ. This phenyl-ring SASA was used instead of whole-residue SASA because supramolecular recognition by CB7-type receptors depends primarily on the accessibility of the aromatic surface rather than on backbone or Cβ exposure. SASA values were calculated using a numerical Shrake–Rupley-type algorithm with a 1.40 Å solvent probe and standard van der Waals radii. Surface points were distributed using Fibonacci sampling, and neighbour searches were accelerated using a three-dimensional cKDTree. The phenyl-ring SASA was obtained as the sum of the SASA values of the six ring carbon atoms.

Residues with phenyl-ring SASA ≥30 Å² were classified as clearly exposed aromatic candidates, residues with 20–30 Å² as moderately exposed, residues with 10–20 Å² as marginally or edge-exposed, and residues with <10 Å² as effectively buried for supramolecular recognition. These thresholds are empirical screening criteria and should not be interpreted as strict physical boundaries.

The analysis identified F49, F374, and F395 as the most robustly exposed phenylalanine candidates in HSA, with F206, F228, and F502 as additional moderate-to-strong candidates. For bovine HYAL1, used as a representative sequence/model reference for the bovine testicular hyaluronidase preparation, F47, F81, F108, and F125 were identified as plausible accessible aromatic candidates. In the open conformation of Pseudomonas/Burkholderia cepacia lipase, F119, F122, F142, F146, F221, and F249 were the most exposed phenylalanine residues, although this assignment is

expected to depend strongly on the conformational state of the lipase. Lysozyme contained only one clearly exposed phenylalanine candidate, F34, whereas RNase A contained no clearly surface-accessible phenylalanine by this criterion, with F120 classified only as marginally exposed.

Importantly, solvent exposure alone does not demonstrate productive CB7-Rubpy binding. SASA calculations use a water-sized probe, whereas CB7 is a much larger receptor and requires an accessible approach trajectory, sufficient space around the aromatic face, compatible portal electrostatics, suitable side-chain mobility, and absence of steric shielding by neighbouring loops, glycans, cofactors, or other surface features. Thus, this analysis provides a first structure-based rationale for the observed protein-dependent responses, but definitive binding-site assignment would require mutagenesis, peptide mapping, docking, or molecular dynamics simulations.

3.2.5 Chemical denaturation of proteins

Protein denaturation was performed using a chaotropic/reducing mixture consisting of 6 M urea and 2 mM DTT. Proteins were adjusted to a final concentration of 100 µM in a total volume of 1 mL and incubated for 30 min at room temperature. Protein concentrations before and after denaturation were determined by UV absorbance at 280 nm using the following extinction coefficients (ε280): insulin, 5,700 M^{-1} cm^{-1}; RNase A, 9,800 M^{-1} cm^{-1}; HSA, 36,500 M^{-1} cm^{-1}; lysozyme, 37,000 M^{-1} cm^{-1}.

Denaturation–recovery experiments were performed independently in duplicate from the same protein stock solution. After recovery, samples were subjected to buffer exchange using PD MidiTrap G-25 desalting columns (Cytiva) according to the manufacturer's instructions. A process blank was generated by performing the identical desalting procedure in the absence of protein and was used for baseline correction to account for residual denaturants remaining after desalting.

For each independent denaturation experiment, two fluorescence samples were prepared and measured, resulting in four fluorescence measurements per condition (two preparation replicates and two technical replicates). Emission measurements were performed at final concentrations of 5 µM CB7-Rubpy and 5 µM protein.

The scheme below summarizes the chemical and thermal denaturation workflows.

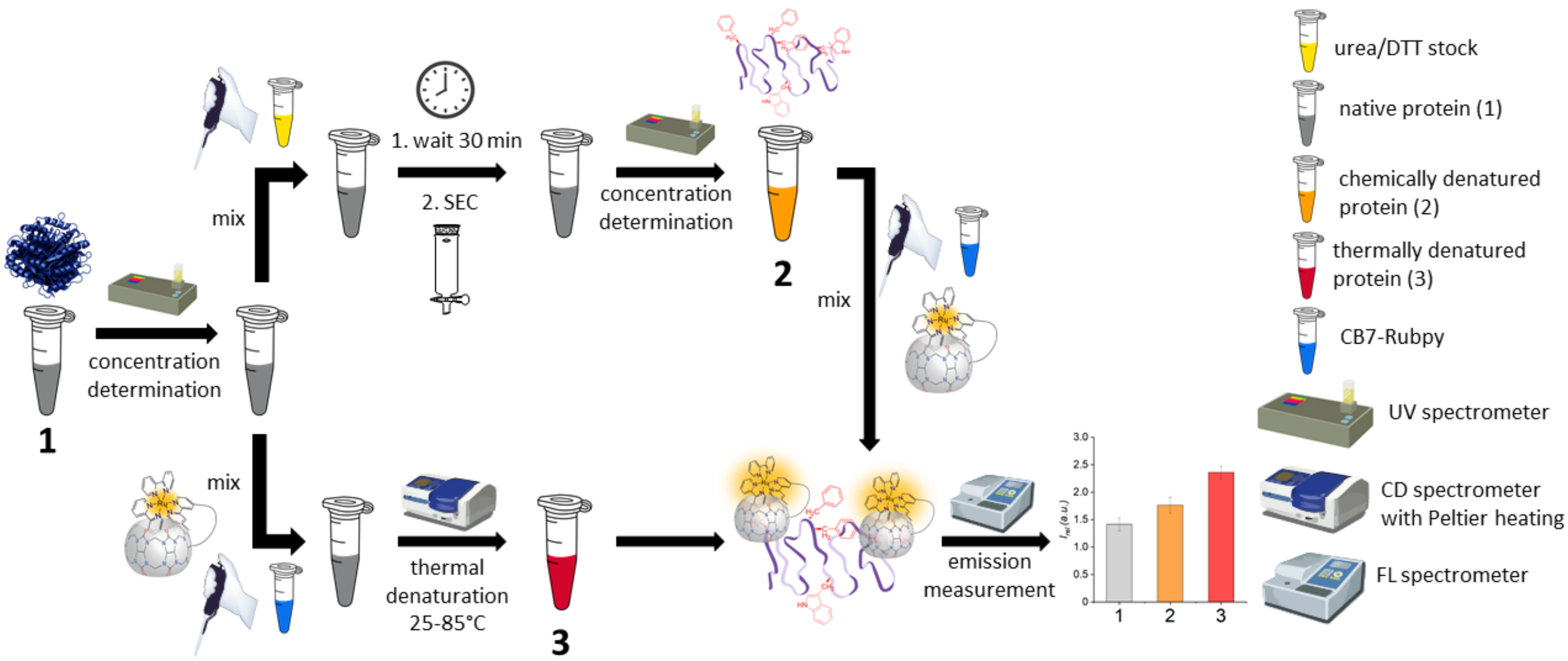

Figure S17. Workflow for chemical and thermal protein denaturation. Protein concentrations were first determined by UV absorption to obtain native protein solutions. For chemical denaturation, proteins were incubated with urea (6 M) and DTT

(2 mM) for 30 min at room temperature. The denaturants were subsequently removed by size-exclusion chromatography, and protein concentrations were re-determined. CB7-Rubpy was then added and emission measurements were performed.

For thermal denaturation, native protein solutions were mixed with CB7-Rubpy and subjected to a heating-cooling cycle. Emission measurements were carried out after thermal treatment.

3.2.6 Emission titrations with salt solutions

Emission titrations for determination of metal-ion binding affinities were performed using concentrated stock solutions prepared in Milli-Q water. Stock solutions were generated from the corresponding metal nitrate salts to enable concentration determination by UV–Vis spectroscopy. Nitrate concentrations were quantified by UV–Vis spectroscopy at 201 nm (ε = 9500 M^{-1} cm^{-1} in water[17]), and the resulting concentrations were used to prepare the stock solutions employed in all titration experiments.

4. Supporting Tables

Cations	*r* (Å)	Log K_{a2}, CB7-Rubpy	Log K_a, CB7
Li^+	0.69	n.a.	0.37
Na^+	1.02	0.54±0.09	3.53
K^+	1.38	0.64±0.09	3.46
Mg^{2+}	0.72	n.a.	0.51
Eu^{3+}	1.09	2.81±0.11	3.30
Gd^{3+}	1.19	2.60±0.12	2.93

Table S1. Extracted binding affinities (K_{a2}) of metal cations with CB7-Rubpy in water. The reported values correspond to the second, weaker binding event associated with disruption of the portal-associated CB7-Rubpy interaction and transition to the solvent-exposed (open) chromophore state. The concentration of CB7-Rubpy was kept constant at 1 µM. Reference values for native CB7 were taken from ref [18].

Analyte	Charge	log K_a (CB7)		log K_a (CB7-Rubpy)		Signal factor (CB7-Rubpy)	
		DI water	*PB*	*DI water*	*PB*	*DI water*	*PB*
M_2V	2^+	8.78 [19]	6.82[20]	6.31±0.09	6.58±0.14	0.1	3.4×10^{-4}
Cad	2^+	7.41[21]	6.65[22] [a]	5.92±0.03	5.35±0.11	6.3	4.0
Spd	3^+	5.31[23]	n.a.	5.29±0.03	5.15±0.01	5.3	3.2
Spm	4^+	7.41[21]	6.07[24] [b]	6.10±0.04	5.80±0.04	6.7	6.7
Phe	zwitterionic	6.01[25]	5.06[26]	5.55±0.07	5.08±0.03	5.4	2.9
Phe-Gly	zwitterionic	7.51±0.01	7.08±0.05	7.71±0.10	7.60±0.30	3.7	3.0
insulin	varies	n.a.	6.18[27]	n.a.	7.19±0.28	n.a.	2.7

Table S2. Comparison of binding affinities in deionized water and phosphate buffer (PB, 10 mM, pH 7.4). Association constants for CB7-Rubpy and the corresponding signal factors were obtained from emission titrations fitted to a 1:1 binding model (λ_{exc} = 452 nm, λ_{em} = 610 nm). Values for native CB7 were taken from the cited literature except for Phe-Gly, which was determined by indicator displacement assays under matched medium conditions (Figures S64 and S65). [a]10 mM ammonium phosphate. [b]20 mM sodium phosphate buffer.

5. Supporting Figures

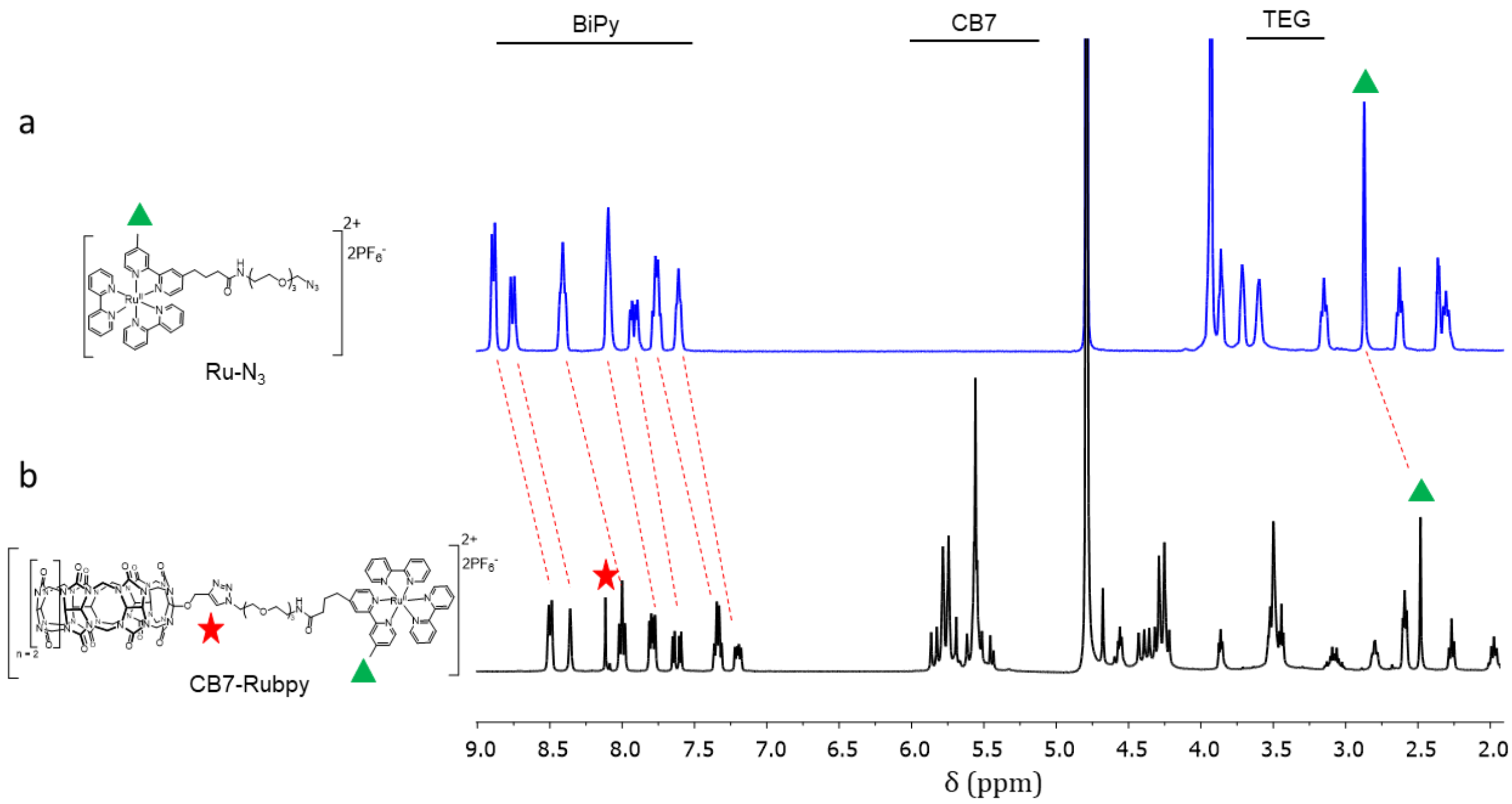

Figure S18. Overlay of ^{1}H-NMR spectra (400 MHz, in D_2O) of a) the azide-functionalized complex of the Ru(II) precursor, i.e., Ru-N_3 and b) CB7-Rubpy. The methyl group of the connecting bipyridine ligand is marked with a green triangle, while the triazole proton is marked with a red star.

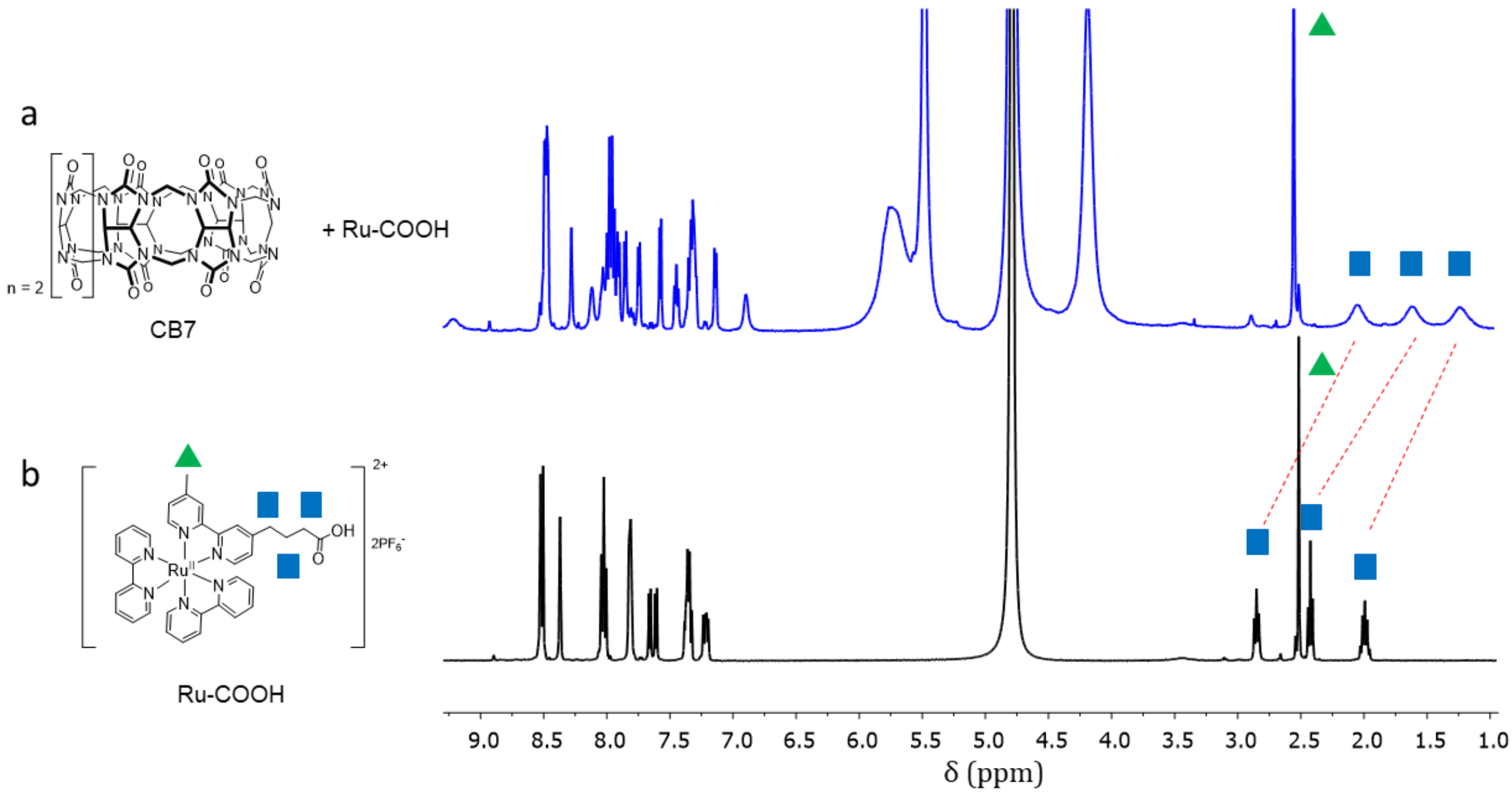

Figure S19. Overlay of ^{1}H NMR spectra (400 MHz, D_2O) of (a) CB7 + Ru-COOH (1:1 mixture, 2 mM each) and (b) Ru-COOH (2 mM). The methyl group of the linker-adjacent bipyridine ligand is marked with a green triangle, while the protons of the alkyl linker on Ru-COOH are indicated by a blue square.

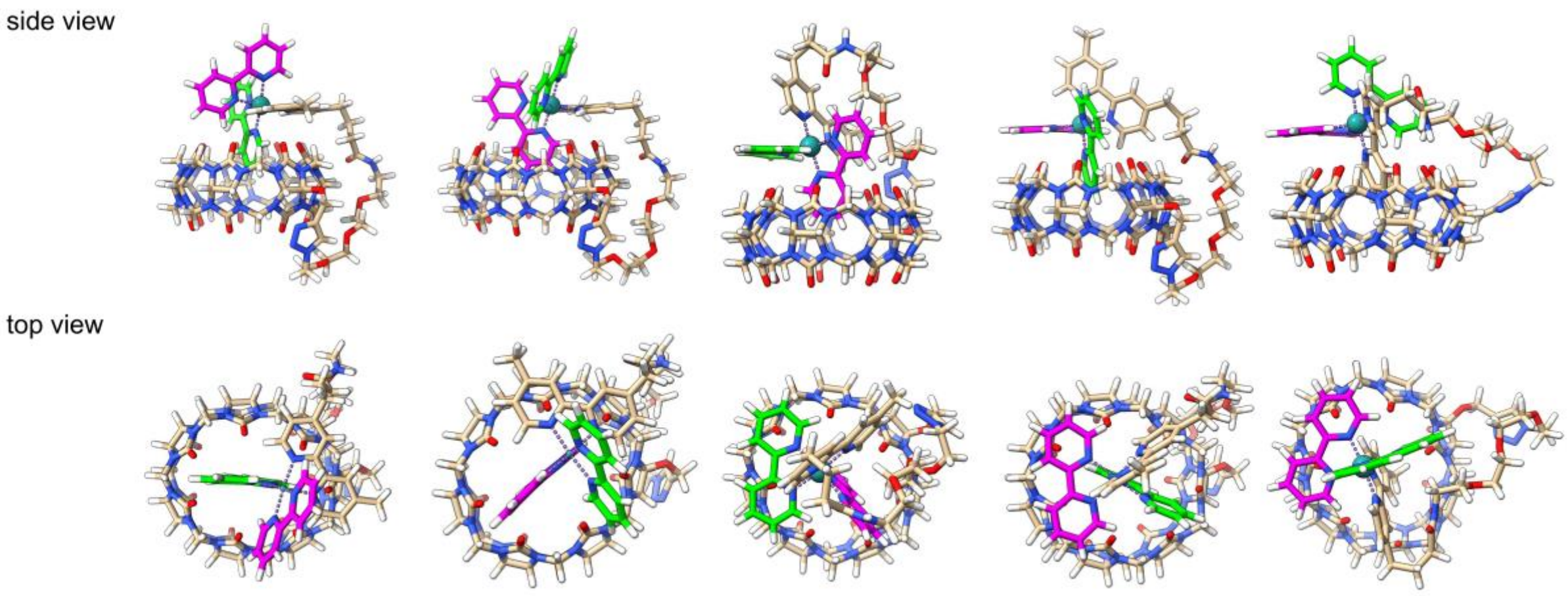

	P1	P4	P3	P2	P5
$^{TM}CCS_{N2}$ / Å^2	454.5	458.5	445.3	451.7	448.4
ΔE(GFN2-xTB) / eV	0	0.14	0.16	0.22	0.27
ΔE(r^{2}scan-3c) / eV	0	0.13	0.28	0.06	0.13

Figure S20. Energetically most favorable GFN2-xTB structure of $[CB7\text{-}Rubpy]^{2+}$ from CREST of each conformational motif (P1-P5) with their calculated collision cross section $^{TM}CCS_{N2}$, relative GFN2-xTB energy and DFT (r^{2}scan-3c) energy.

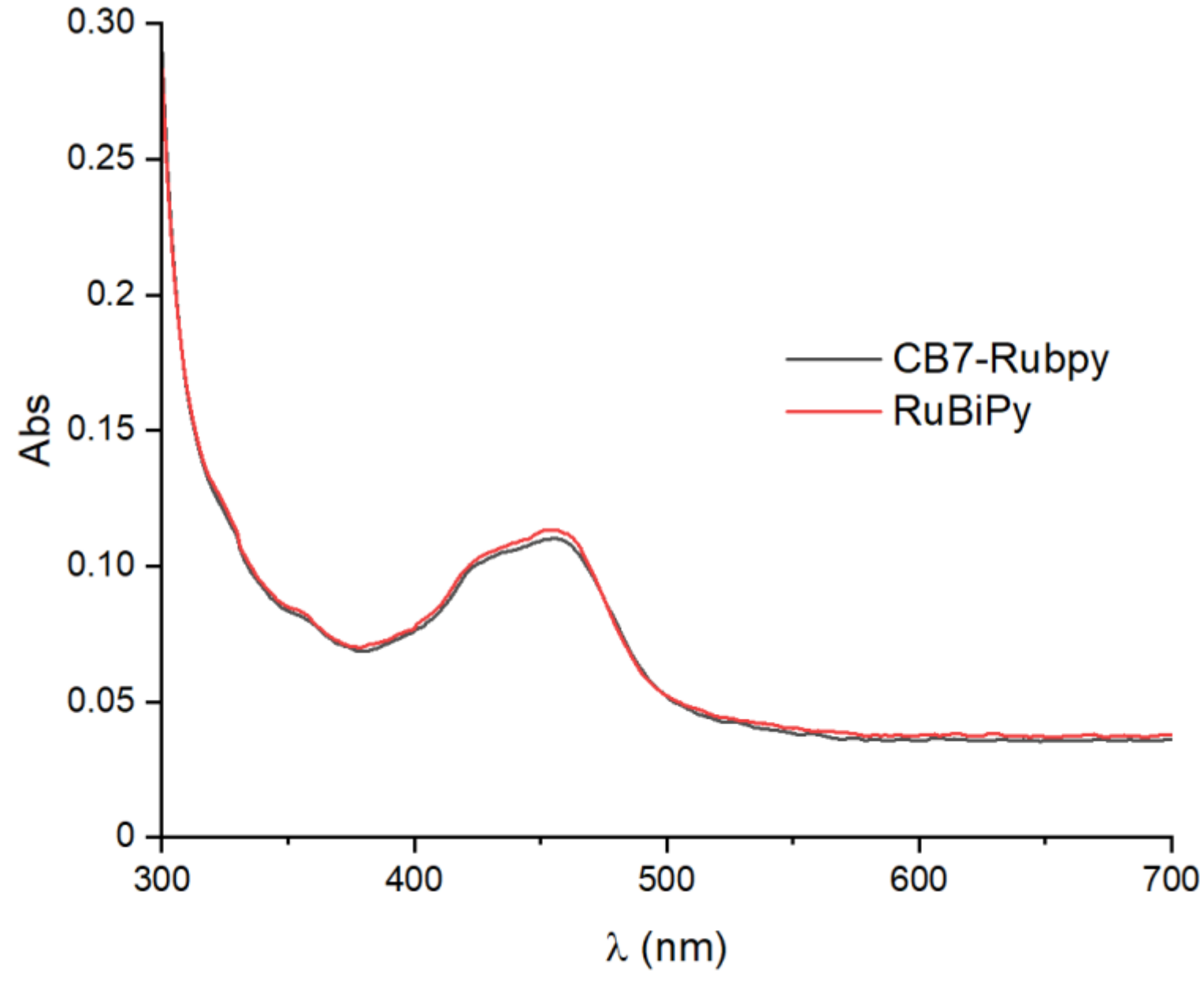

Figure S21. UV-vis absorption spectra of RuBiPy and CB7-Rubpy (5 μM) in water. Spectra were recorded using a 1 cm path length cuvette.

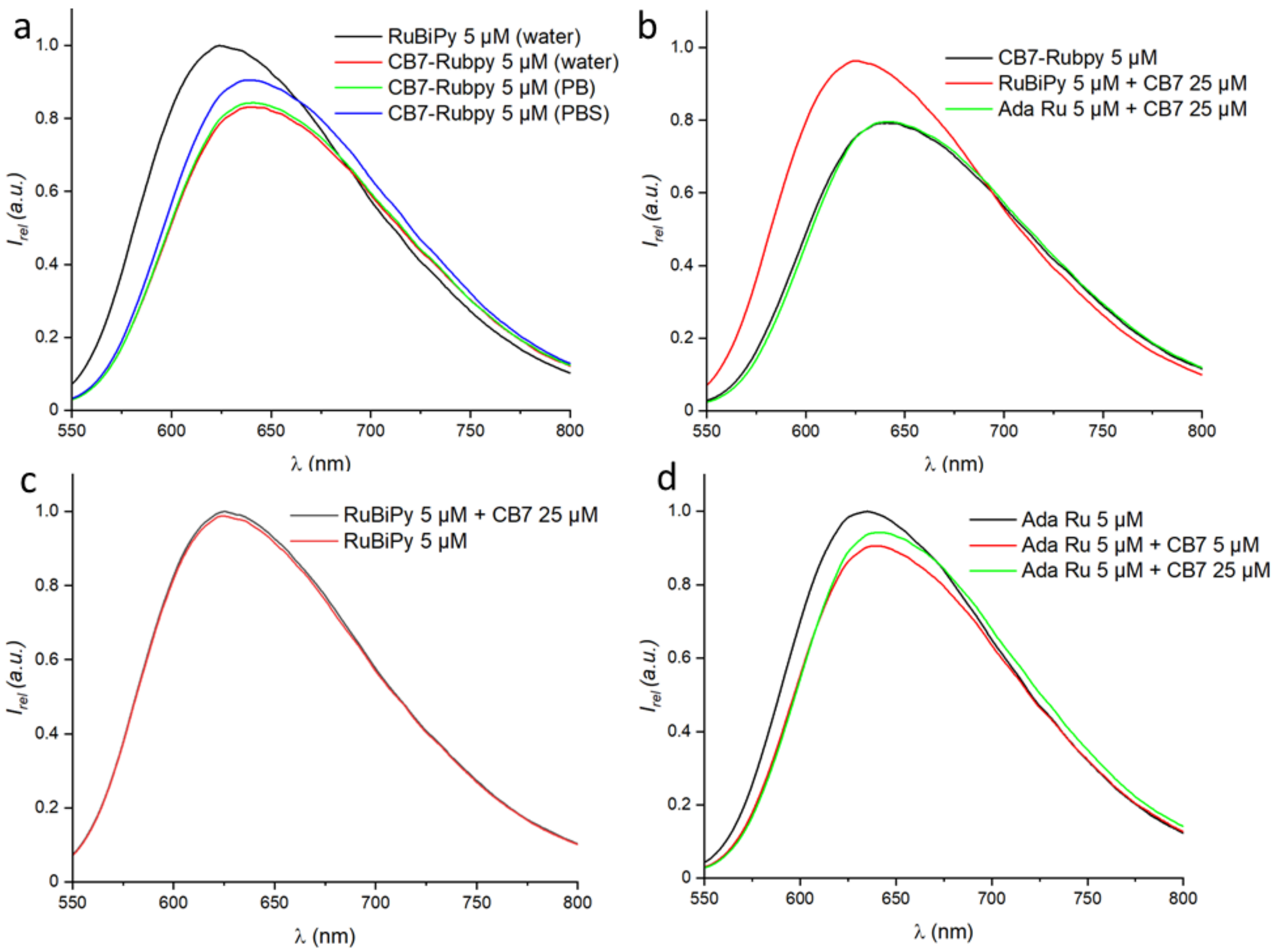

Figure S22. (a) Comparison of the emission spectra of RuBiPy (5 µM in water) and CB7-Rubpy (5 µM) in water, phosphate buffer (PB, 10 mM, pH 7.4), and phosphate-buffered saline (PBS, 1×, pH 7.4). (b) Emission response of CB7-Rubpy (5 µM, in water) compared to non-covalent reference systems, including RuBiPy (5 µM, in water) and Ada-Ru (5 µM, in water) in the presence of 5 equivalents of CB7. (c) Comparison of the emission response of RuBiPy (5 µM, in water) alone and in the presence of 5 equivalents of CB7. (d) Comparison of the emission response of Ada-Ru (5 µM, in water) alone and in the presence of 1 or 5 equivalents of CB7.

All emission spectra were recorded with λ_{exc} = 452 nm, using an excitation slit width of 5 nm and an emission slit width of 2 nm, with an emission cut-off filter at 500 nm. Spectra were normalized to the maximum emission intensity of the highest-responding sample.

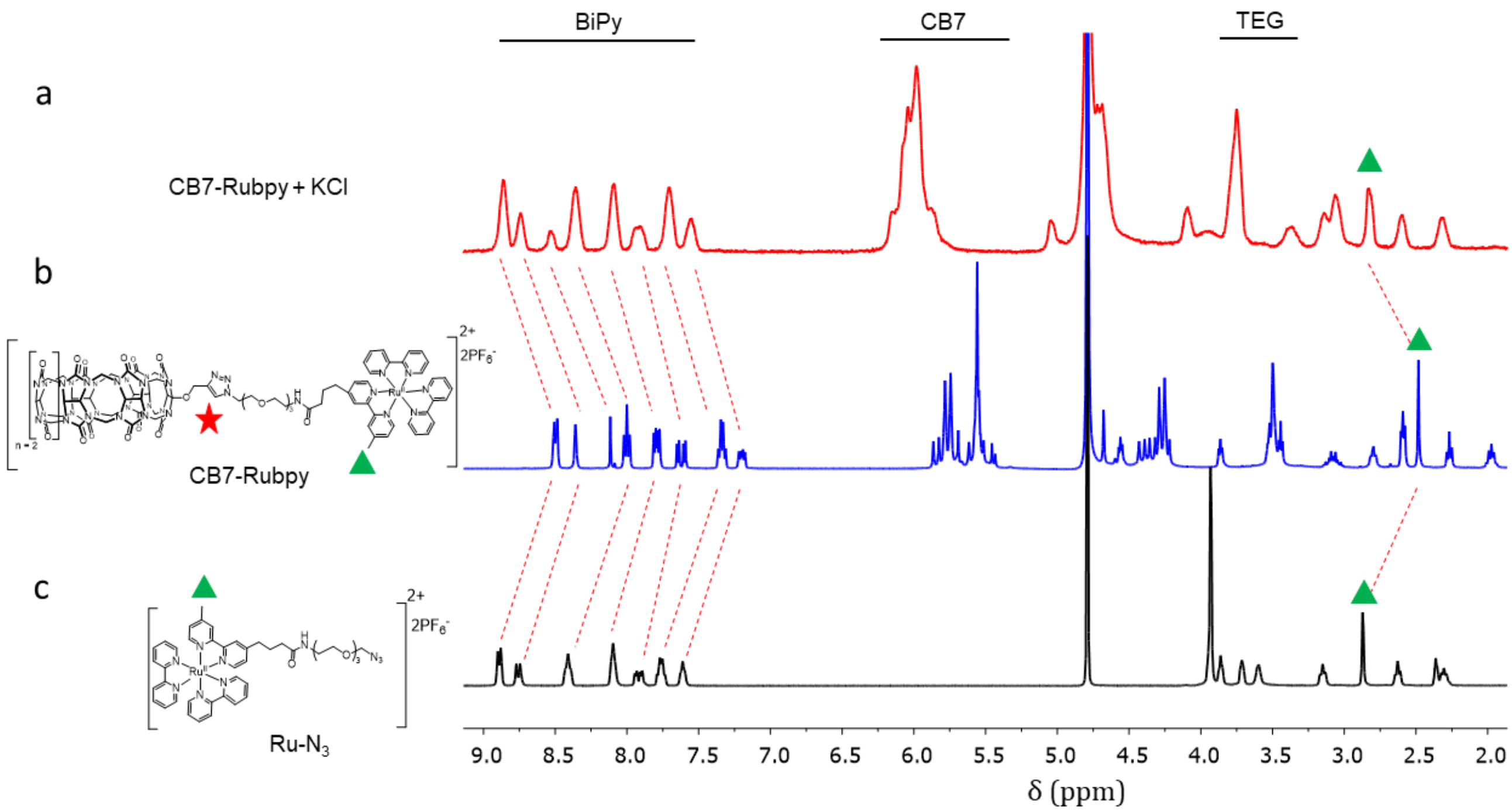

Figure S23. Overlay of ^{1}H NMR spectra (400 MHz, D_2O) of (a) CB7-Rubpy in the presence of KCl ([CB7-Rubpy] = 2 mM, [KCl] = 1000 mM), (b) CB7-Rubpy alone (2 mM), and (c) the azide-functionalized Ru(II) precursor (Ru-N_3, 2 mM). The methyl group of the linker-adjacent bipyridine ligand is marked with a green triangle, while the triazole proton is indicated by a red star.

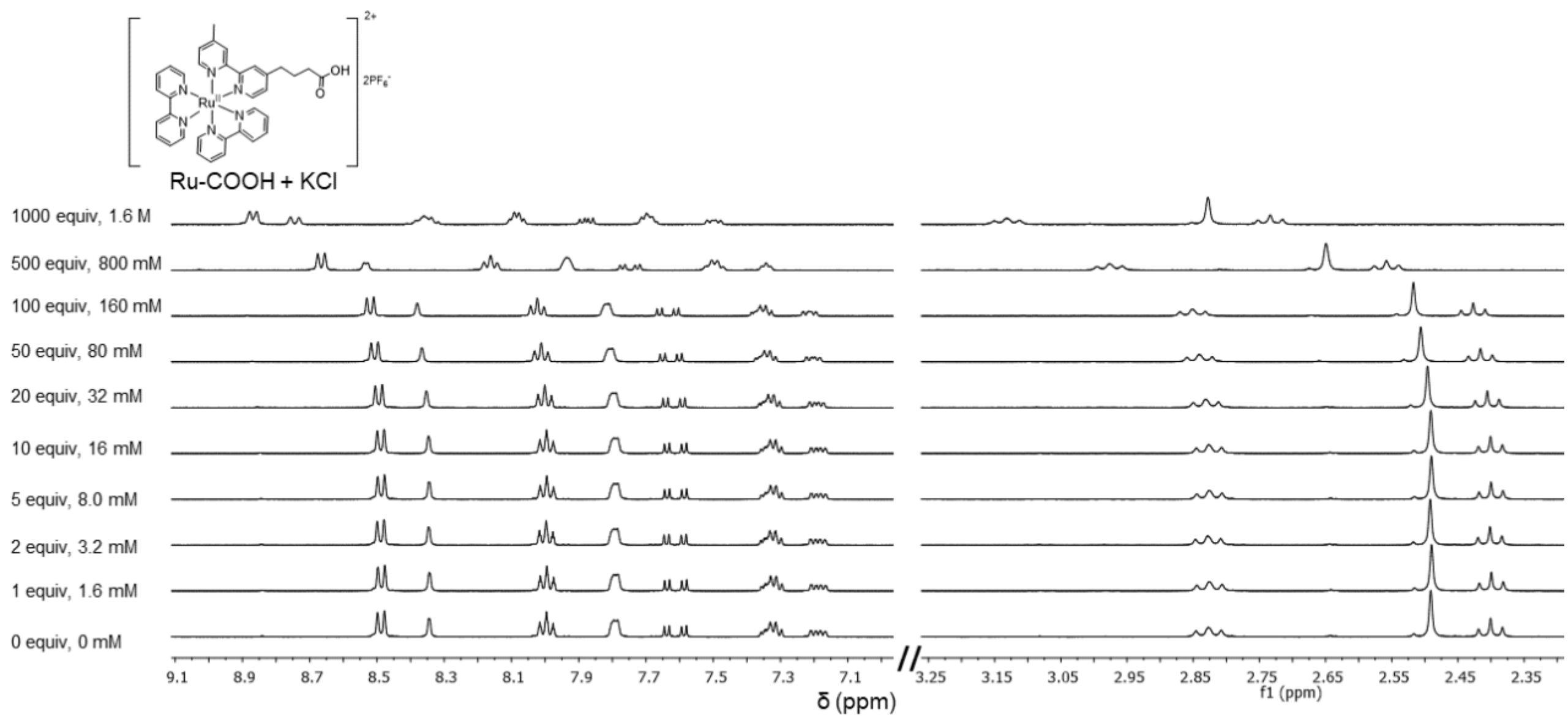

Figure S24. ^{1}H NMR titration of Ru-COOH with KCl in D_2O. Spectra were recorded at a concentration of [Ru-COOH] = 1.6 mM with increasing amounts of KCl (0-1000 equiv; corresponding to 0-1.6 M KCl). Shown are selected spectral regions highlighting (left) the aromatic bipyridine resonances and (right) the methyl signal. Both spectral regions exhibit a uniform and monotonic response upon increasing salt concentration. No significant changes are observed up to 50 equiv KCl (≤80 mM). At higher concentrations (≥50 equiv), all signals shift upfield, with the methyl resonance showing a slightly more pronounced shift compared to the aromatic signals. In contrast to CB7-Rubpy, no non-monotonic behavior is observed, indicating a uniform response of the $Ru(bpy)_3^{2+}$ unit to increasing ionic strength in the absence of the CB7 host.

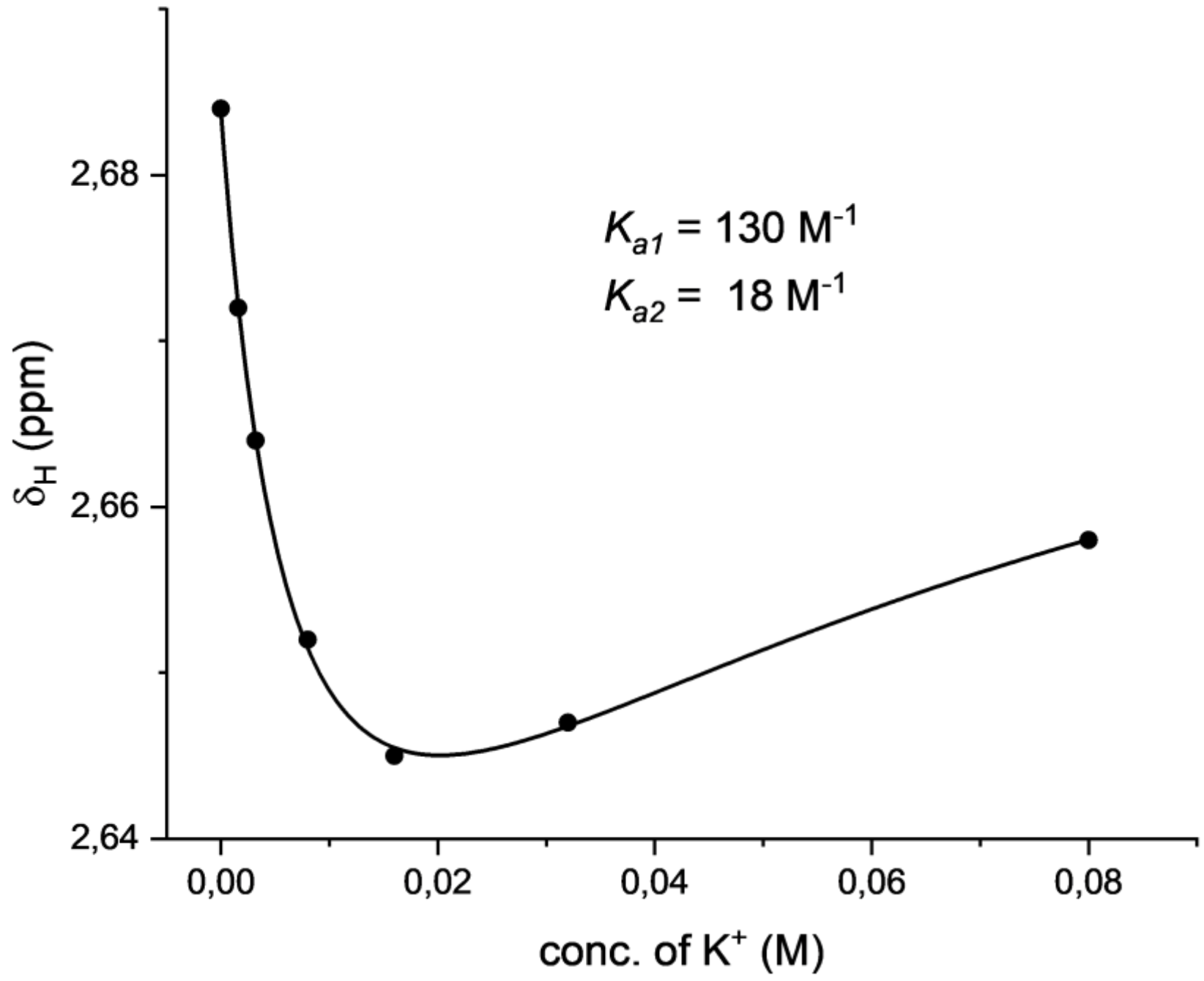

Figure S25. ^{1}H NMR titration of CB7-Rubpy with KCl. Spectra were recorded in D_2O at 400 MHz with a CB7-Rubpy concentration of 1.6 mM. The plot shows the chemical shift changes of the methyl group of the linker-adjacent bipyridine ligand upon addition of increasing concentrations of KCl. A characteristic non-monotonic shift is observed, reflecting a stepwise, salt-dependent conformational reorganization of the CB7-Rubpy system. Fitting of the titration data to a 2:1 binding model enables extraction of two binding constants (K_{a1} and K_{a2}), corresponding to the two distinct interaction regimes.

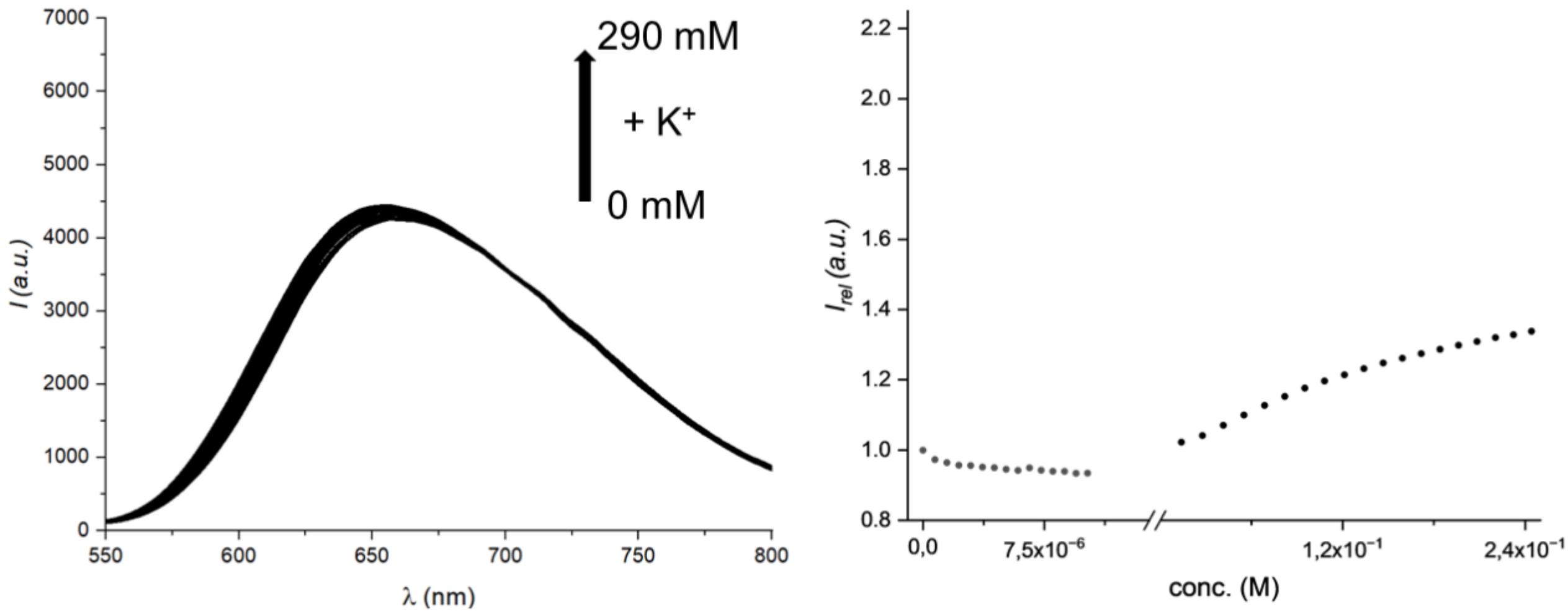

Figure S26. K^+-dependent modulation of CB7-Rubpy emission in water. Left: Emission spectra (λ_{exc} = 452 nm) of CB7-Rubpy ([CB7-Rubpy] = 1 µM) upon addition of K^+ (0-290 mM). Spectra are normalized to the maximum emission intensity of the highest-response sample. Right: Corresponding titration plot showing the emission intensity at 610 nm as a function of K^+ concentration, combining low- and high-concentration regimes. Data are normalized to the initial emission intensity of CB7-Rubpy (1 µM) in the absence of K^+.

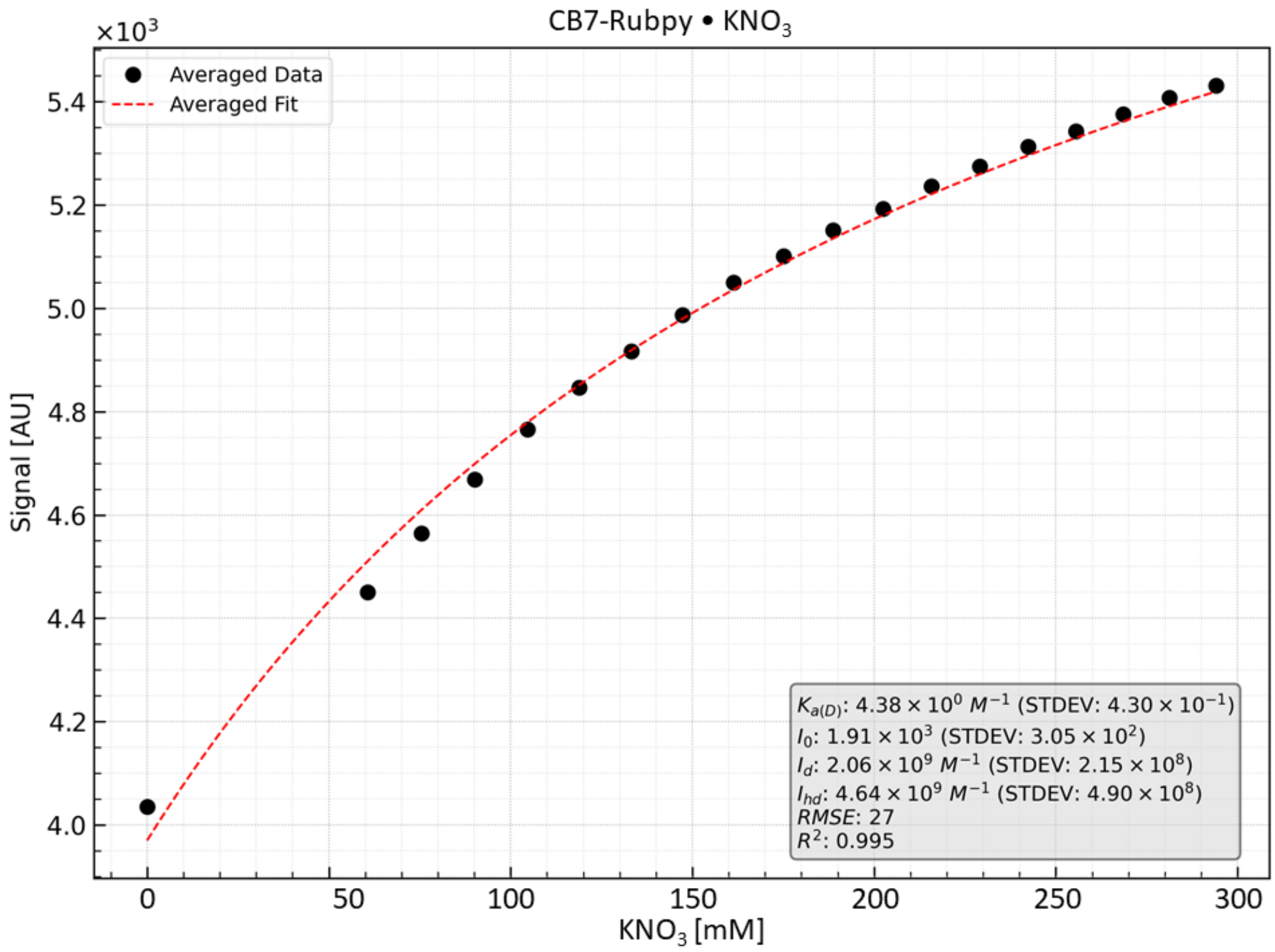

Figure S27. Titration curve and fit (1:1 binding model) of KNO_3 with CB7-Rubpy in water. Emission intensity at 610 nm (λ_{exc} = 452 nm) is plotted as a function of KNO_3 concentration ([CB7-Rubpy] = 1 µM; [KNO_3] = 0-290 mM). The solid line represents the fit to a 1:1 binding model. The extracted binding affinity corresponds to K_{a2}, representing the second, weaker binding event associated with disruption of the portal-associated CB7-Rubpy interaction and transition to the solvent-exposed (open) chromophore state.

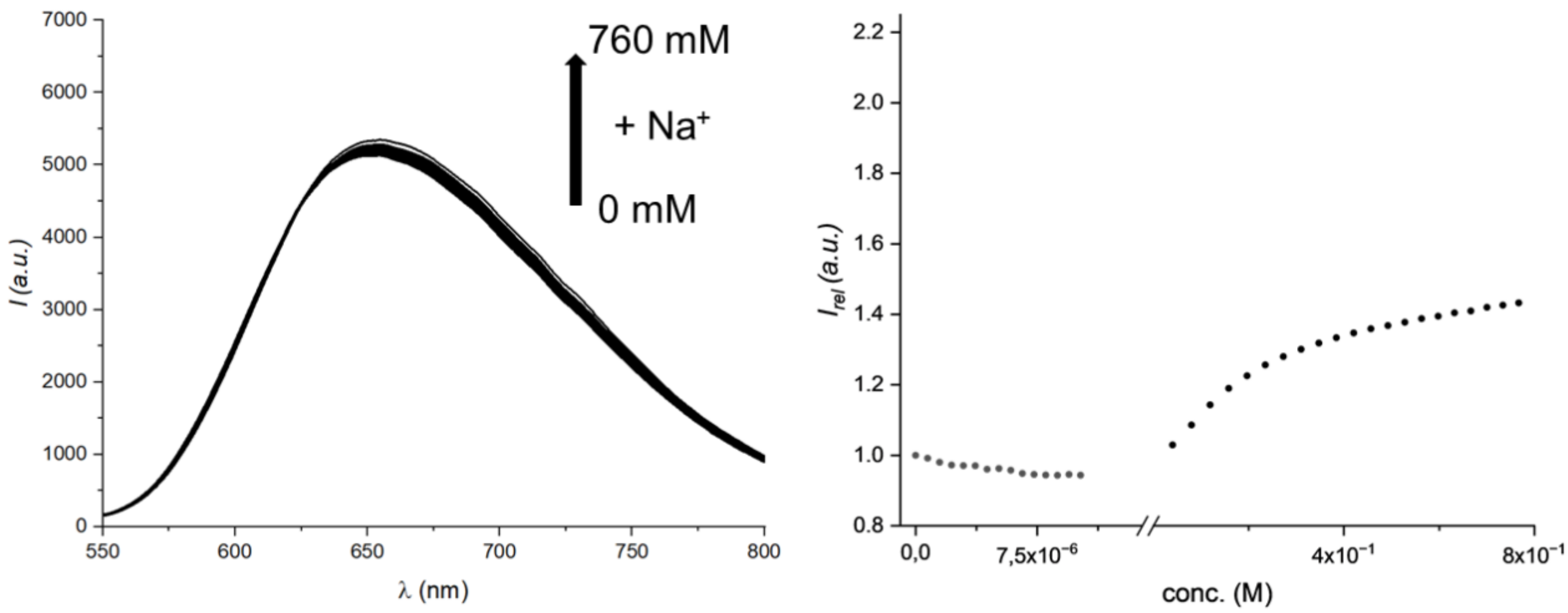

Figure S28. Na^+-dependent modulation of CB7-Rubpy emission in water. Left: Emission spectra (λ_{exc} = 452 nm) of CB7-Rubpy ([CB7-Rubpy] = 1 µM) upon addition of Na^+ (0-760 mM). Spectra are normalized to the maximum emission intensity of the highest-response sample. Right: Corresponding titration plot showing the emission intensity at 610 nm as a function of Na^+ concentration, combining low- and high-concentration regimes. Data are normalized to the initial emission intensity of CB7-Rubpy (1 µM) in the absence of Na^+.

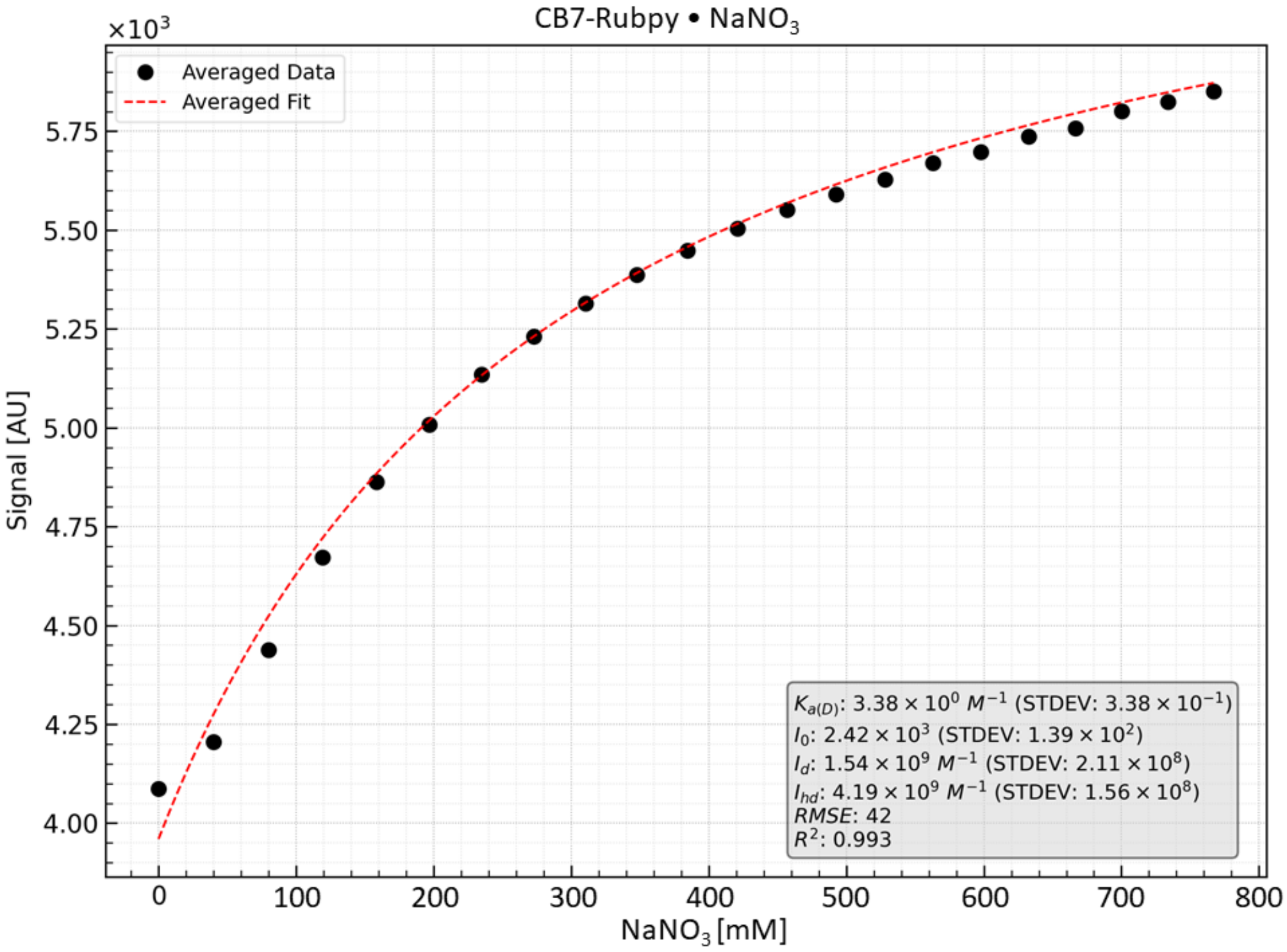

Figure S29. Titration curve and fit (1:1 binding model) of $NaNO_3$ with CB7-Rubpy in water. Emission intensity at 610 nm (λ_{exc} = 452 nm) is plotted as a function of $NaNO_3$ concentration ([CB7-Rubpy] = 1 µM; [$NaNO_3$] = 0-760 mM). The solid line represents the fit to a 1:1 binding model. The extracted binding affinity corresponds to K_{a2}, representing the second, weaker binding event associated with disruption of the portal-associated CB7-Rubpy interaction and transition to the solvent-exposed (open) chromophore state.

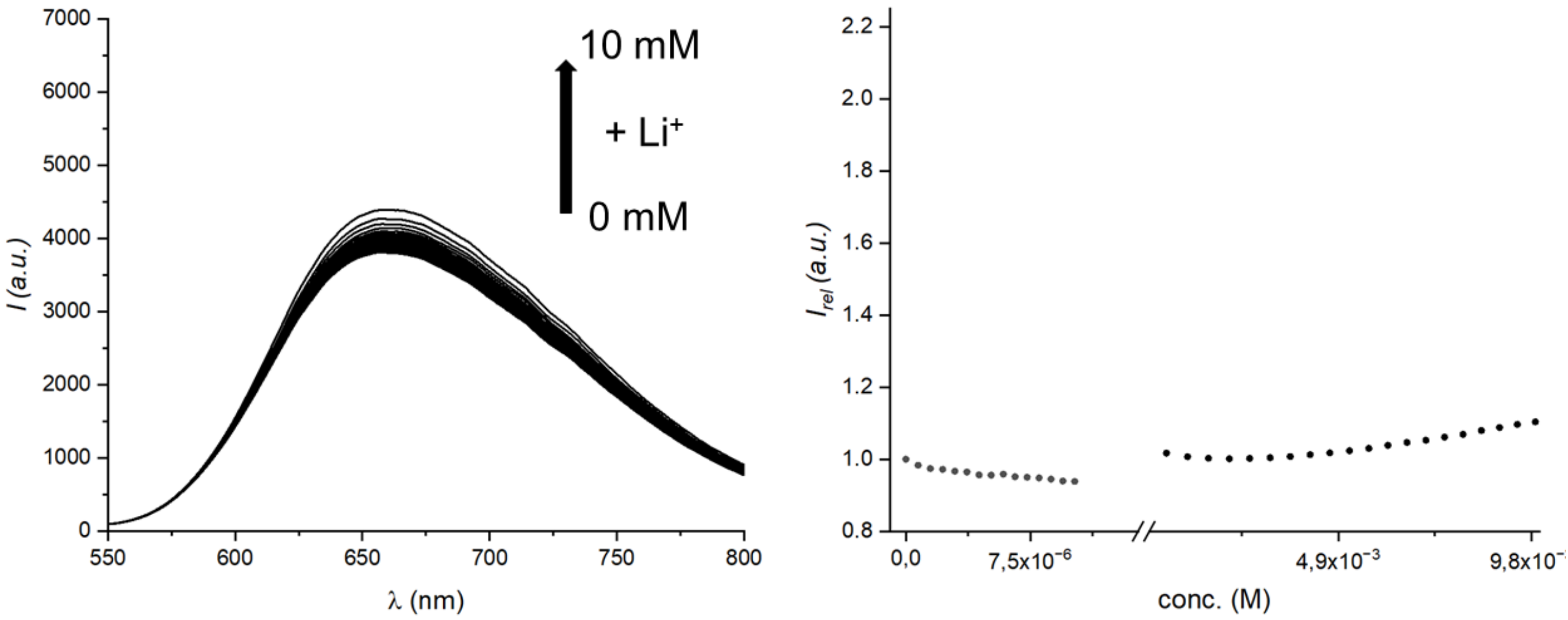

Figure S30. Li^+-dependent modulation of CB7-Rubpy emission in water. Left: Emission spectra (λ_{exc} = 452 nm) of CB7-Rubpy ([CB7-Rubpy] = 1 µM) upon addition of Li^+ (0-10 mM). Spectra are normalized to the maximum emission intensity of the highest-response sample. Right: Corresponding titration plot showing the emission intensity at 610 nm as a function of Li^+ concentration, combining low- and high-concentration regimes. Data are normalized to the initial emission intensity of CB7-Rubpy (1 µM) in the absence of Li^+.

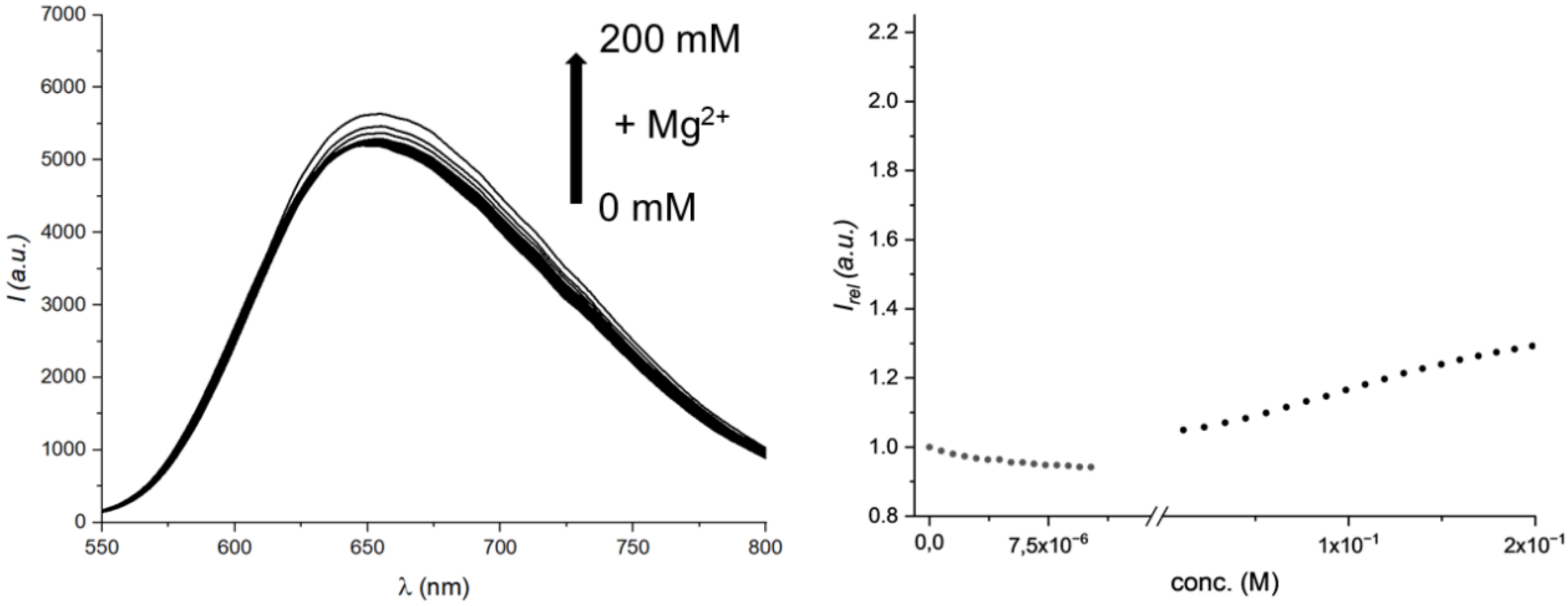

Figure S31. Mg^{2+}-dependent modulation of CB7-Rubpy emission in water. Left: Emission spectra (λ_{exc} = 452 nm) of CB7-Rubpy ([CB7-Rubpy] = 1 µM) upon addition of Mg^{2+} (0-200 mM). Spectra are normalized to the maximum emission intensity of the highest-response sample. Right: Corresponding titration plot showing the emission intensity at 610 nm as a function of Mg^{2+} concentration, combining low- and high-concentration regimes. Data are normalized to the initial emission intensity of CB7-Rubpy (1 µM) in the absence of Mg^{2+}.

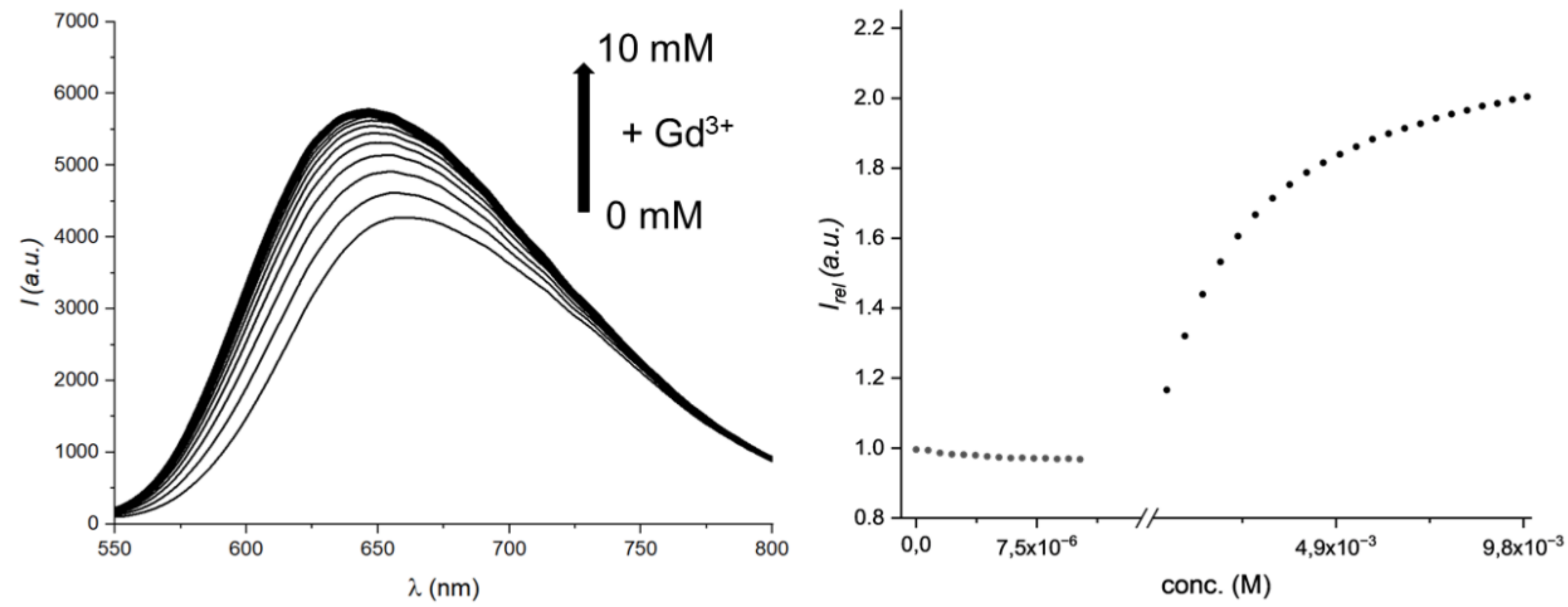

Figure S32. Gd^{3+}-dependent modulation of CB7-Rubpy emission in water. Left: Emission spectra (λ_exc = 452 nm) of CB7-Rubpy ([CB7-Rubpy] = 1 µM) upon addition of Gd^{3+} (0-10 mM). Spectra are normalized to the maximum emission intensity of the highest-response sample. Right: Corresponding titration plot showing the emission intensity at 610 nm as a function of Gd^{3+} concentration, combining low- and high-concentration regimes. Data are normalized to the initial emission intensity of CB7-Rubpy (1 µM) in the absence of Gd^{3+}.

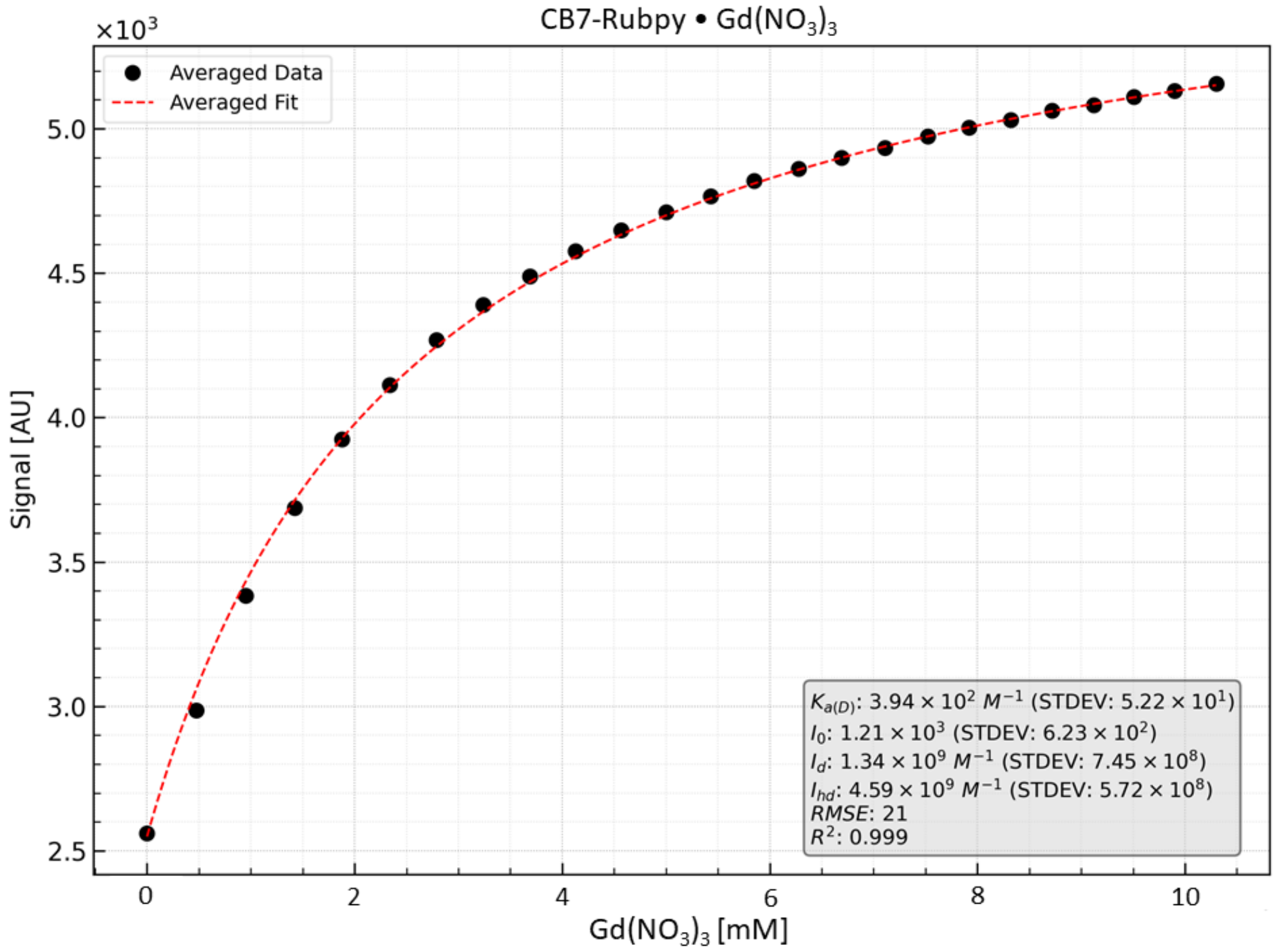

Figure S33. Titration curve and fit (1:1 binding model) of $Gd(NO_3)_3$ with CB7-Rubpy in water. Emission intensity at 610 nm (λexc = 452 nm) is plotted as a function of $Gd(NO_3)_3$ concentration ([CB7-Rubpy] = 1 µM; [$Gd(NO_3)_3$] = 0-10 mM). The solid line represents the fit to a 1:1 binding model.

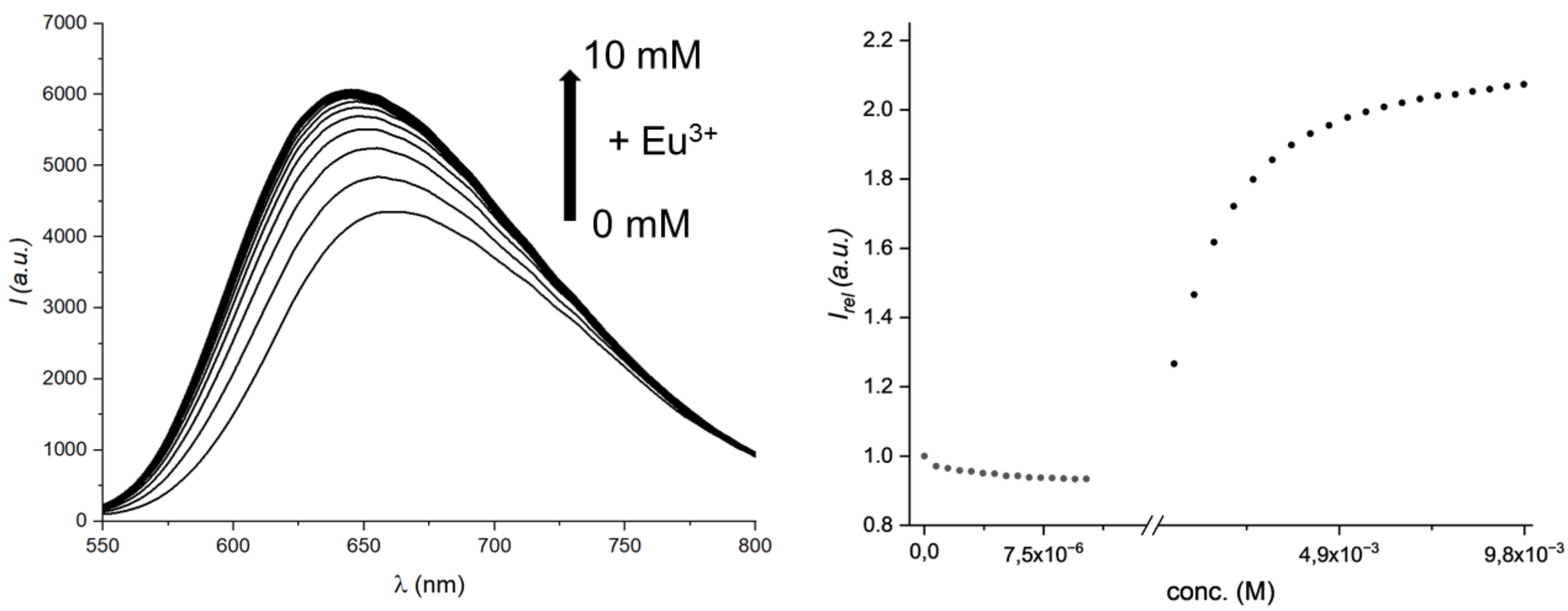

Figure S34. Eu^{3+}-dependent modulation of CB7-Rubpy emission in water. Left: Emission spectra (λ_{exc} = 452 nm) of CB7-Rubpy ([CB7-Rubpy] = 1 µM) upon addition of Eu^{3+} (0-10 mM). Spectra are normalized to the maximum emission intensity of the highest-response sample. Right: Corresponding titration plot showing the emission intensity at 610 nm as a function of Eu^{3+} concentration, combining low- and high-concentration regimes. Data are normalized to the initial emission intensity of CB7-Rubpy (1 µM) in the absence of Eu^{3+}.

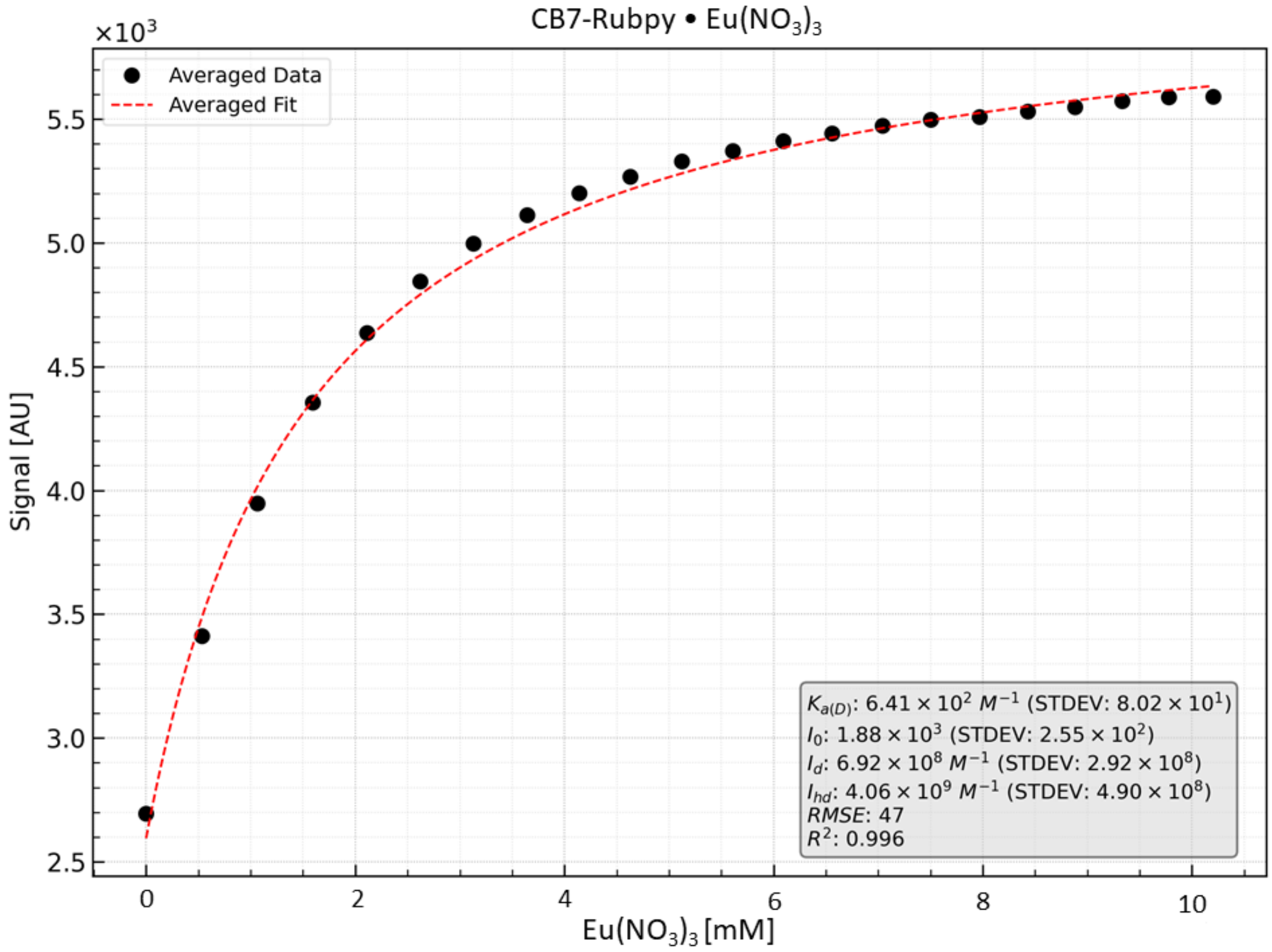

Figure S35. Titration curve and fit (1:1 binding model) of $Eu(NO_3)_3$ with CB7-Rubpy in water. The emission intensity at 610 nm (λ_{exc} = 452 nm) is plotted as a function of $Eu(NO_3)_3$ concentration ([CB7-Rubpy] = 1 µM; [$Eu(NO_3)_3$] = 0-10 mM). The solid line represents the fit to a 1:1 binding model.

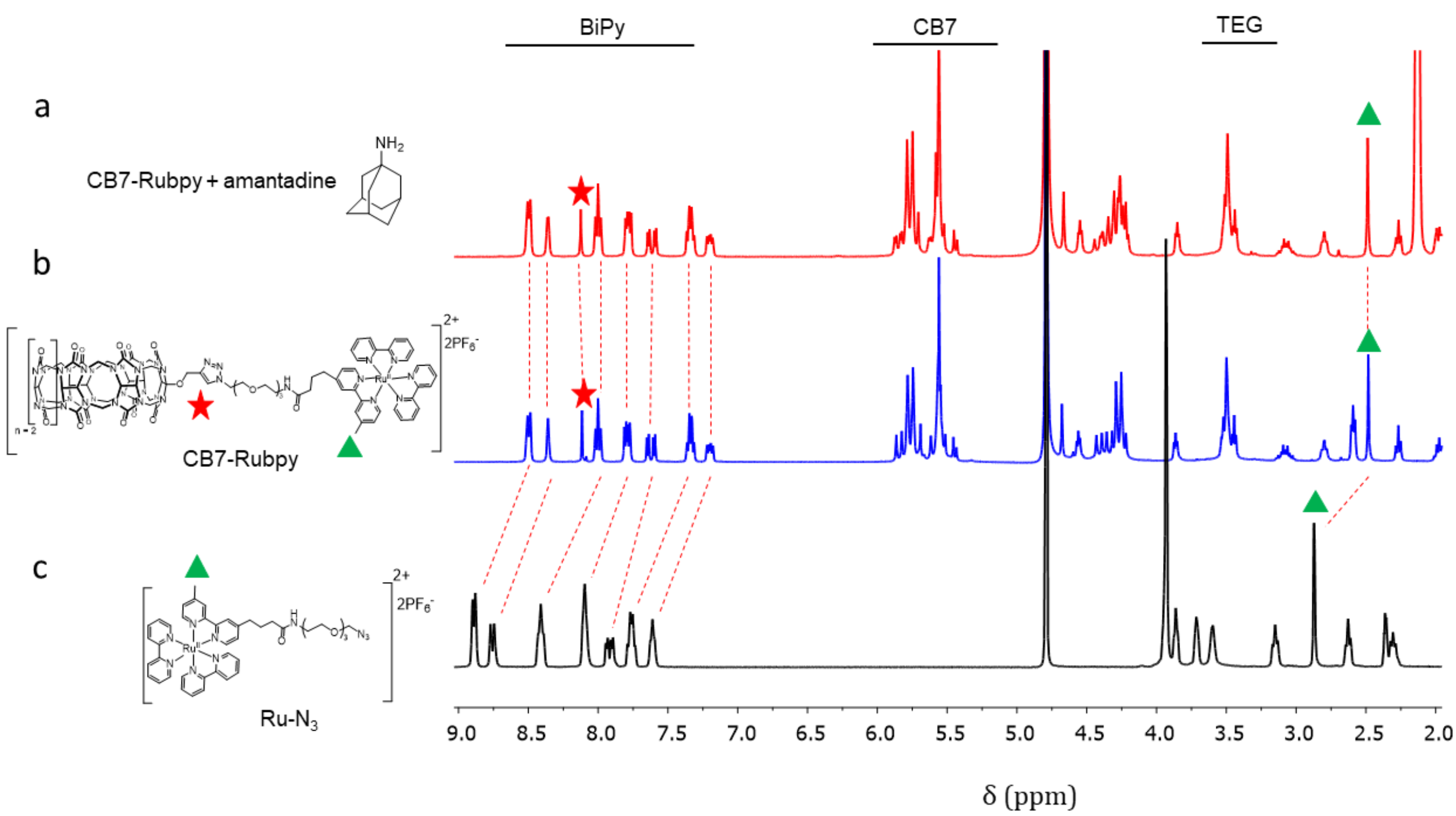

Figure S36. Overlay of ¹H NMR spectra (400 MHz, D_2O) of (a) CB7-Rubpy in the presence of amantadine ([CB7-Rubpy] = 2 mM, [amantadine] = 20 mM), (b) CB7-Rubpy alone (2 mM), and (c) the azide-functionalized Ru(II) precursor ($Ru-N_3$, 2 mM). The methyl group of the linker-adjacent bipyridine ligand is marked with a green triangle, while the triazole proton is indicated by a red star.

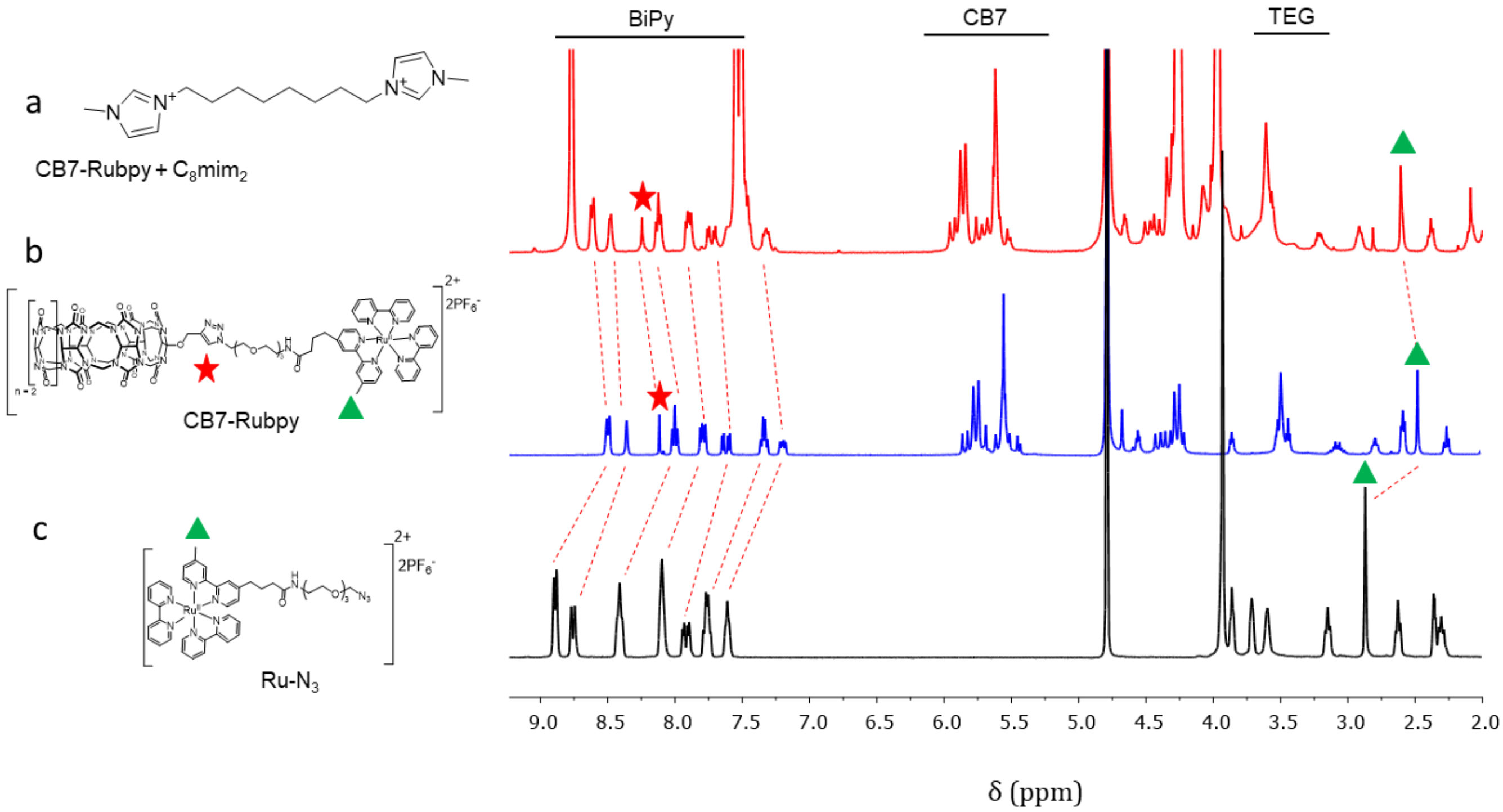

Figure S37. Overlay of ^{1}H-NMR spectra (400 MHz, in D_2O) of (a) CB7-Rubpy in the presence of $[C_8(mim)_2]^{2+}$ ([CB7-Rubpy] = 2 mM, $[C_8(mim)_2]^{2+}$ = 20 mM), (b) CB7-Rubpy alone (2 mM), and (c) the azide-functionalized Ru(II) precursor (Ru-N_3, 2 mM). The methyl group of the linker-adjacent bipyridine ligand is marked with a green triangle, while the triazole proton is indicated by a red star.

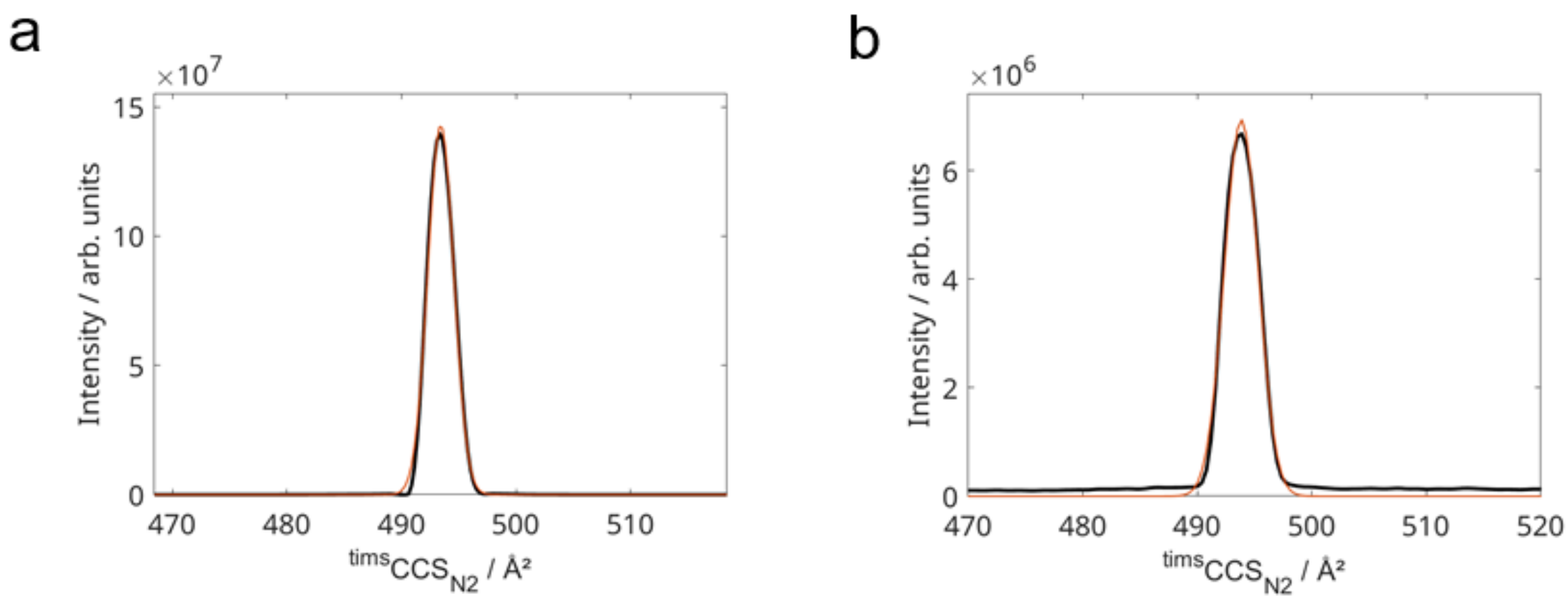

Figure S38. Ion mobility spectra of (a) $[\text{CB7-Rubpy}\bullet \text{C8(mim)}_2\text{Br}_2]^{2+}$ and (b) $[\text{CB7-Rubpy}\bullet\text{amantadine}]^{3+}$, with experimental $^{tims}CCS_{N2}$ values of 493.5 and 493.8 Å², respectively.

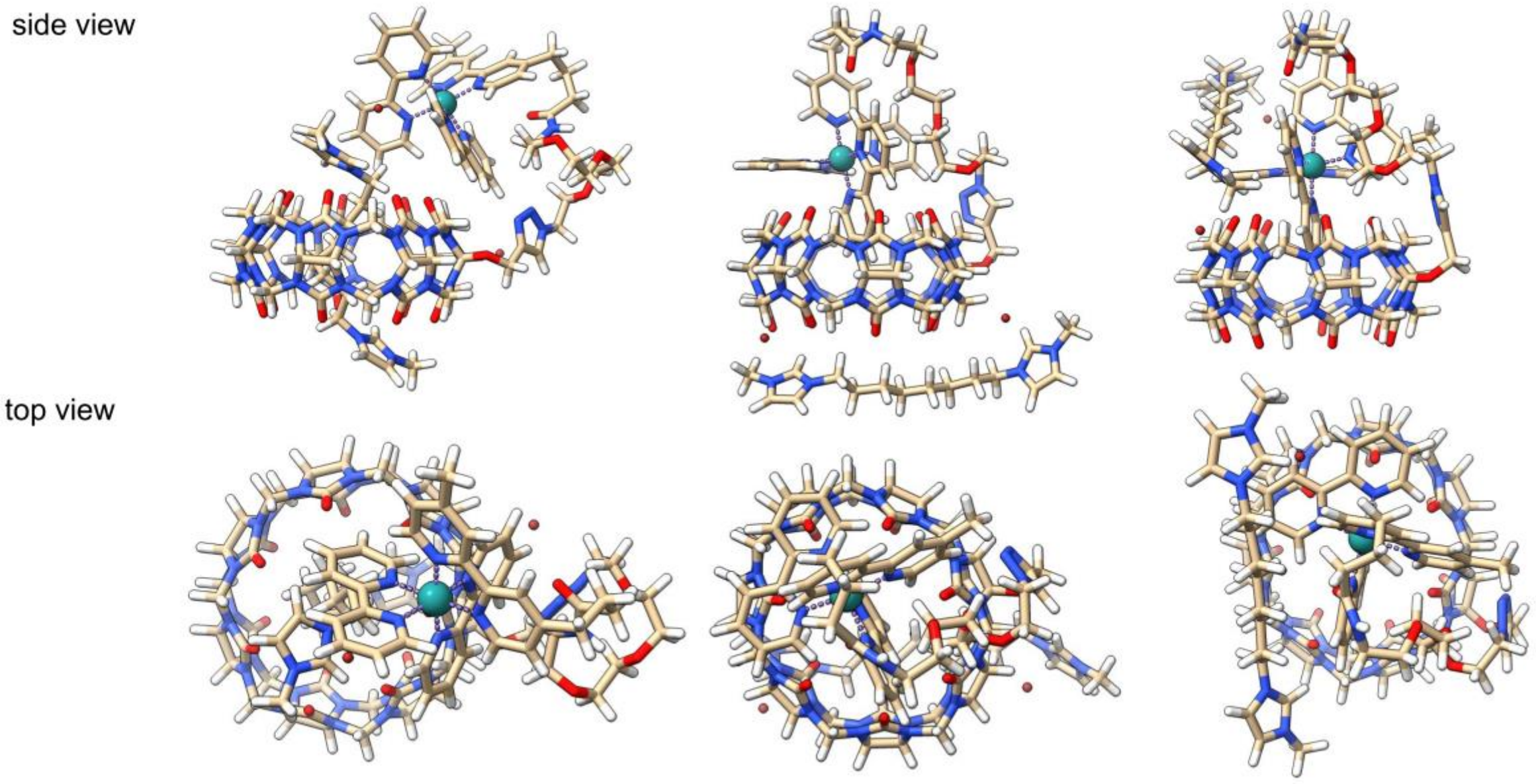

	CI1	CE1	CE2
$^{TM}CCS_{N2}$ / Å^2	501.5	506.5	499.3
ΔE(GFN2-xTB) / eV	0	0.52	1.13
ΔE(r^2scan-3c) / eV	0	0.85	1.50
ΔE(r^2scan-3c / COSMO) / eV	0	0.46	0.48

Figure S39. Representative inclusion and exclusion structures of [CB7- Rubpy•$C_8(mim)_2Br_2]^{2+}$ with their calculated collision cross section $^{TM}CCS_{N2}$, relative GFN2-xTB energy and DFT energies in gas-phase (r^2scan-3c) and in solution (r^2scan-3c / COSMO).

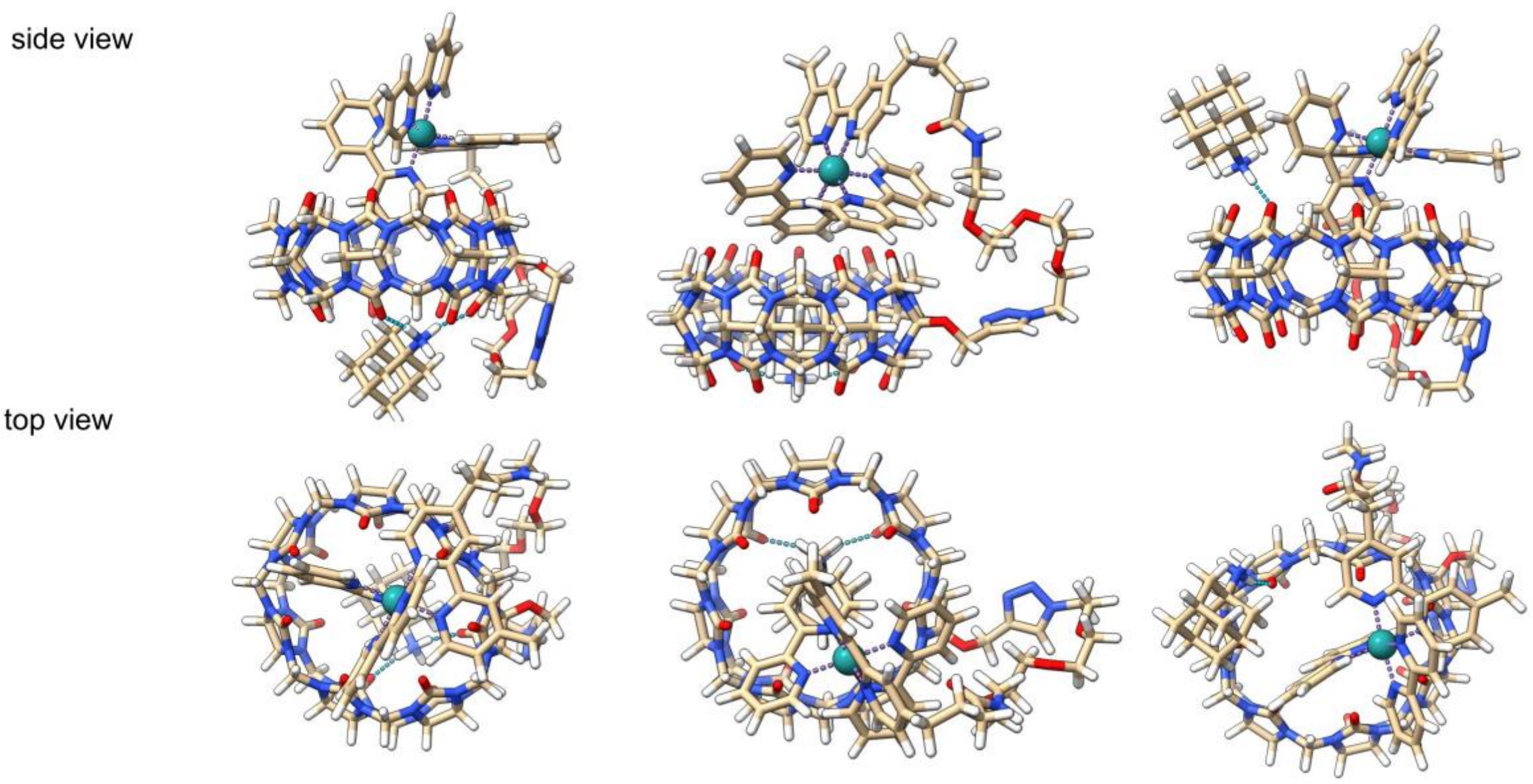

	AE1	AI1	AE2
$^{TM}CCS_{N2}$ / Å^2	483.0	497.4	506.9
ΔE(GFN2-xTB) / eV	0	0.42	2.12
ΔE(r^2scan-3c) / eV	0	0.28	1.95
ΔE(r^2scan-3c / COSMO) / eV	0.8	0	1.12

Figure S40. Representative inclusion and exclusion structures of [CB7-Rubpy•amantadine]$^{3+}$ with their calculated collision cross section $^{TM}CCS_{N2}$, relative GFN2-xTB energy and DFT energies in gas-phase (r^{2}scan-3c) and in solution (r^{2}scan-3c / COSMO).

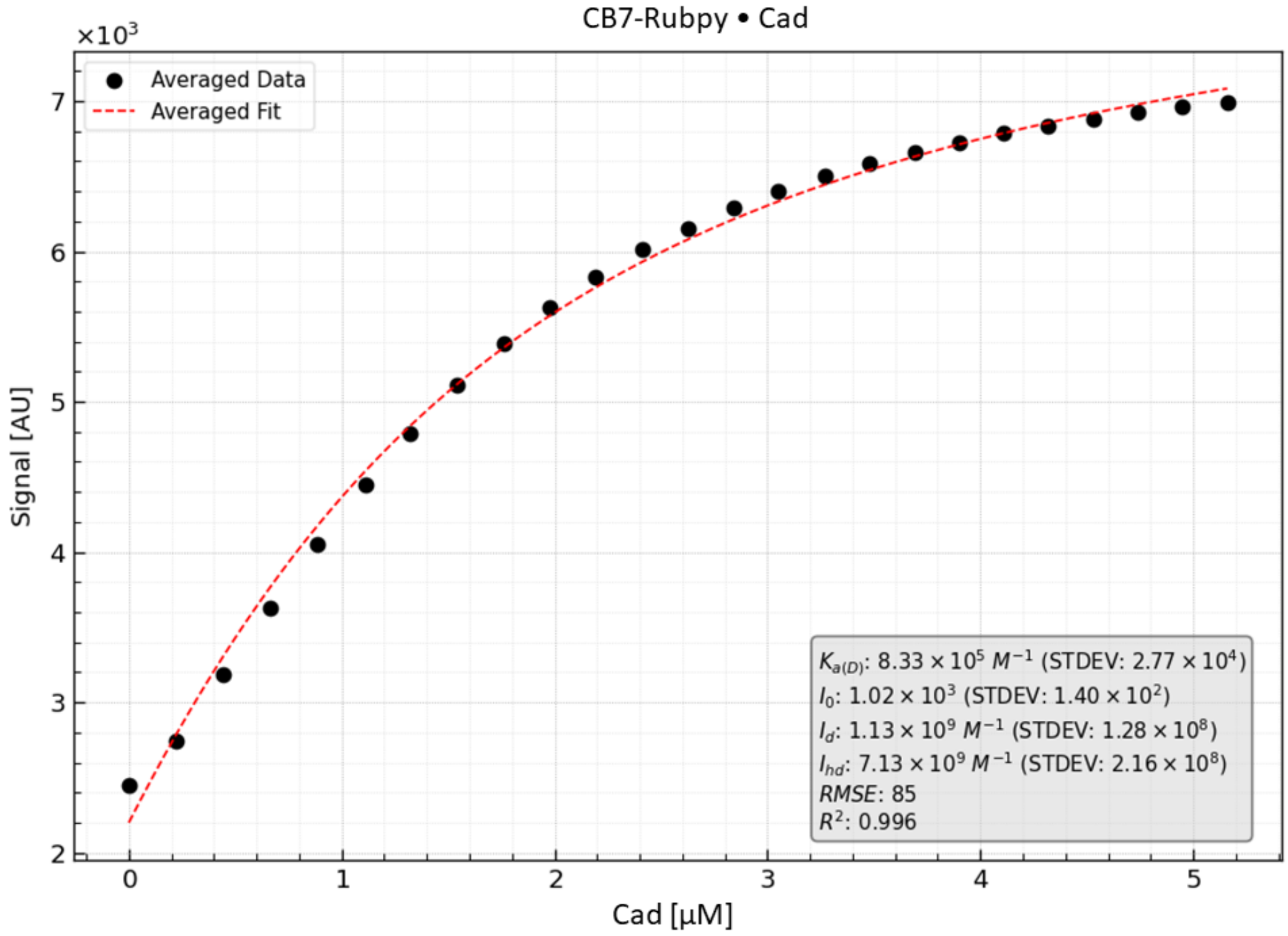

Figure S41. Titration curve and fit (1:1 binding model) of cadaverine with CB7-Rubpy in water. The emission intensity at 610 nm (λ_{exc} = 452 nm) is plotted as a function of cadaverine concentration ([CB7-Rubpy] = 1 µM; [cadaverine] = 0-5 µM). The dashed line represents the fit to a 1:1 binding model.

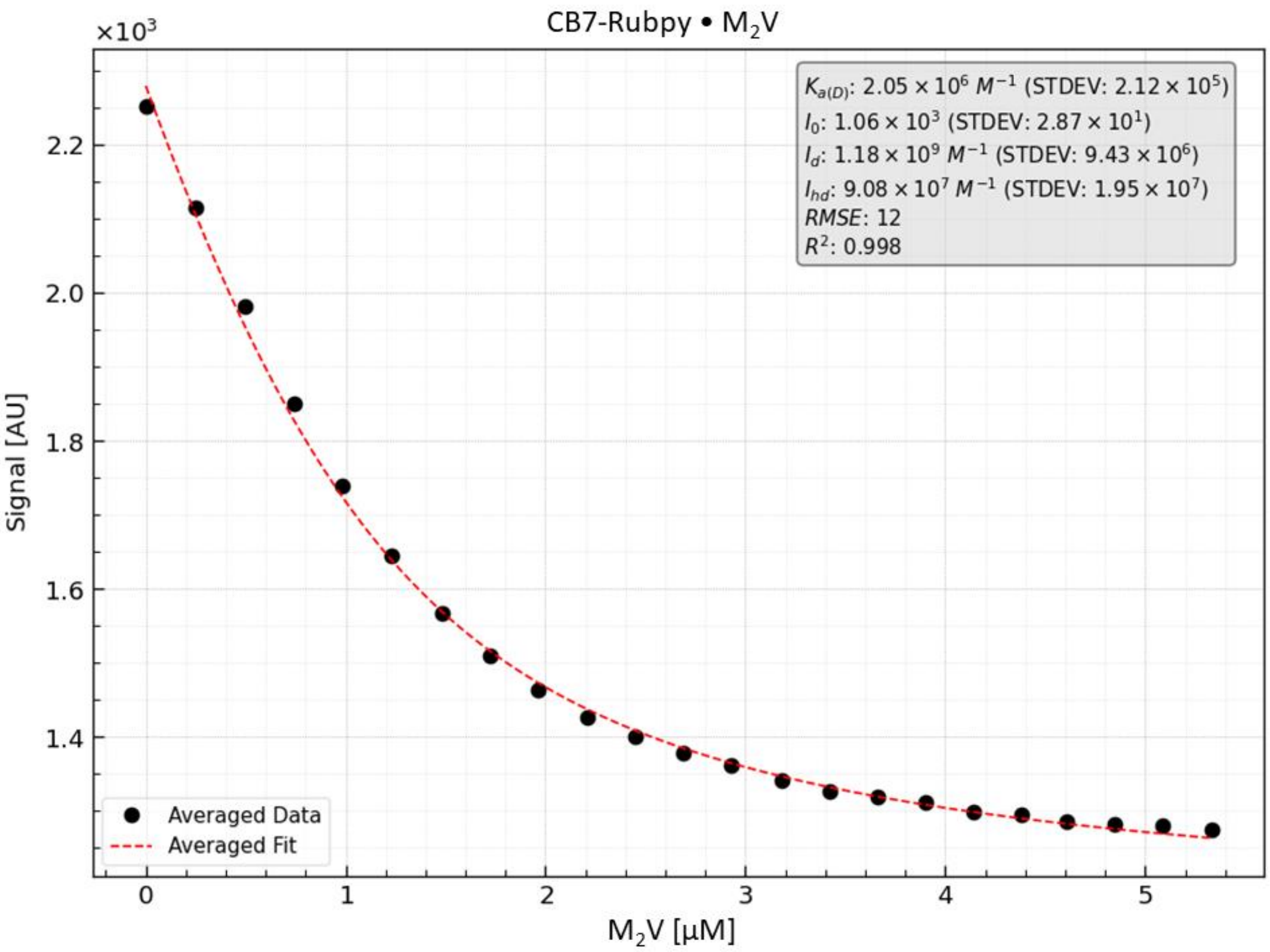

Figure S42. Titration curve and fit (1:1 binding model) of methyl viologen with CB7-Rubpy in water. The emission intensity at 610 nm (λ_{exc} = 452 nm) is plotted as a function of methyl viologen concentration ([CB7-Rubpy] = 1 µM; [methyl viologen] = 0-15 µM). The dashed line represents the fit to a 1:1 binding model.

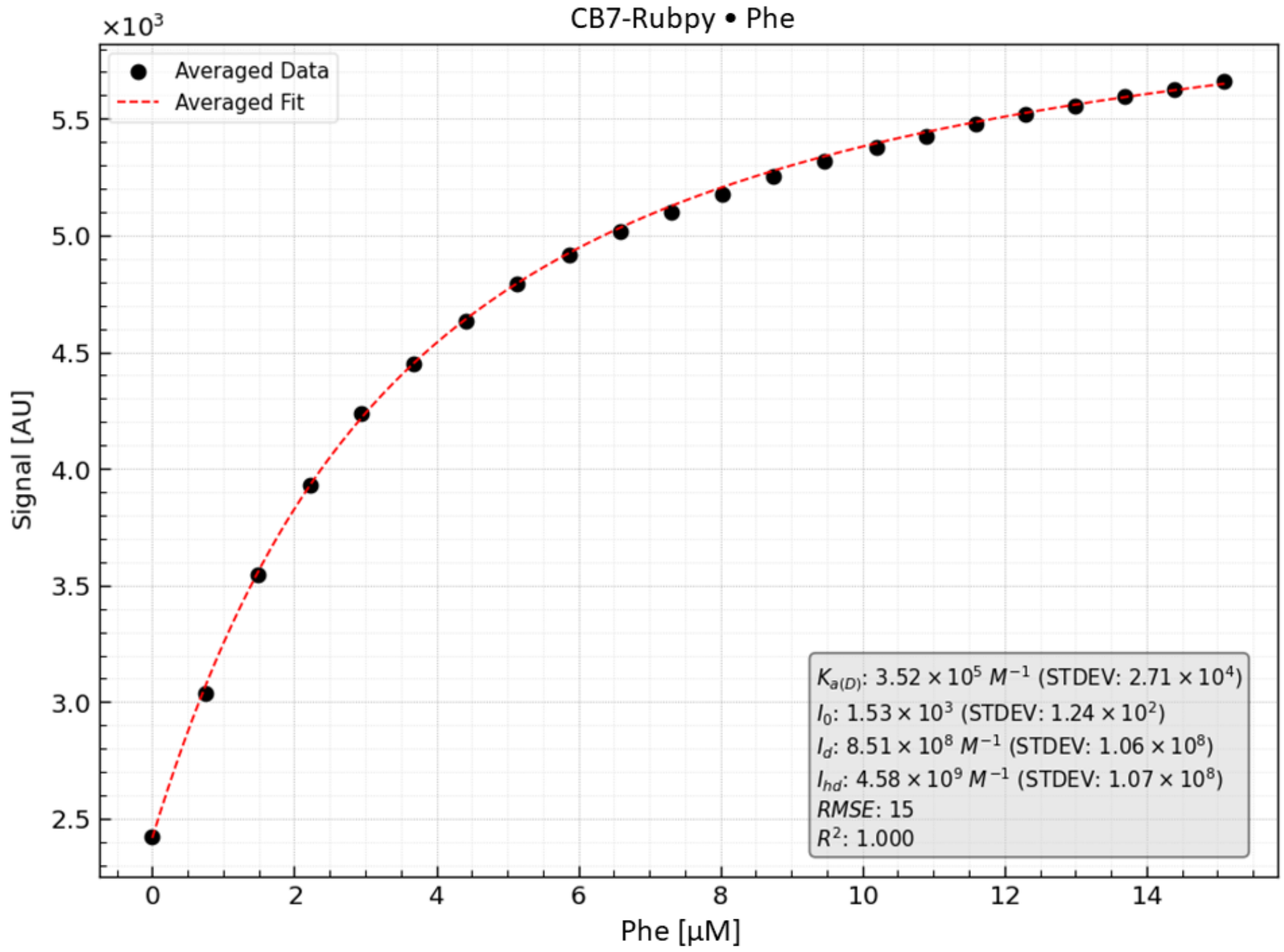

Figure S43. Titration curve and fit (1:1 binding model) of phenylalanine with CB7-Rubpy in water. The emission intensity at 610 nm (λ_{exc} = 452 nm) is plotted as a function of phenylalanine concentration ([CB7-Rubpy] = 1 µM; [phenylalanine] = 0-15 µM). The dashed line represents the fit to a 1:1 binding model.

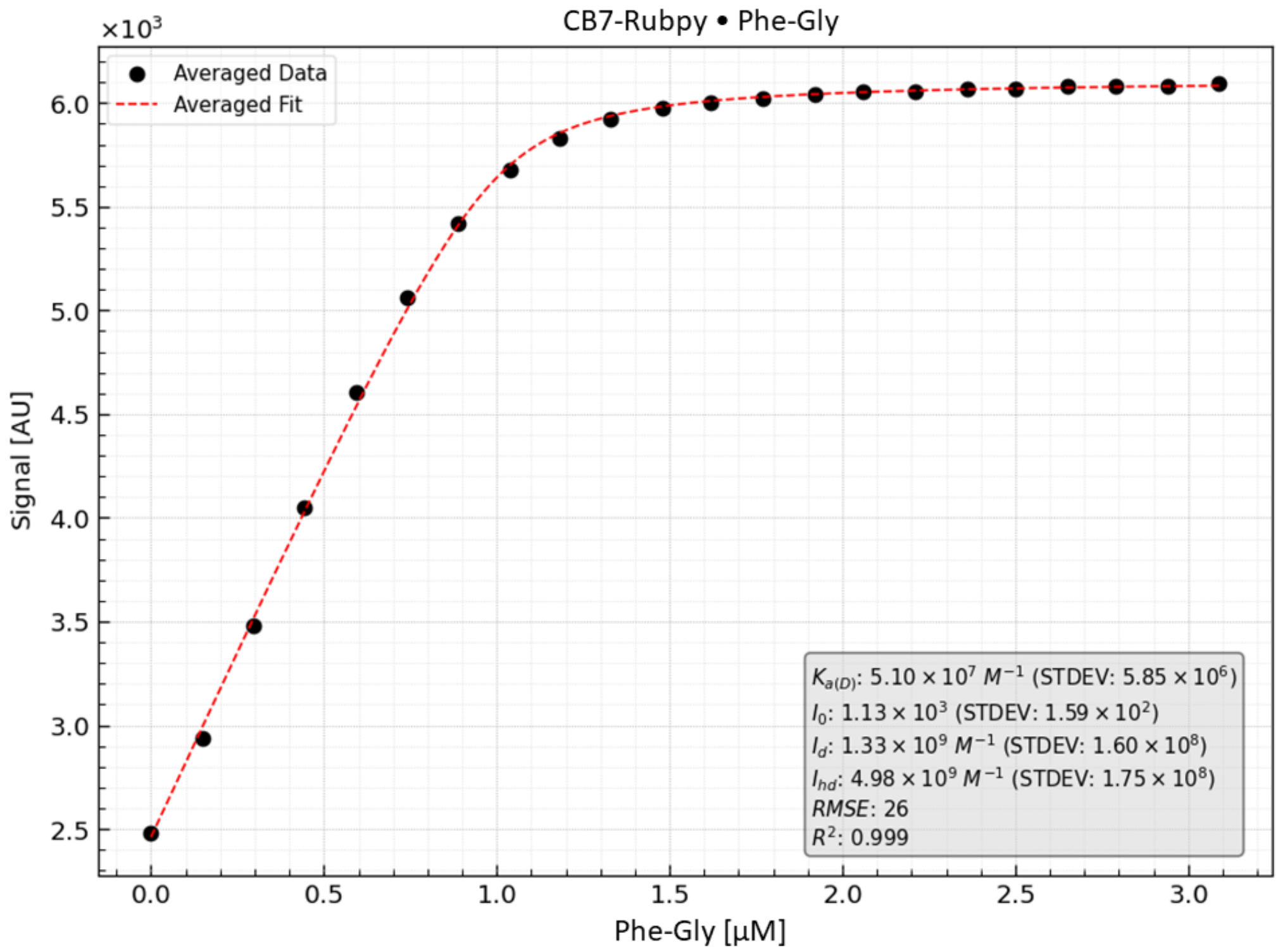

Figure S44. Titration curve and fit (1:1 binding model) of phenylalanyl-glycine (Phe-Gly) with CB7-Rubpy in water. The emission intensity at 610 nm (λ_{exc} = 452 nm) is plotted as a function of Phe-Gly concentration ([CB7-Rubpy] = 1 µM; [Phe-Gly] = 0-3 µM). The dashed line represents the fit to a 1:1 binding model.

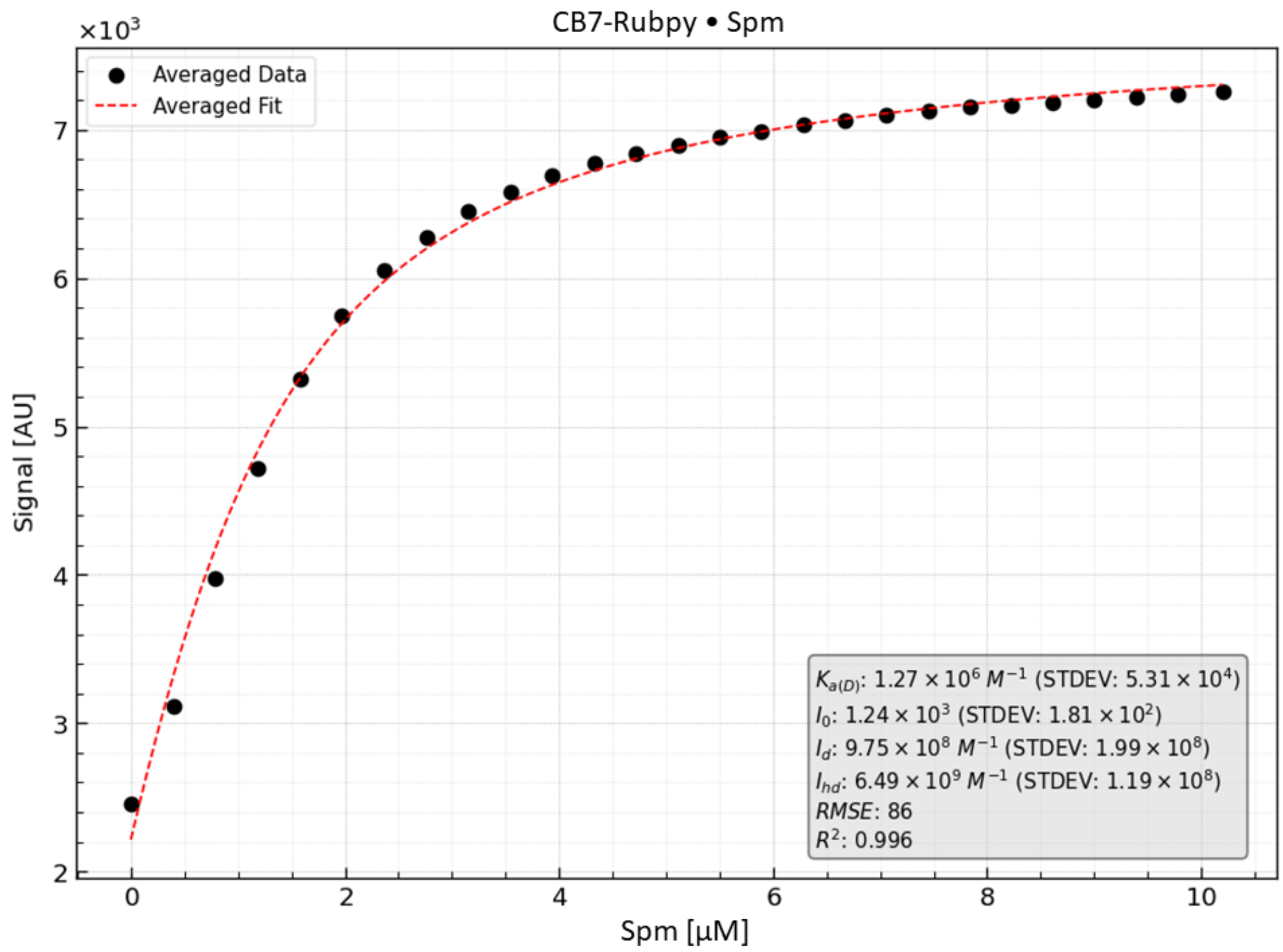

Figure S45. Titration curve and fit (1:1 binding model) of spermine (Spm) with CB7-Rubpy in water. The emission intensity at 610 nm (λ_{exc} = 452 nm) is plotted as a function of spermine concentration ([CB7-Rubpy] = 1 µM; [Spm] = 0-10 µM). The dashed line represents the fit to a 1:1 binding model.

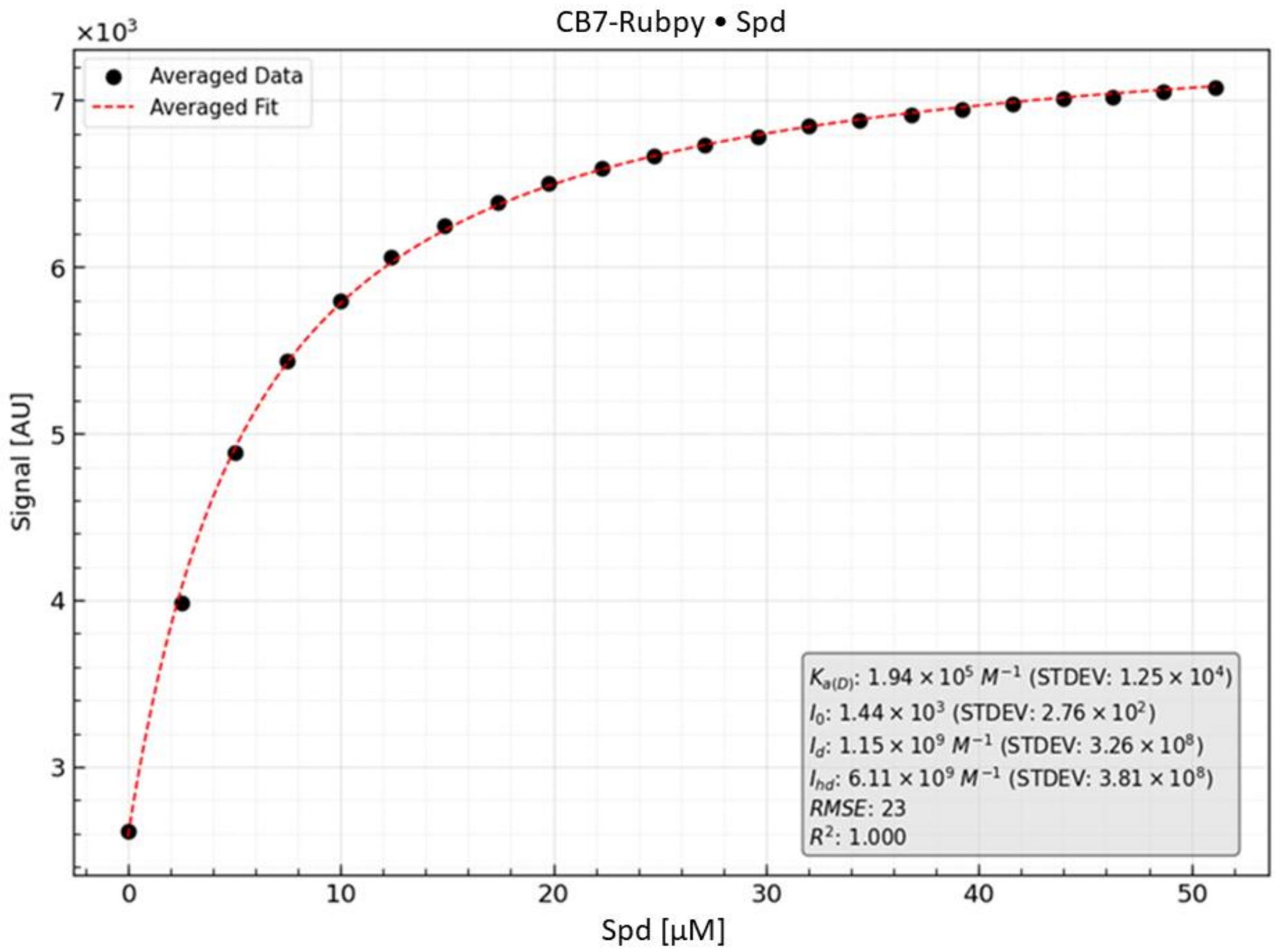

Figure S46. Titration curve and fit (1:1 binding model) of spermidine (Spd) with CB7-Rubpy in water. The emission intensity at 610 nm (λ_{exc} = 452 nm) is plotted as a function of spermidine concentration ([CB7-Rubpy] = 1 µM; [Spd] = 0-50 µM). The dashed line represents the fit to a 1:1 binding model.

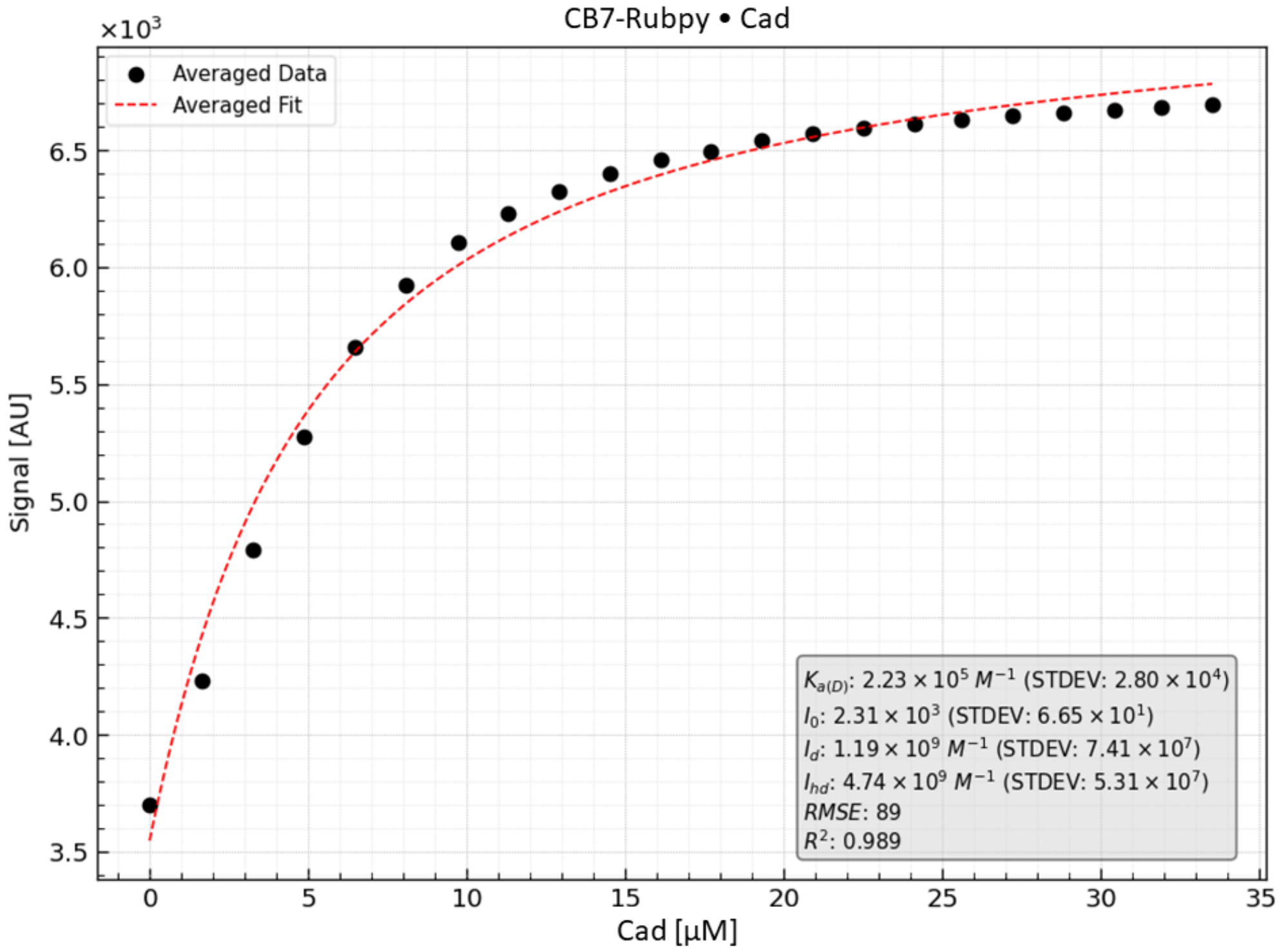

Figure S47. Titration curve and fit (1:1 binding model) of cadaverine with CB7-Rubpy in phosphate buffer (PB, 10 mM, pH 7.4). The emission intensity at 610 nm (λ_{exc} = 452 nm) is plotted as a function of cadaverine concentration ([CB7-Rubpy] = 1 μM; [cadaverine] = 0-30 μM). The dashed line represents the fit to a 1:1 binding model.

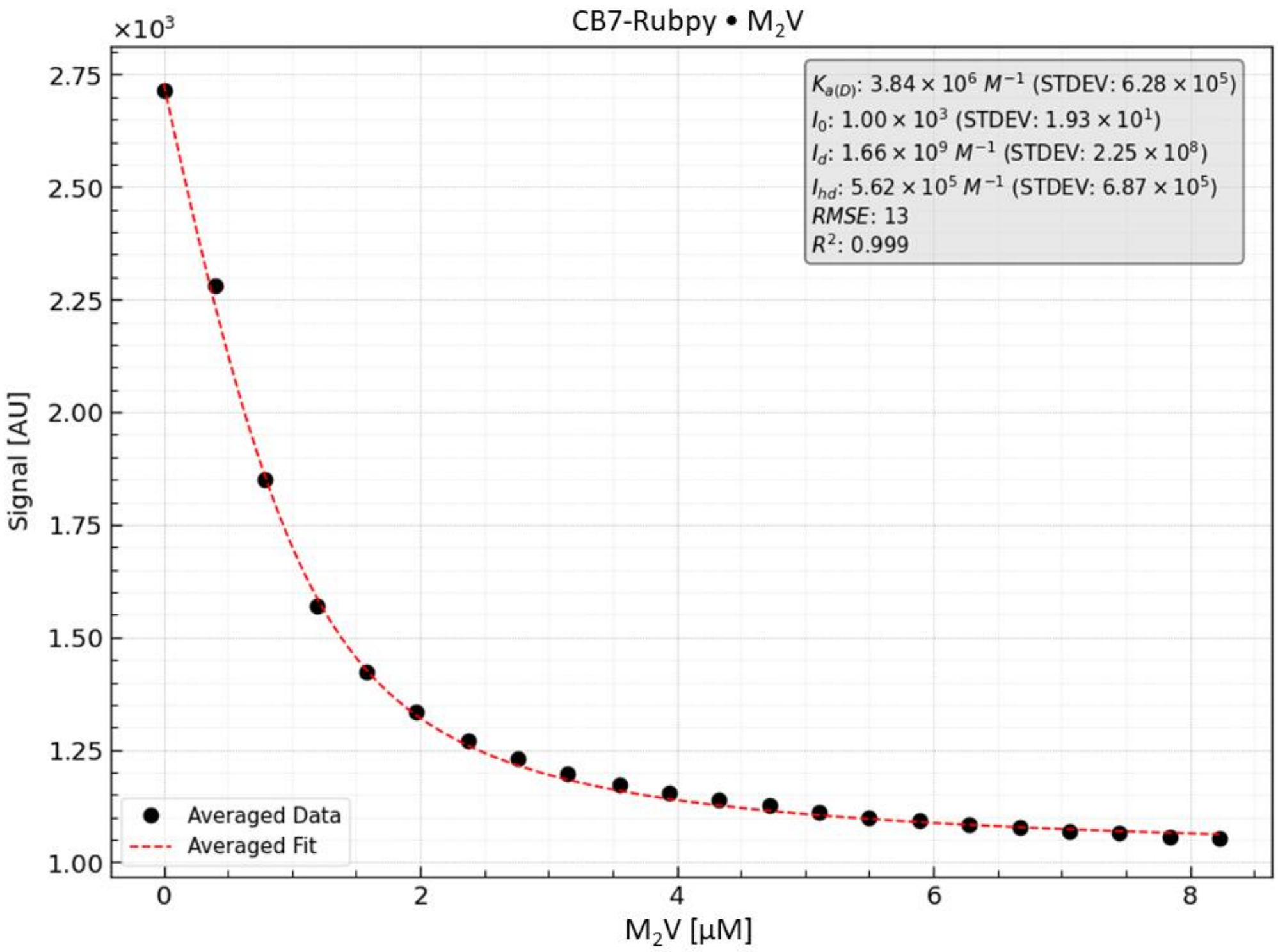

Figure S48. Titration curve and fit (1:1 binding model) of methyl viologen with CB7-Rubpy in phosphate buffer (PB, 10 mM, pH 7.4). The emission intensity at 610 nm (λ_{exc} = 452 nm) is plotted as a function of methyl viologen concentration ([CB7-Rubpy] = 1 μM; [methyl viologen] = 0-8 μM). The dashed line represents the fit to a 1:1 binding model.

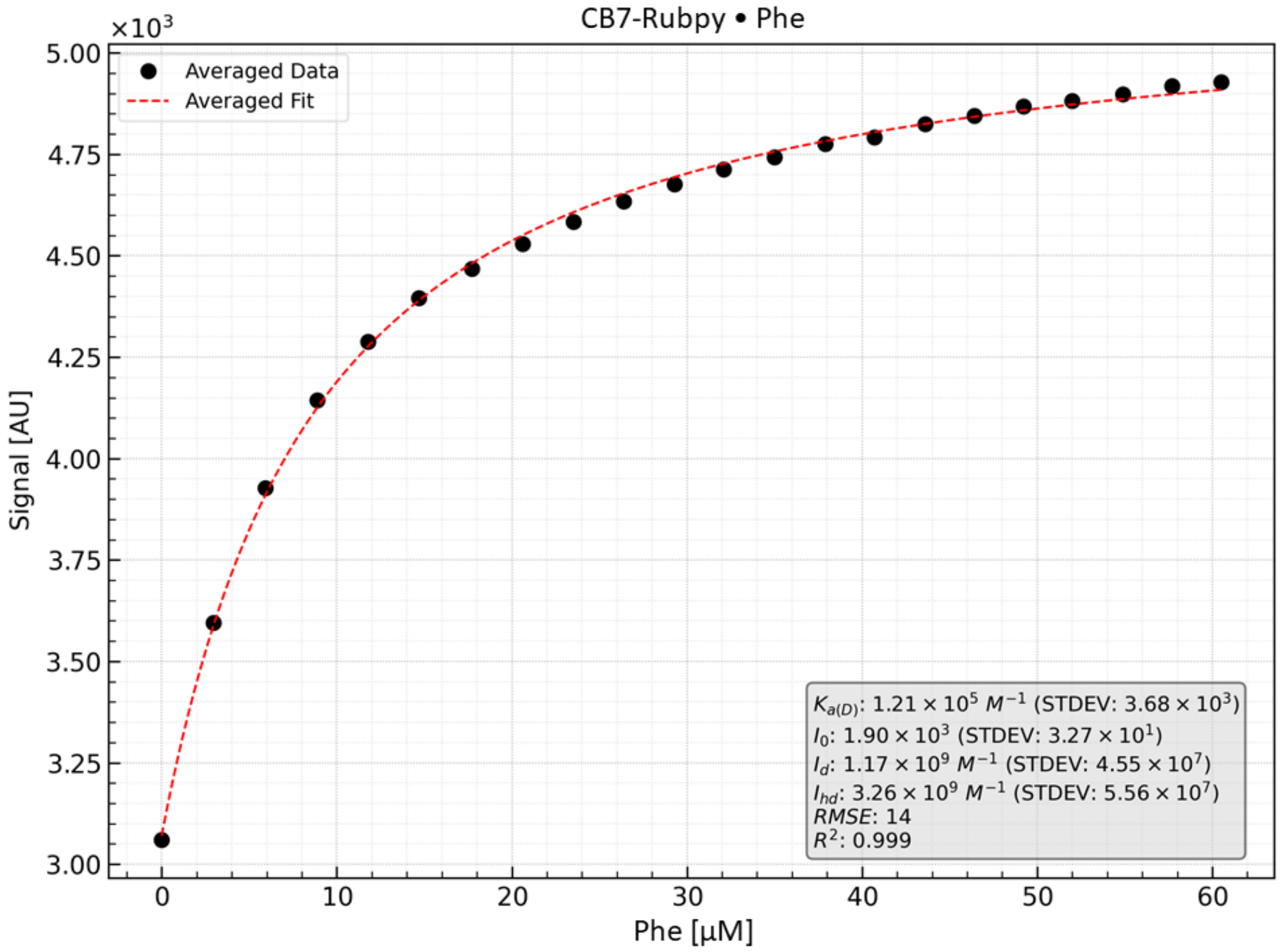

Figure S49. Titration curve and fit (1:1 binding model) of phenylalanine with CB7-Rubpy in phosphate buffer (PB, 10 mM, pH 7.4). The emission intensity at 610 nm (λ_{exc} = 452 nm) is plotted as a function of phenylalanine concentration ([CB7-Rubpy] = 1 µM; [phenylalanine] = 0-60 µM). The dashed line represents the fit to a 1:1 binding model.

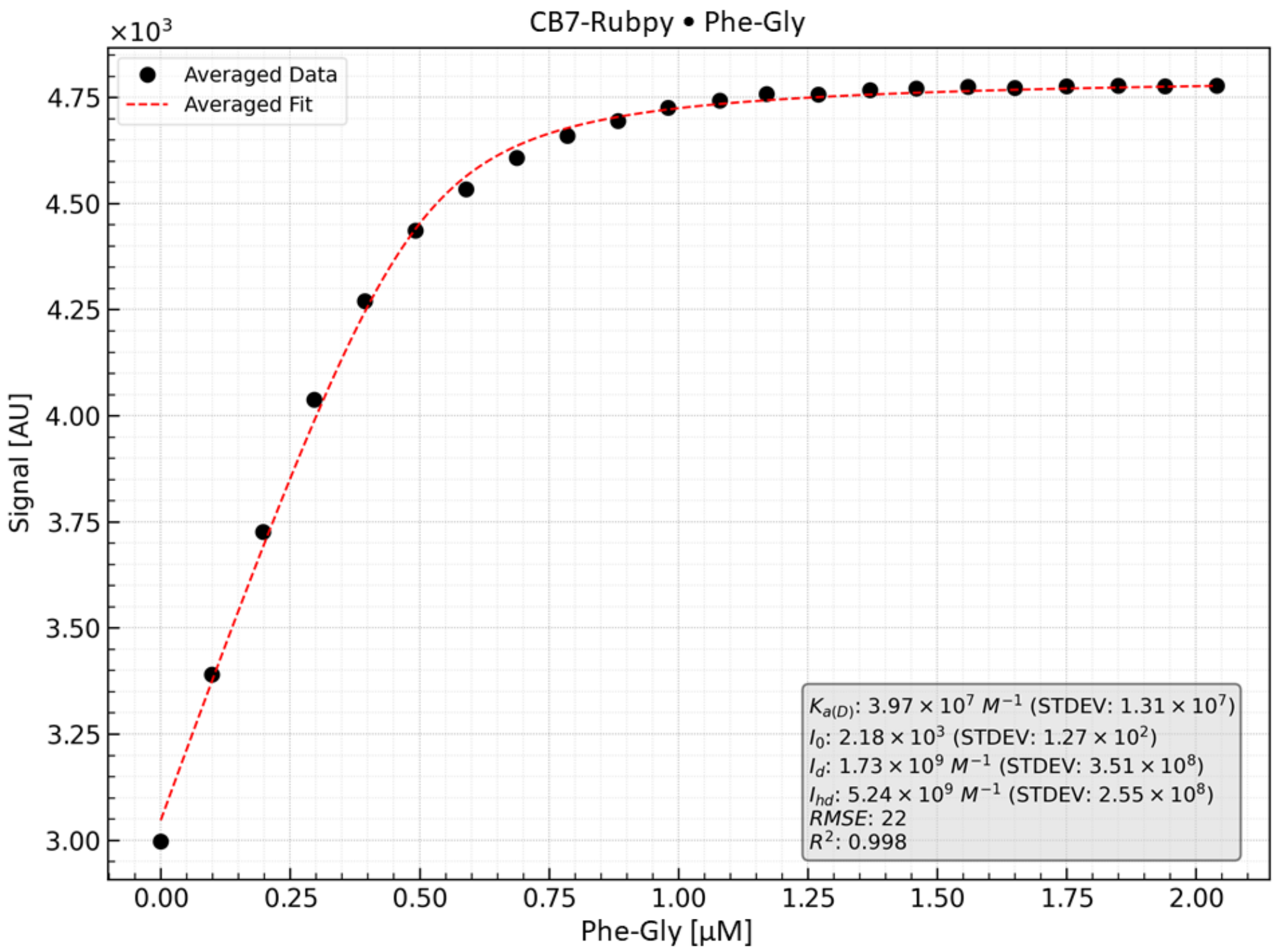

Figure S50. Titration curve and fit (1:1 binding model) of phenylalanyl-glycine (Phe-Gly) with CB7-Rubpy in phosphate buffer (PB, 10 mM, pH 7.4). The emission intensity at 610 nm (λ_{exc} = 452 nm) is plotted as a function of Phe-Gly concentration ([CB7-Rubpy] = 1 µM; [Phe-Gly] = 0-2 µM). The dashed line represents the fit to a 1:1 binding model.

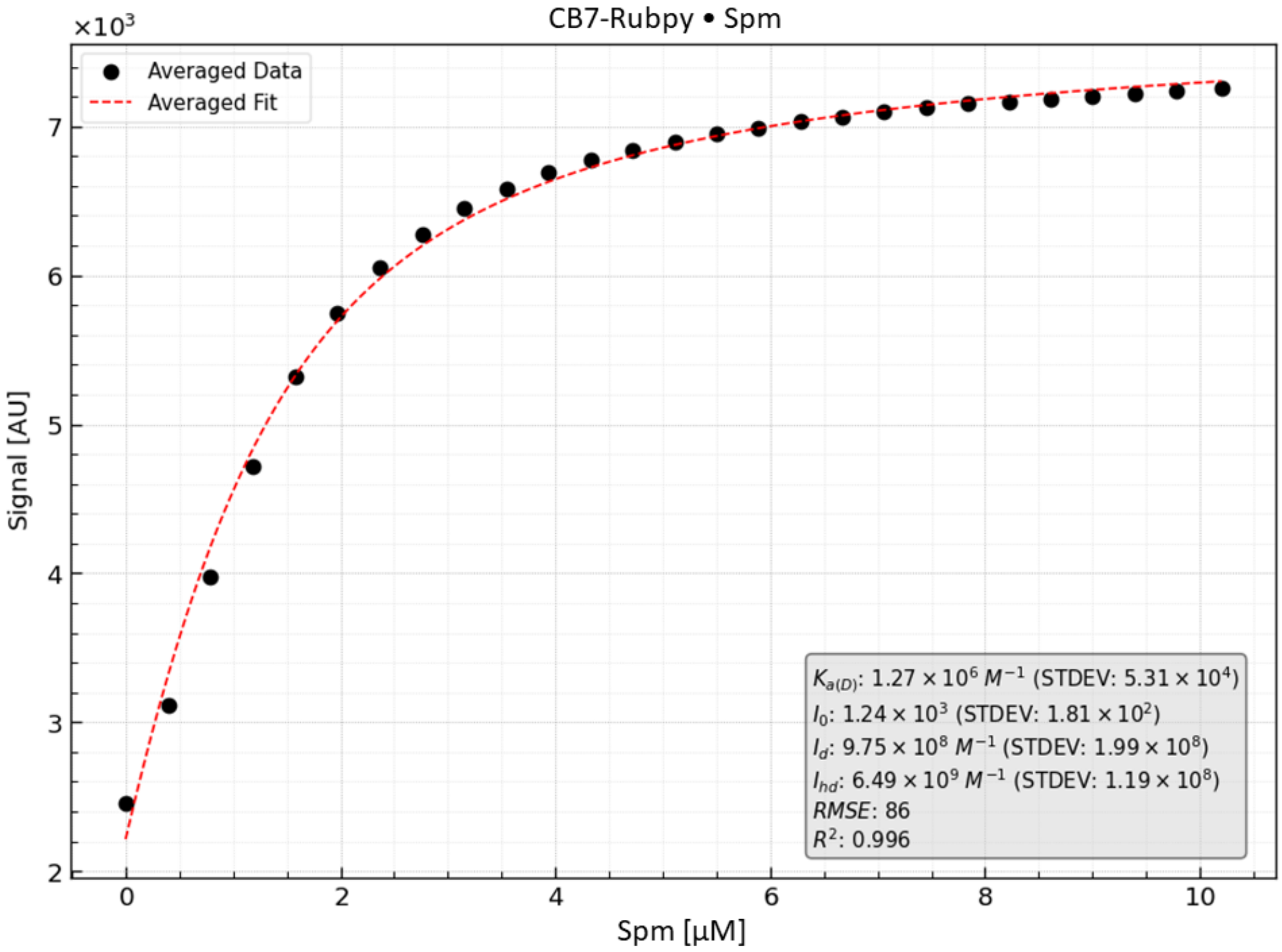

Figure S51. Titration curve and fit (1:1 binding model) of spermine (Spm) with CB7-Rubpy in phosphate buffer (PB, 10 mM, pH 7.4). The emission intensity at 610 nm (λ_{exc} = 452 nm) is plotted as a function of spermine concentration ([CB7-Rubpy] = 1 µM; [Spm] = 0-19 µM). The dashed line represents the fit to a 1:1 binding model.

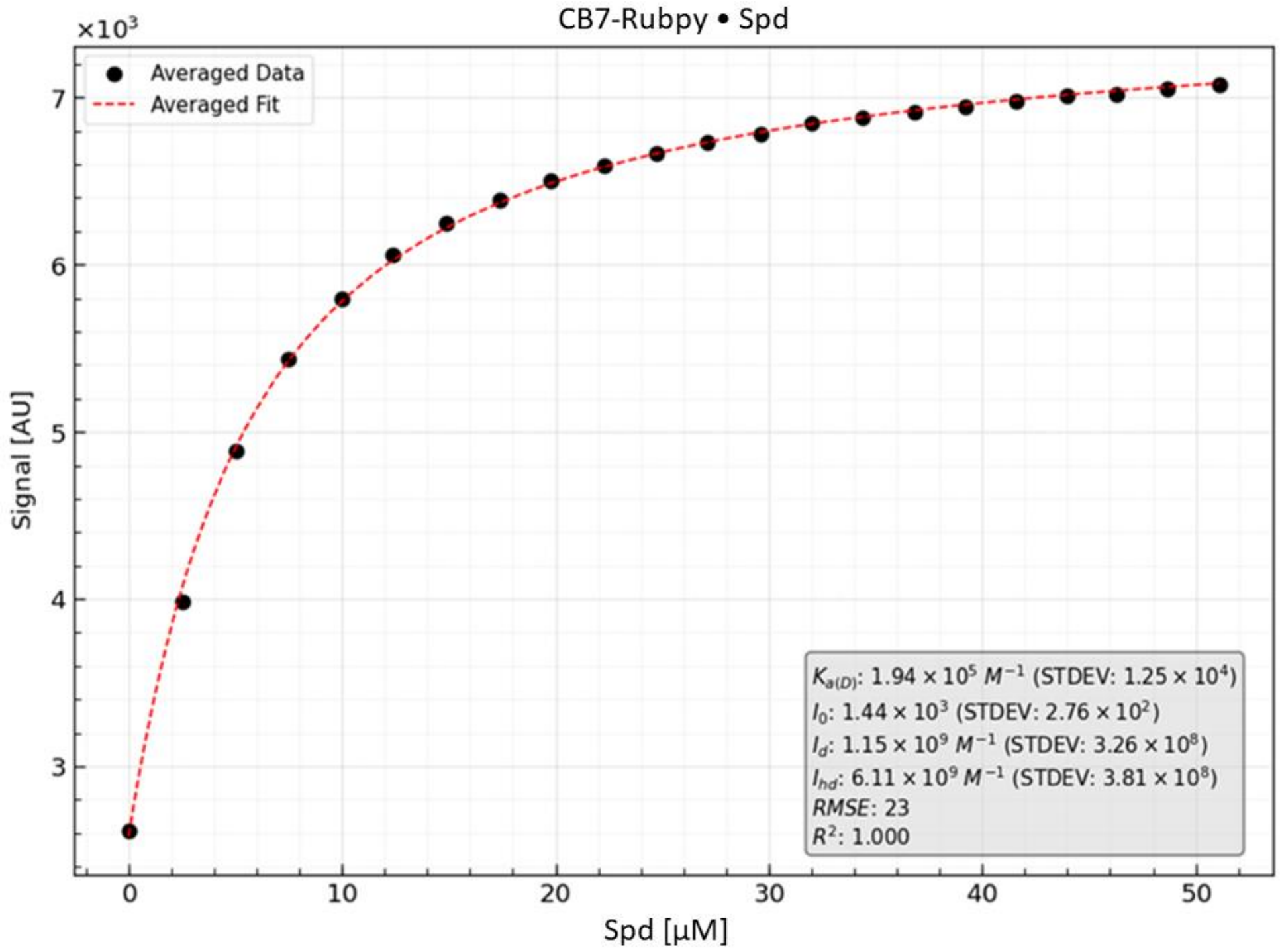

Figure S52. Titration curve and fit (1:1 binding model) of spermidine (Spd) with CB7-Rubpy in phosphate buffer (PB, 10 mM, pH 7.4). The emission intensity at 610 nm (λ_{exc} = 452 nm) is plotted as a function of spermidine concentration ([CB7-Rubpy] = 1 µM; [Spd] = 0-70 µM). The dashed line represents the fit to a 1:1 binding model.

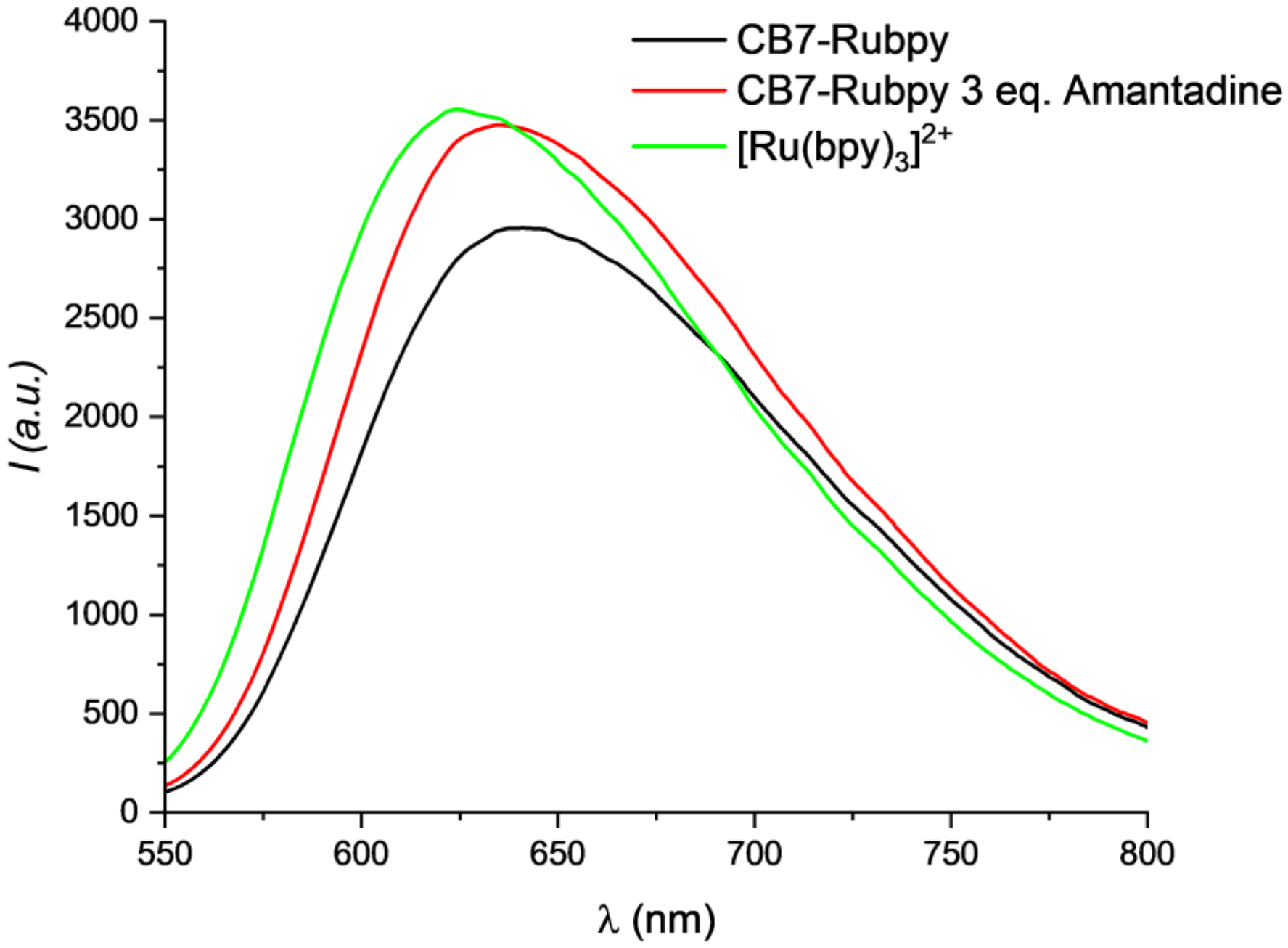

Figure S53. Emission spectra of CB7-Rubpy, CB7-Rubpy in the presence of excess amantadine, and $[Ru(bpy)_3]^{2+}$ (each 5 μM) in water.

All emission spectra were recorded with λ_{exc} = 452 nm, using an excitation slit width of 5 nm and an emission slit width of 2 nm, with an emission cut-off filter at 500 nm.

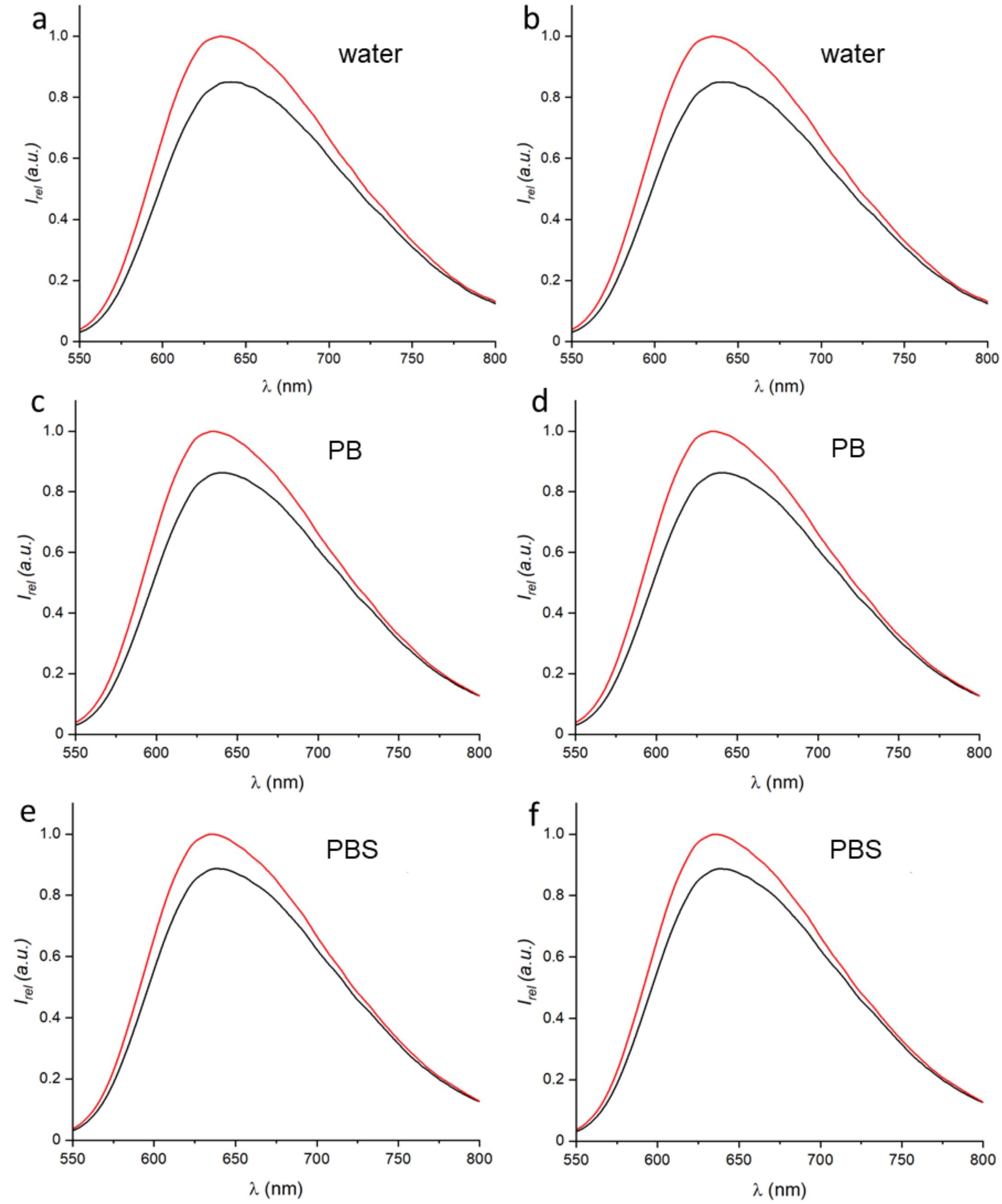

Figure S54. Comparison of the emission spectra (λ_{exc} = 452 nm) of CB7-Rubpy in the absence (0 equiv, black) and presence of amantadine (3 equiv, red) under aerated and degassed conditions in different aqueous media. (a) Aerated, water. (b) Degassed, water. (c) Aerated, PB. (d) Degassed, PB. (e) Aerated, PBS. (f) Degassed, PBS.

All emission spectra were recorded with an excitation wavelength of 452 nm, using an excitation slit width of 5 nm and an emission slit width of 2 nm, with an emission cut-off filter at 500 nm. Spectra were normalized to the maximum emission intensity of the highest-responding sample.

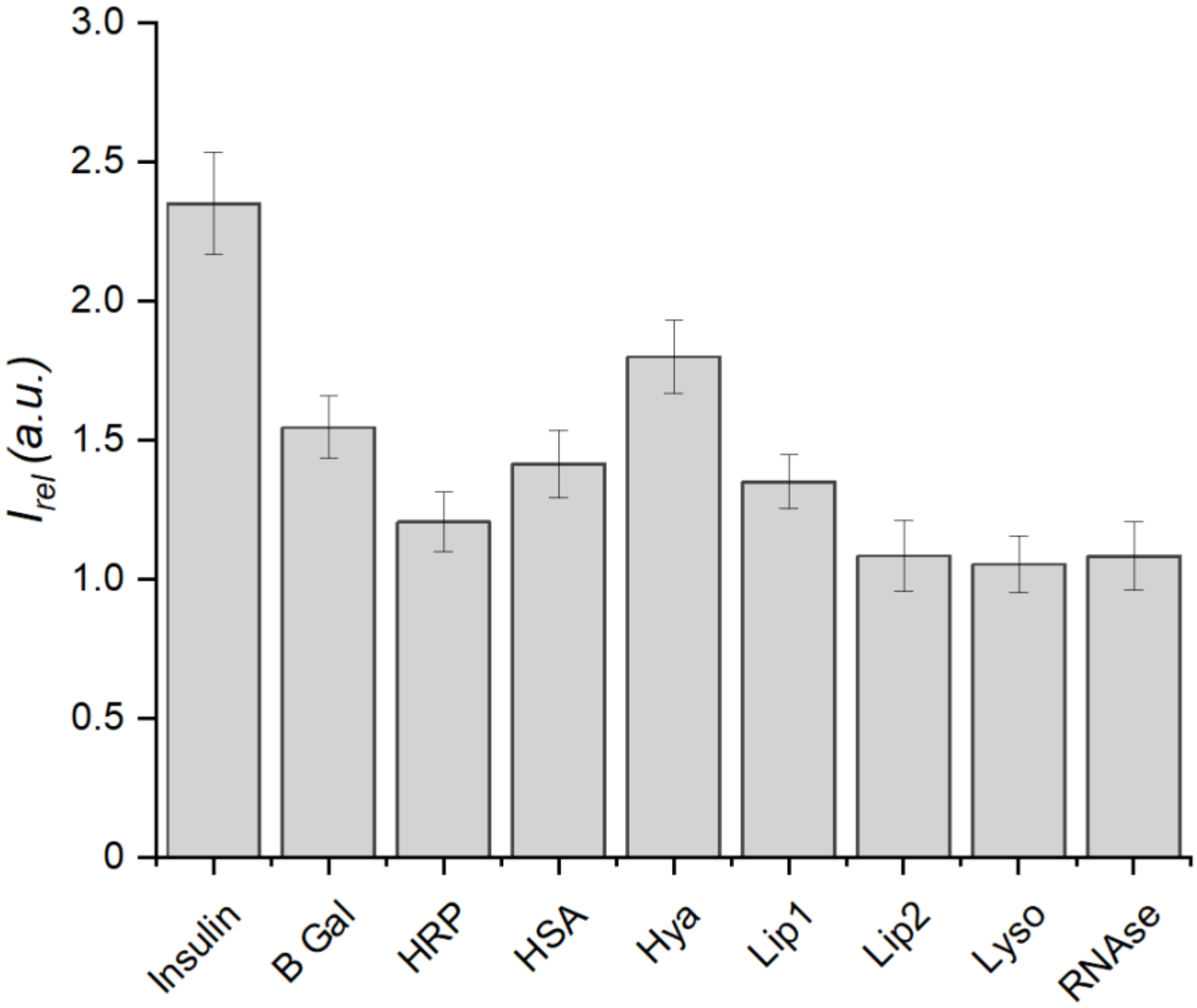

Figure S55. Comparison of the photoluminescent response of CB7-Rubpy toward different proteins in phosphate buffer (PB, 10 mM, pH 7.4). Emission intensities at 610 nm (λ_{exc} = 452 nm) of CB7-Rubpy ([CB7-Rubpy] = 5 μM) in the absence and presence of proteins (1 equiv) are shown under identical conditions. Data are normalized to the emission intensity of CB7-Rubpy in the absence of protein, highlighting protein-dependent differences in signal response.

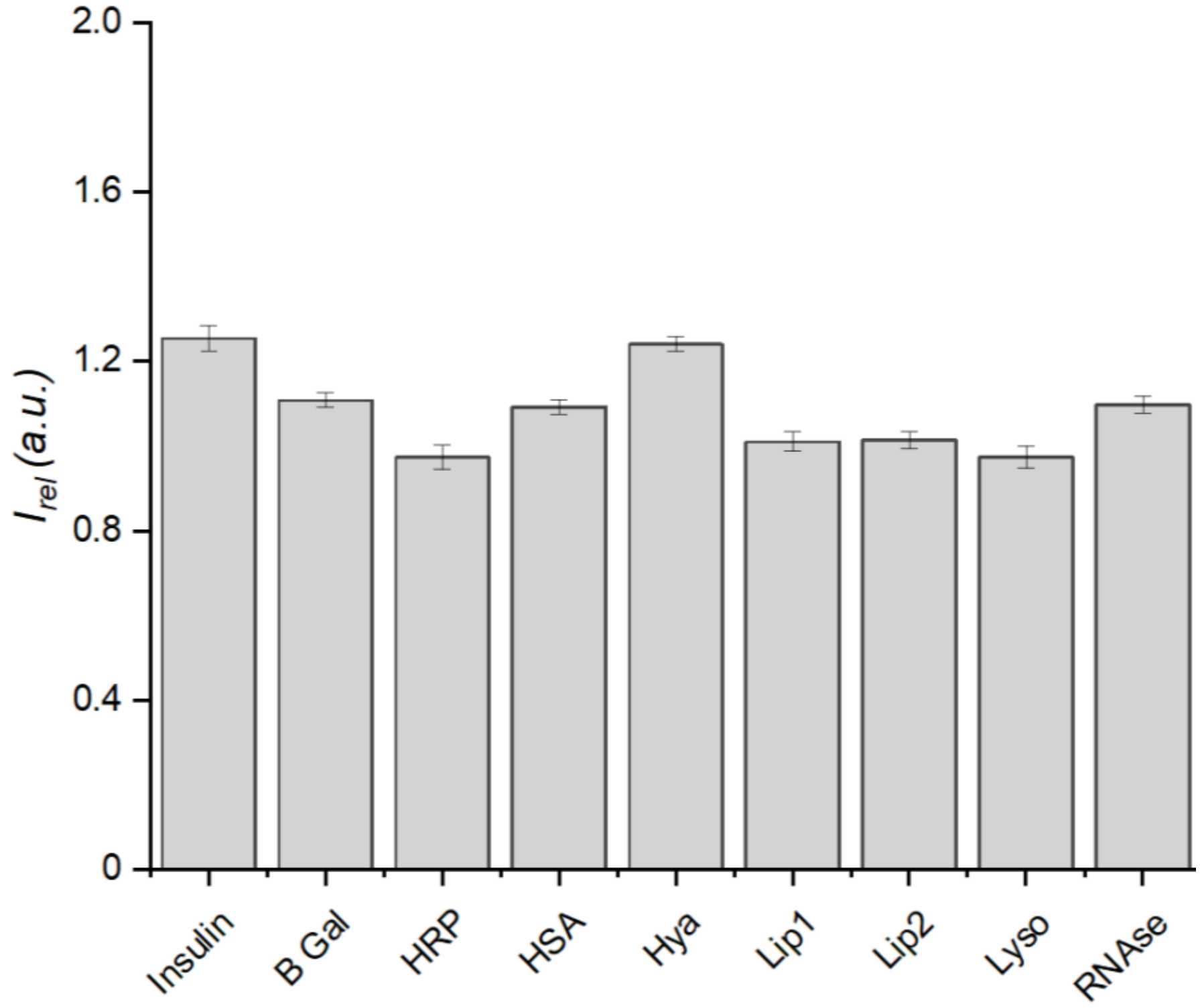

Figure S56. Comparison of the photoluminescent response of CB7-Rubpy toward different proteins in phosphate-buffered saline (PBS, 1×, pH 7.4). Emission intensities at 610 nm (λ_{exc} = 452 nm) of CB7-Rubpy ([CB7-Rubpy] = 5 μM) in the absence and presence of proteins (1 equiv) are shown under identical conditions. Data are normalized to the emission intensity of CB7-Rubpy in the absence of protein, highlighting protein-dependent differences in signal response.

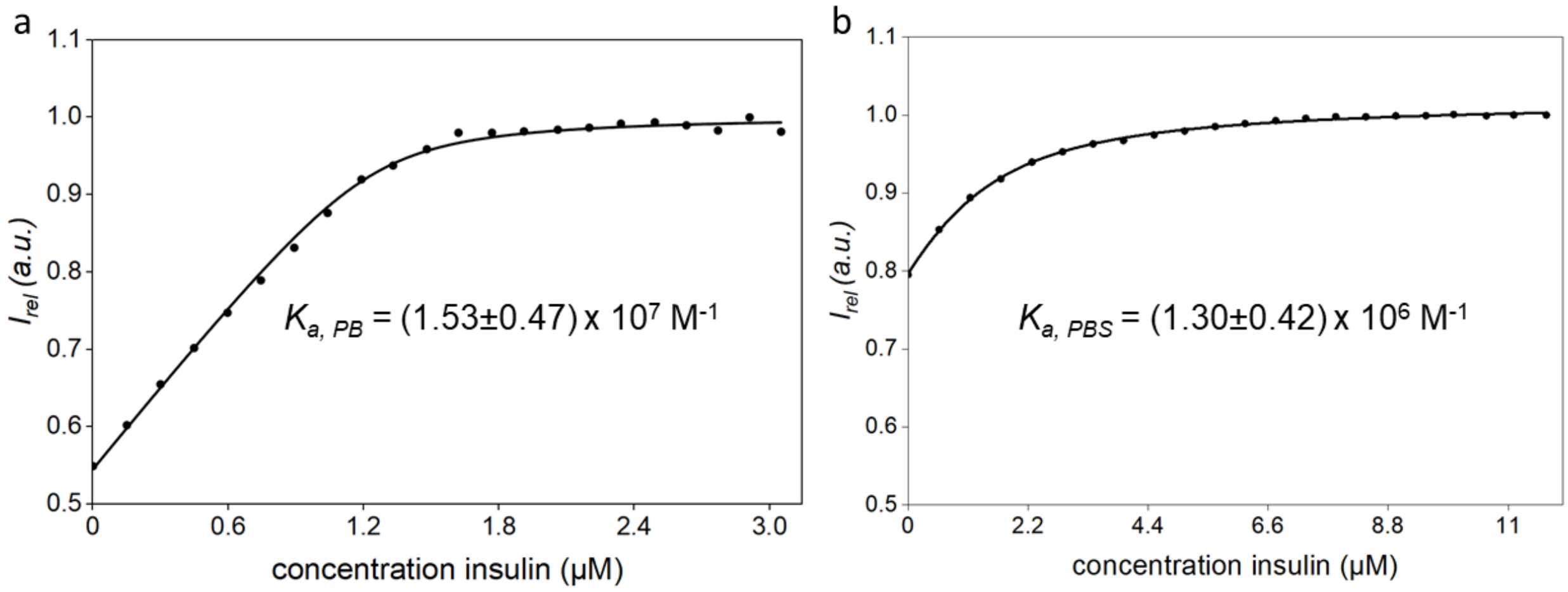

Figure S57. Determination of the binding affinity of CB7-Rubpy toward insulin by emission titration. Emission intensity (I_{rel}) of CB7-Rubpy (1 μM) recorded at λ_{exc} = 452 nm and λ_{em} = 610 nm upon titration with insulin in (a) phosphate buffer (PB, 10 mM, pH 7.4) and (b) phosphate-buffered saline (PBS, 1×, pH 7.4). Solid lines represent fits to a 1:1 binding model, yielding $K_{a,\ PB} = (1.53 \pm 0.47) \times 10^{7}\ M^{-1}$ and $K_{a,\ PBS} = (1.30 \pm 0.42) \times 10^{6}\ M^{-1}$. Each data point represents the average of three independent measurements (triplicates).

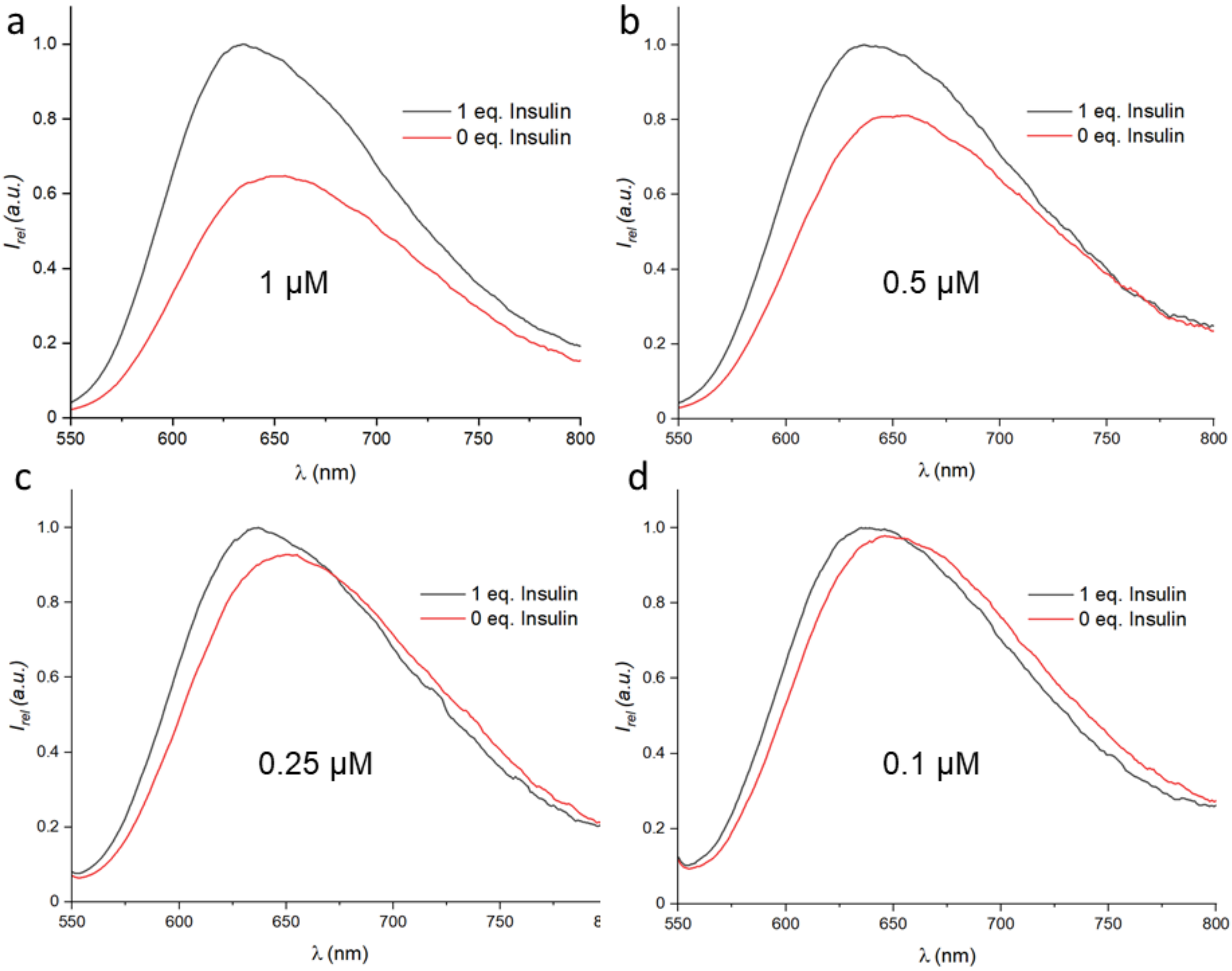

Figure S58. Concentration-dependent photoluminescent response of CB7-Rubpy toward insulin in phosphate buffer (PB, 10 mM, pH 7.4). Emission spectra (λ_{exc} = 452 nm) of CB7-Rubpy recorded in the absence (red) and presence (black) of insulin (1 equiv) at decreasing concentrations are shown for (a) 1 μM, (b) 0.5 μM, (c) 0.25 μM, and (d) 0.1 μM CB7-Rubpy/insulin. Pronounced differences in emission intensity are observed at 1 and 0.5 μM, while reduced but still measurable differences persist at 0.25 μM. At 0.1 μM, the intensity difference is small, although a modest spectral shift remains visible, indicating limited discrimination under these conditions. All emission spectra were recorded with an excitation slit width of 5 nm and an emission slit width of 2 nm, using an emission cut-off filter at 500 nm, and were normalized to the maximum emission intensity of the highest-response sample.

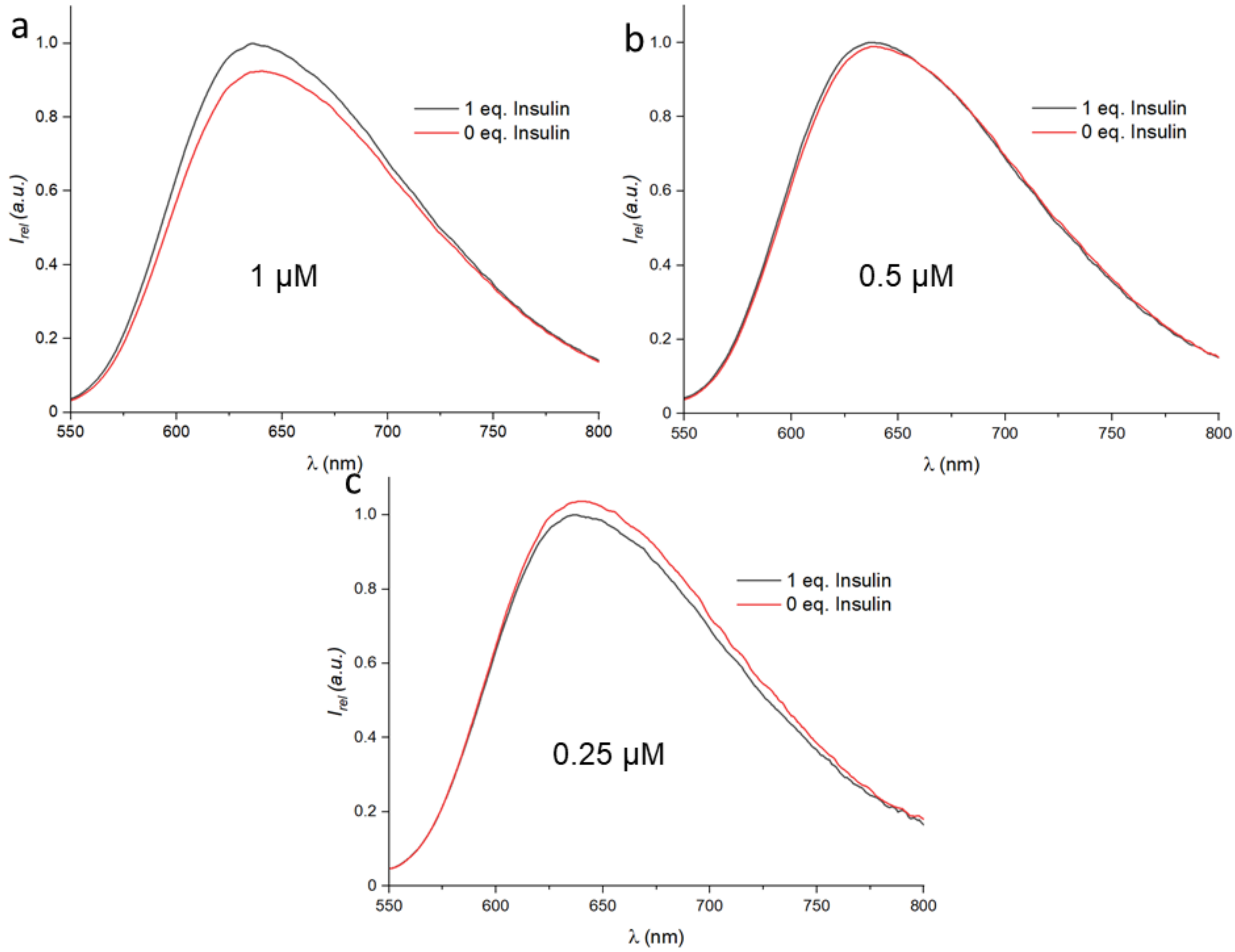

Figure S59. Concentration-dependent photoluminescent response of CB7-Rubpy toward insulin in phosphate-buffered saline (PBS, 1×, pH 7.4). Emission spectra (λ_{exc} = 452 nm) of CB7-Rubpy recorded in the absence (red) and presence (black) of insulin (1 equiv) at decreasing concentrations are shown for (a) 1 µM, (b) 0.5 µM, and (c) 0.25 µM CB7-Rubpy/insulin. In contrast to measurements in PB, only minor differences in emission intensity are observed at 1 µM and 0.5 µM, while the spectra largely overlap at lower concentrations, indicating limited discrimination under these conditions. All emission spectra were recorded with an excitation slit width of 5 nm and an emission slit width of 2 nm, using an emission cut-off filter at 500 nm, and were normalized to the maximum emission intensity of the highest-response sample.

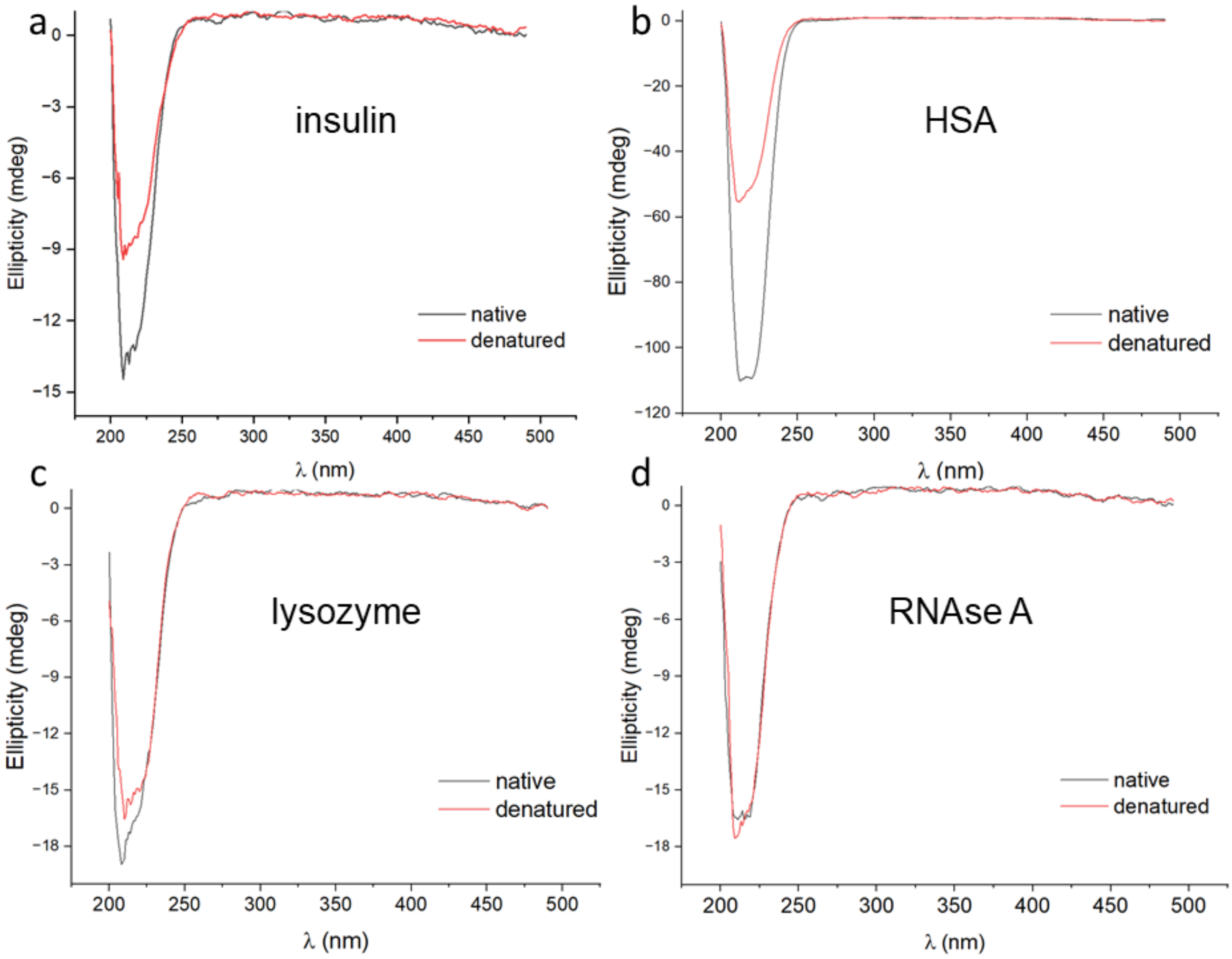

Figure S60. Circular dichroism (CD) spectra of proteins in phosphate buffer (PB, 10 mM, pH 7.4). CD spectra of (a) insulin, (b) human serum albumin (HSA), (c) lysozyme, and (d) RNase A are shown under native conditions, after chemical denaturation with urea/DTT, and after removal of the denaturants. For HSA, incomplete recovery of secondary structure is observed after denaturant removal, consistent with the enhanced CB7-Rubpy emission response after recovery. Insulin likewise shows altered CD signatures after chemical treatment, in agreement with the reduced emission response. In contrast, lysozyme and RNase A exhibit only minor changes in their CD spectra, consistent with the negligible differences observed in their emission responses between native and recovered states.

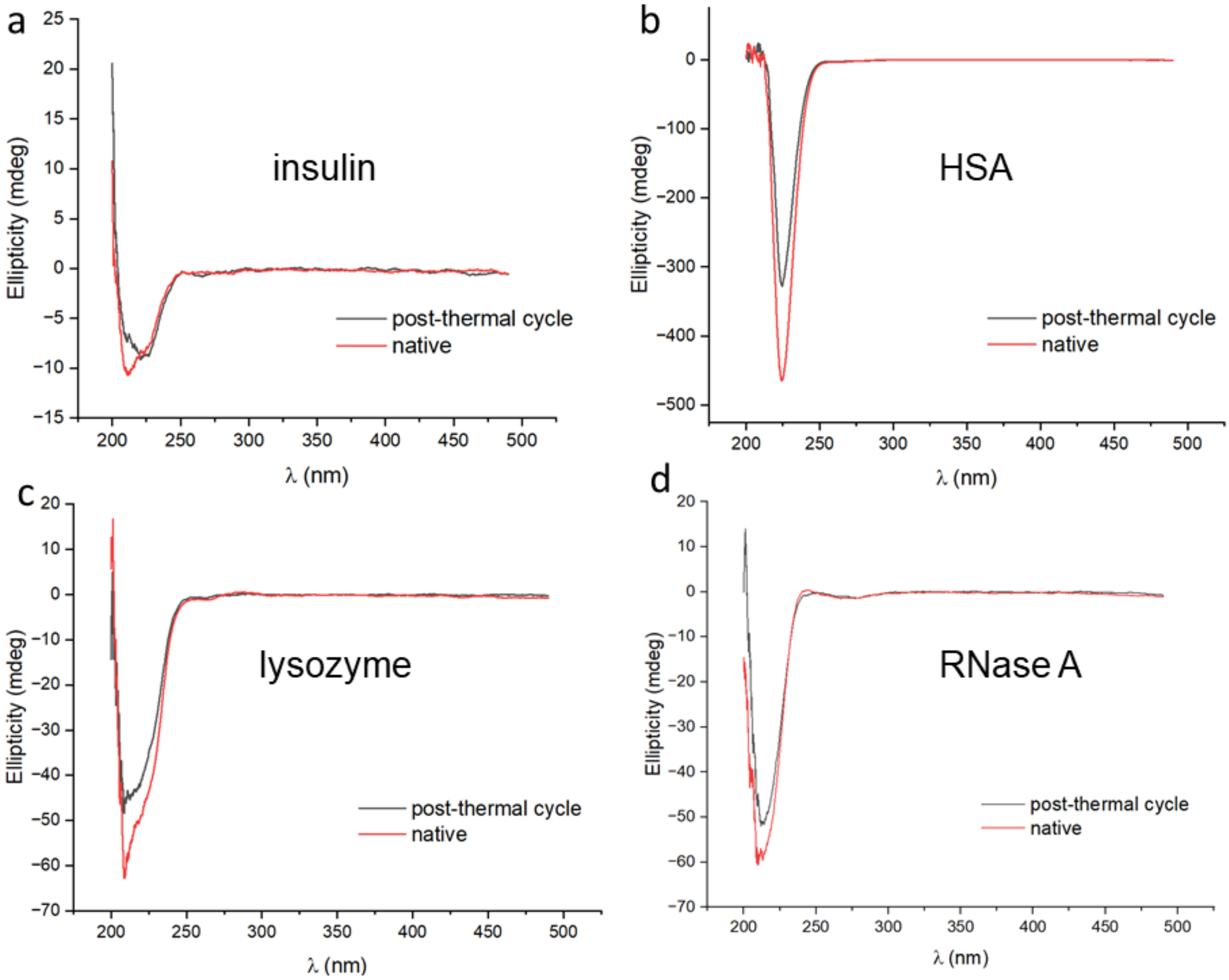

Figure S61. Circular dichroism (CD) spectra of proteins in phosphate buffer (PB, 10 mM, pH 7.4) following thermal denaturation and recovery. CD spectra of (a) insulin, (b) human serum albumin (HSA), (c) lysozyme, and (d) RNase A are shown under native conditions and after thermal denaturation (85 °C) followed by cooling to room temperature. HSA exhibits the largest changes in its CD spectrum, indicating incomplete structural recovery, consistent with the pronounced increase in CB7-Rubpy emission observed after thermal denaturation. Insulin likewise shows altered CD signatures after recovery, in agreement with the reduced emission response. In contrast, lysozyme and RNase A display only minor spectral changes, indicating efficient recovery of their native secondary structure and consistent with the negligible differences observed in their emission responses between native and thermally denatured states.

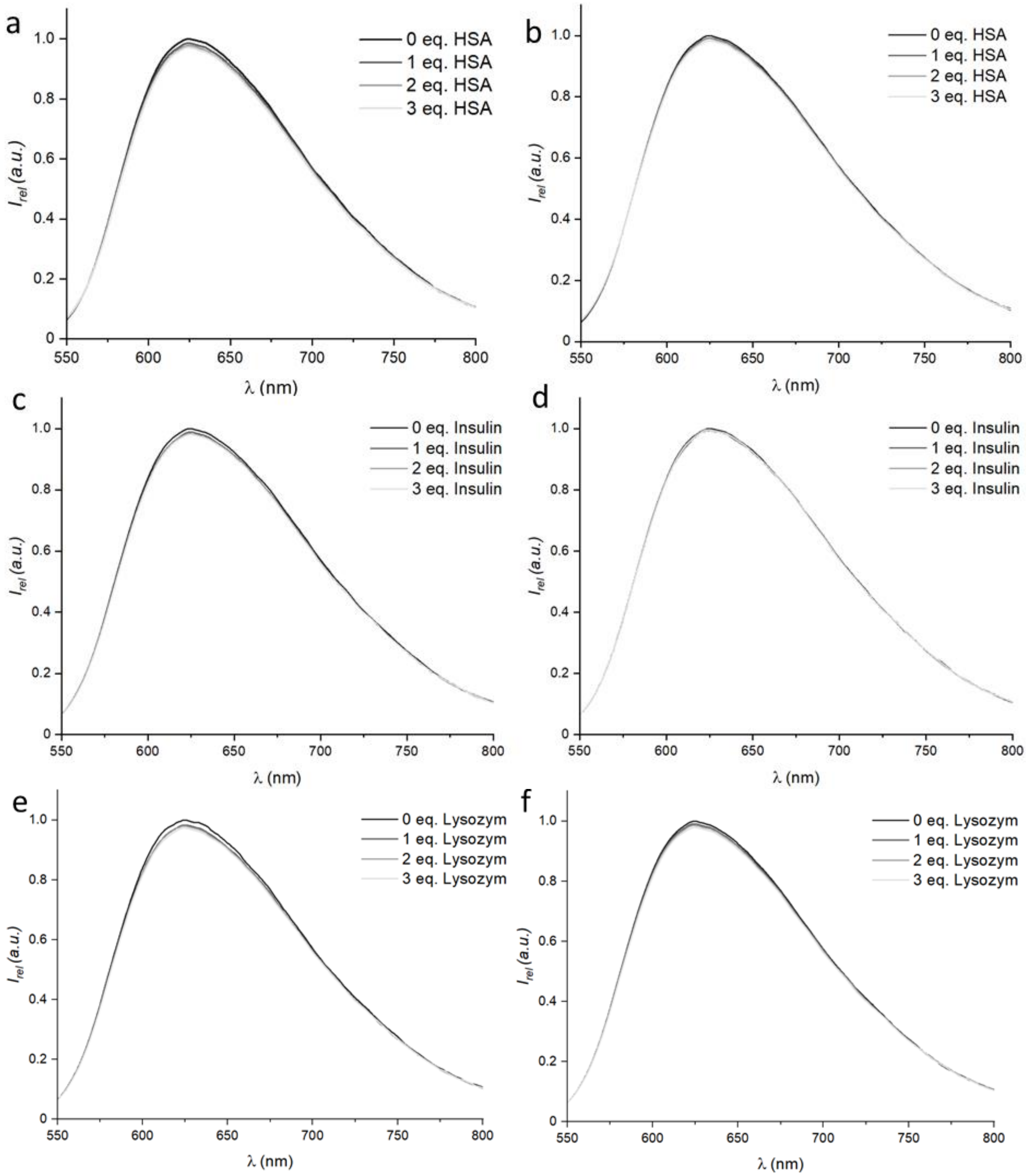

Figure S62. Emission spectra (λ_{exc} = 452 nm) of $[Ru(bpy)_3]Cl_2 \cdot 6H_2O$ (5 µM) in the absence and presence of proteins (5 µM) in different buffer systems: (a,b) human serum albumin (HSA), (c,d) insulin, and (e,f) lysozyme, measured in phosphate buffer (PB, 10 mM, pH 7.4) and phosphate-buffered saline (PBS, 1×, pH 7.4), respectively.

All emission spectra were recorded with an excitation slit width of 5 nm and an emission slit width of 2 nm, using an emission cut-off filter at 500 nm, and were normalized to the maximum emission intensity of the highest-response sample.

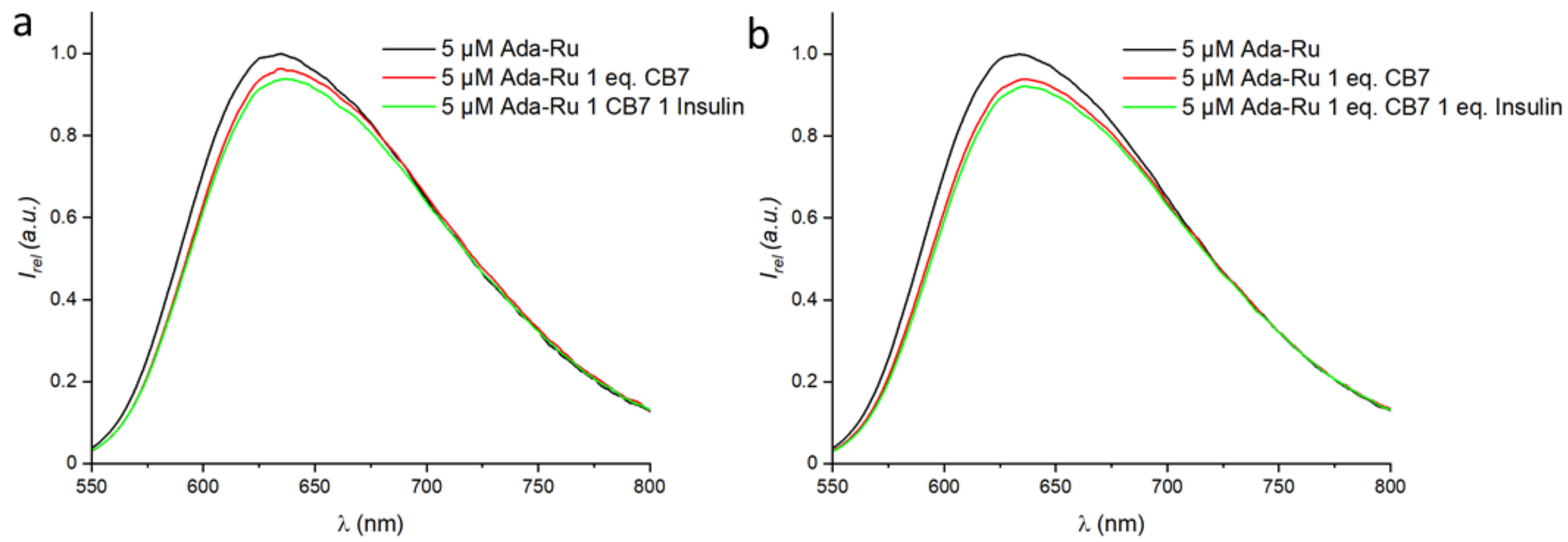

Figure S63. Emission spectra (λ_{exc} = 452 nm) of Ada-Ru (5 μM), Ada-Ru (5 μM) in the presence of CB7 (5 μM), and Ada-Ru (5 μM) with CB7 (5 μM) upon addition of insulin (5 μM) in (a) phosphate buffer (PB, 10 mM, pH 7.4) and (b) phosphate-buffered saline (PBS, 1×, pH 7.4).

All emission spectra were recorded with an excitation slit width of 5 nm and an emission slit width of 2 nm, using an emission cut-off filter at 500 nm, and were normalized to the maximum emission intensity of the highest-response sample.

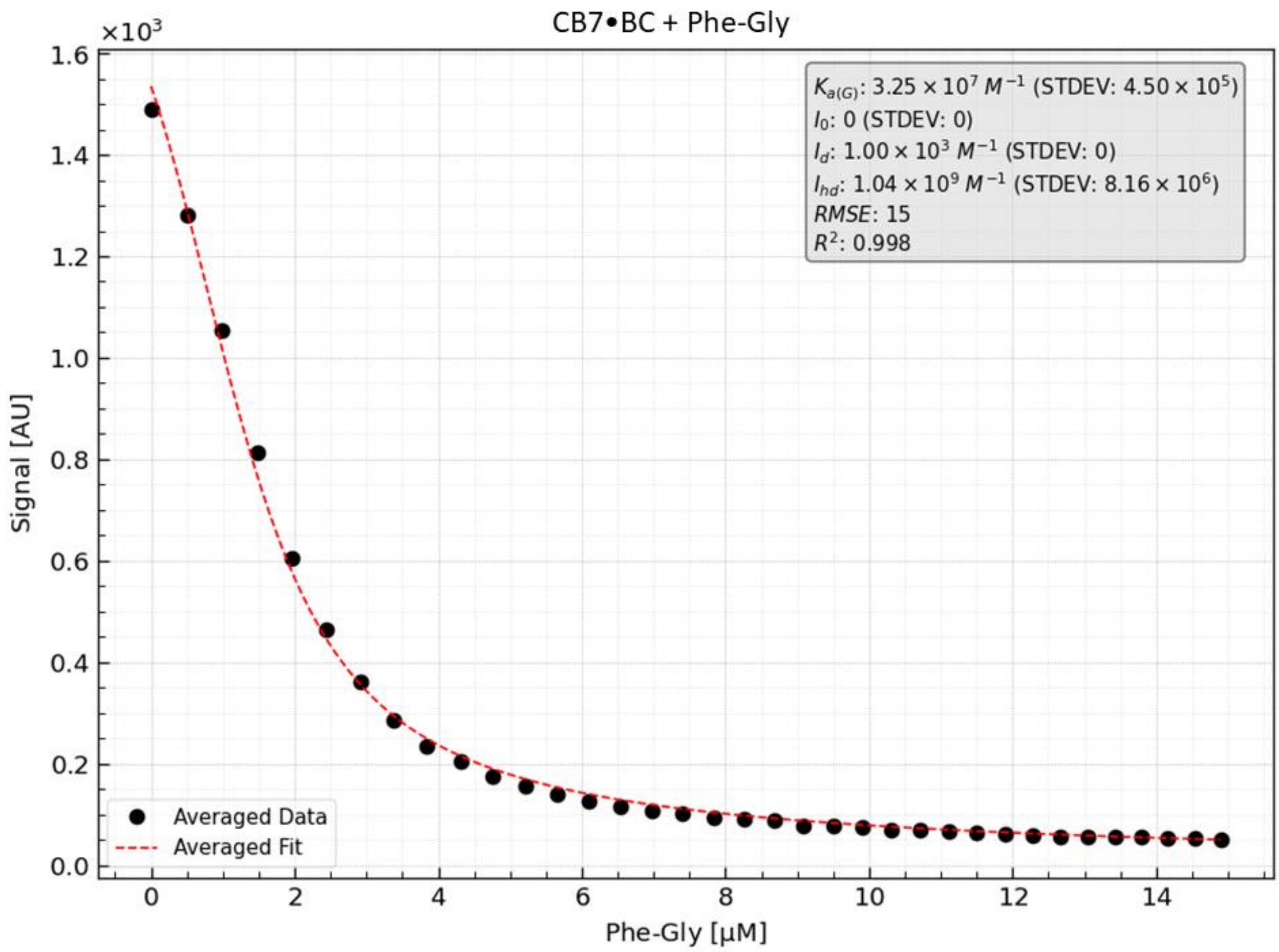

Figure S64. Indicator displacement assay (IDA) for phenylalanyl-glycine (Phe-Gly) using the CB7•berberine chloride reporter pair in water. The assay was performed with 2 μM CB7 and 2 μM berberine chloride by titration with increasing concentrations of Phe-Gly. Berberine emission was recorded using λ_{exc} = 345 nm and λ_{em} = 487 nm, and the resulting displacement response was used to determine the binding affinity of Phe-Gly to native CB7 under the same aqueous conditions used for the CB7-Rubpy binding studies. The dashed line represents the 1:1 displacement fit, yielding log K_a = 7.51 ± 0.01 for Phe-Gly binding to native CB7.

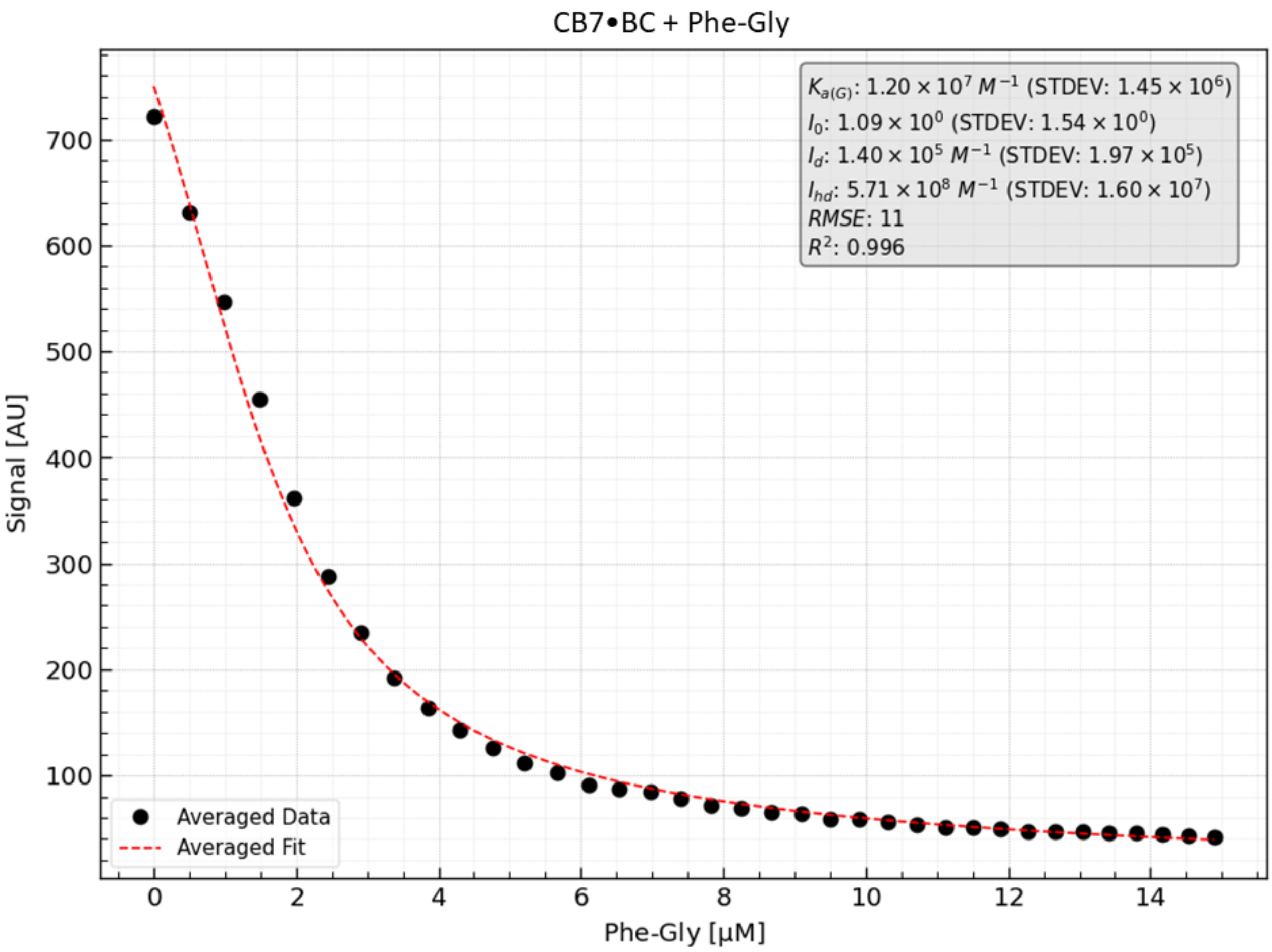

Figure S65. Indicator displacement assay (IDA) for phenylalanyl-glycine (Phe-Gly) using the CB7•berberine chloride reporter pair in PB (10 mM, pH 7.4). The assay was performed with 2 μM CB7 and 2 μM berberine chloride by titration with increasing concentrations of Phe-Gly. Berberine emission was recorded using λ_{exc} = 345 nm and λ_{em} = 487 nm, and the resulting displacement response was used to determine the binding affinity of Phe-Gly to native CB7 under the same aqueous conditions used for the CB7-Rubpy binding studies. The dashed line represents the 1:1 displacement fit, yielding log K_a = 7.08 ± 0.05 for Phe-Gly binding to native CB7.

6. Supporting References

(1) Marquez, C. H., F.; Nau, W. M. Cucurbiturils: Molecular Nanocapsules for Time-Resolved Fluorescence-Based Assays. *IEEE Trans Nanobioscience* **2004**, *3* (1), 39-45. DOI: https://doi.org/10.1109/TNB.2004.824269.
(2) Dong, N. H., J.; Li, T.; Peralta, A.; Avei, M. R.; Ma, M.; Kaifer, A. E. . Synthesis and Binding Properties of Monohydroxycucurbit[7]uril: A Key Derivative for the Functionalization of Cucurbituril Hosts. *J. Org. Chem.* **2018**, *83* (10), 5467-5473. DOI: https://doi.org/10.1021/acs.joc.8b00382.
(3) Koc, A. K., R.; Tuncel, D. "Clicked" Porphyrin-Cucurbituril Conjugate: A New Multifunctional Supramolecular Assembly Based on Triglycosylated Porphyrin and Monopropargyloxycucurbit[7]uril. *Chem. Eur. J.* **2018**, *24* (58), 15550-15555. DOI: https://doi.org/10.1002/chem.201804024.
(4) Weis, P. H., F.; Fischer, R.; Schneider, E. K.; Neumaier, M.; Kappes, M. M. . Probing the structure of giant fullerenes by high resolution trapped ion mobility spectrometry. *Phys. Chem. Chem. Phys.* **2019**, *21* (35), 18877-18892. DOI: https://doi.org/10.1039/C9CP03326B
(5) Stow, S. M. C., T. J.; Zheng, X.; Kurulugama, R. T.; Mairinger, T.; May, J. C.; Rennier, E. E.; Baker, E. S.; Smith, R. D.; McLean, J. A.; Hann, S.; Fjeldsted, J. C. . An Interlaboratory Evaluation of Drift Tube Ion Mobility–Mass Spectrometry Collision Cross Section Measurements. *Anal. Chem* **2017**, *89* (17), 9048-9055. DOI: https://doi.org/10.1021/acs.analchem.7b01729.
(6) Pracht, P. B., F.; Grimme, S. . Automated exploration of the low-energy chemical space with fast quantum chemical methods. *Phys. Chem. Chem. Phys.* **2020**, *22* (14), 7169-7192 DOI: https://doi.org/10.1039/C9CP06869D
(7) Bannwarth, C. E., S.; Grimme, S. GFN2-xTB—An Accurate and Broadly Parametrized Self-Consistent Tight-Binding Quantum Chemical Method with Multipole Electrostatics and Density-Dependent Dispersion Contributions. *J. Chem. Theory Comput.* **2019**, *15* (3), 1652–1671. DOI: https://doi.org/10.1021/acs.jctc.8b01176.
(8) Grimme, S. H., A.; Ehlert, S.; Mewes, J.-M. . r2SCAN-3c: An Efficient "Swiss Army Knife" Composite Electronic-Structure Method. *J. Chem. Phys.* **2021**, *154*. DOI: https://doi.org/10.1063/5.0040021.
(9) Zeglis, B. M. B., J. K. . A Mismatch-Selective Bifunctional Rhodium−Oregon Green Conjugate: A Fluorescent Probe for Mismatched DNA. *J. Am. Chem. Soc.* **2006**, *128* (17), 5654-5655. DOI: https://doi.org/10.1021/ja061409c.
(10) Fan, Z. W., J.; Liu, W.; Ma, J.; Sun, J.; Wang, P. Thiol-selective sensor based on intramolecular energy transfer between a bichromophoric system. *Tetrahedron* **2013**, *69* (23), 4536-4540. DOI: https://doi.org/10.1016/j.tet.2013.04.038.
(11) Hu, C. G., L.; Prabodh, A.; Baksi, A.; Siennicka, A.; Levkin, P. A.; Kappes, M. M.; Biedermann, F. Covalent Cucurbit[7]Uril–Dye Conjugates for Sensing in Aqueous Saline Media and Biofluids. *Chem. Sci.* **2020**, *11* (41), 11142–11153. DOI: https://doi.org/10.1039/D0SC03079A.
(12) Plett, C. G., S. . Automated and Efficient Generation of General Molecular Aggregate Structures. *Angew. Chem. Int. Ed.* **2022**, *62* (4). DOI: https://doi.org/10.1002/anie.202214477.
(13) Klamt, A. S., G. COSMO: a new approach to dielectric screening in solvents with explicit expressions for the screening energy and its gradient *J. Chem. Soc., Perkin Trans. 2* **1993**, (5), 799-805.
(14) Larriba-Andaluz, C. H., C. J. Jr. . Collision cross section calculations for polyatomic ions considering rotating diatomic/linear gas molecules. *J. Chem. Phys.* **2014**, *141* (19). DOI: https://doi.org/10.1063/1.4901890.
(15) Kim, H. K., H. I.; Johnson, P. V.; Beegle, L. W.; Beauchamp, J. L.; Goddard, W. A.; Kanik, I. . Experimental and Theoretical Investigation into the Correlation between Mass and Ion Mobility

for Choline and Other Ammonium Cations in N_2. *Anal. Chem* **2008**, *80* (6), 1928-1936. DOI: https://doi.org/10.1021/ac701888e.
(16) Hu, C. J., T.; Chakraborty, P.; Neumaier, M.; Levkin, P. A.; Kappes, M. M.; Biedermann, F. . Further Dimensions for Sensing in Biofluids: Distinguishing Bioorganic Analytes by the Salt-Induced Adaptation of a Cucurbit[7]Uril-Based Chemosensor. *J. Am. Chem. Soc.* **2022**, *144* (29), 13084–13095. DOI: https://doi.org/10.1021/jacs.2c01520.
(17) Jankowski, J. J. K., D. J.; Mopper, K. . Nitrate and Nitrite Ultraviolet Actinometers *Photochem. Photobiol.* **2008**, *70* (3), 319-328. DOI: 10.1111/j.1751-1097.1999.tb08143.x.
(18) Zhang, S. G., L.; Miskolczy, Z.; Biczók, L.; Biedermann, F.; Nau, W. M. Binding Affinities of Cucurbit[*n*]Urils with Cations. *Chem. Commun.* **2019**, *55* (94), 14131-14134. DOI: https://doi.org/10.1039/C9CC07687E.
(19) Sinn, S. K., J.; Biedermann, F. . Teaching old indicators even more tricks: binding affinity measurements with the guest-displacement assay (GDA). *Chem. Commun.* **2020**, *56* (49), 6620-6623. DOI: https://doi.org/10.1039/D0CC01841D.
(20) Vincil, G. A. U., A. Effects of the number and placement of positive charges on viologen-cucurbit[*n*}uril interactions *Supramol. Chem.* **2008**, *20* (8), 681-687. DOI: https://doi.org/10.1080/10610270701689572.
(21) Lee, E.-C. K., H.-J.; Park, S. Y. . Reversible Shape-Morphing and Fluorescence-Switching in Supramolecular Nanomaterials Consisting of Amphiphilic Cyanostilbene and Cucurbit[7]uril *Chem. Asian J.* **2019**, *14* (9), 1457-1461 DOI: https://doi.org/10.1002/asia.201900204.
(22) Nau, W. M. G., G.; Hennig, A.; Bakirci, H.; Bailey, D. M. . Substrate-Selective Supramolecular Tandem Assays: Monitoring Enzyme Inhibition of Arginase and Diamine Oxidase by Fluorescent Dye Displacement from Calixarene and Cucurbituril Macrocycles. *J. Am. Chem. Soc.* **2009**, *131* (32), 11558-11570. DOI: https://doi.org/10.1021/ja904165c.
(23) Cheng, Q. Y., Z.; Quan, X.; Ding, Y.; Li, J.; Wang, Z.; Zhao, Y.; Chen, X.; Wang, R. . Tumor polyamines as guest cues attract host-functionalized liposomes for targeting and hunting via a bio-orthogonal supramolecular strategy *Theranostics* **2023**, *13* (2), 611-620. DOI: 10.7150/thno.80857.
(24) Chen, Y. S., Z. Supramolecular Chemotherapy Based on the Host–Guest Complex of Lobaplatin–Cucurbit[7]uril. *ACS Appl. Bio Mater.* **2020**, *3* (4), 2449-2454. DOI: https://doi.org/10.1021/acsabm.0c00172.
(25) Grimm, L. M. S., J.; Tkachenko, B.; Schreiner, P. R.; Gilson, M. K.; Biedermann, F. The temperature-dependence of host–guest binding thermodynamics: experimental and simulation studies. *Chem. Sci.* **2023**, *14* (42), 11818-11829. DOI: https://doi.org/10.1039/D3SC01975F.
(26) Lee, J. W. L., H. H. L.; Ho Ko, Y.; Kim, K.; Kim, H. I. . Deciphering the Specific High-Affinity Binding of Cucurbit[7]uril to Amino Acids in Water *J. Phys. Chem. B* **2015**, *119* (13), 4628-4636. DOI: https://doi.org/10.1021/acs.jpcb.5b00743.
(27) Chinai, J. M. T., A. B.; Ryno, L. M.; Hargreaves, N. D.; Morris, C. A.; Hart, P. J.; Urbach, A. R. . Molecular Recognition of Insulin by a Synthetic Receptor *J. Am. Chem. Soc.* **2011**, *133* (23), 8810-8813. DOI: https://doi.org/10.1021/ja201581x.
